# Supplementary material for: Prehospital identification of intracerebral haemorrhage: a scoping review of early clinical features and portable devices
Source: BMJ Open. 2024 Apr 19;14(4):e079316. doi: 10.1136/bmjopen-2023-079316 (PMC11033659; doi:10.1136/bmjopen-2023-079316)
Supplement: Supplementary data [file bmjopen-2023-079316supp001.pdf]

Supplementary Table 1. Characteristics of the included studies for clinical features.

Included study, year and country	Design	Setting	Study population	Statistical method	Age (Years)	Onset-to-evaluation	Method of confirmation	Clinical features collected			Findings relevant to review
								Past medical history	Signs and symptoms	Vital signs	
Woisetschläger [25], 2000; Austria	Retrospective-prospective study	Prehospital and in-hospital	ICH, IS<br><br>ICH: 118<br>IS: 106	Uni- and multi-variable regression	ICH (mean): 58<br>IS (mean): 65	NR (Evaluated in prehospital)	CT scan	• AF<br>• Aspirin use<br>• Dicoumarin use<br>• DM<br>• HTN<br>• Hyperlipidaemia<br>• Prior MI<br>• Smoking	• Aphasia<br>• Headache<br>• Hemisymptoms<br>• Impaired LOC<br>• Seizures<br>• Syncope<br>• Vomiting	• SBP<br>• DBP<br>• ECG	• ICH patients were younger than IS patients.<br>• A history of HTN, DM, and MI was more commonly observed in IS patients.<br>• Impaired LOC was predictive of ICH.<br>• Unilateral weakness or sensory loss was observed more frequently in IS patients.
Yamashita [26], 2011; Japan	Retrospective study	Prehospital and in-hospital	ICH, IS<br><br>ICH: 100<br>IS: 127	Uni- and multi-variable regression	ICH (median): 68<br>IS (median): 74	Within 6 h of onset	CT or MRI scan	• ACS<br>• AF<br>• Antithrombotics use<br>• DM<br>• Haemodialysis<br>• HTN<br>• Hyperlipidaemia<br>• Prior TIA, IS, and ICH<br>• Smoking	• Headache<br>• Neurological deficits using NIHSS items<br>• Seizures<br>• Vomiting	• SBP<br>• DBP<br>• ECG	• ICH patients were younger than IS patients.<br>• Patients with ICH were more likely to have a history of ICH and haemodialysis, while patients with IS had a higher likelihood of a history of ACS, AF, antithrombotic usage, and hyperlipidaemia.<br>• Headache, vomiting, impaired LOC, and seizures were more frequently observed in the ICH group.<br>• The NIHSS median score was higher in the ICH group (16 vs 9).<br>• The median BP was higher in the ICH group, including both SBP (190 vs 164 mm Hg) and DBP (102 vs 90 mm Hg).
Jin [27], 2016; China	Prospective study	Prehospital and in-hospital	ICH, IS, SAH, non-stroke<br><br>ICH: 689<br>IS: 797<br>SAH: 109<br>Non-stroke: 394	Uni- and multi-variable regression	All patients (mean): 66	NR (Evaluated in prehospital)	CT or MRI scan	• AF<br>• DM<br>• HTN	• Vomiting	• SBP<br>• DBP<br>• ECG<br>• HR	• The comparison was between two groups: ICH vs IS/TIA.<br>• Age <65 years was found to be associated with the ICH group.<br>• IS/TIA patients were more likely to have a history of AF and DM.<br>• Vomiting was a common presenting symptom in the ICH group.<br>• SBP ≥180 mm Hg and DBP ≥100 mm Hg were more frequently observed in ICH patients, while arrhythmia was a common finding in the IS/TIA group.

Uchida [28], 2018; Japan	Prospective study	Prehospital and in-hospital	Any stroke, ICH, LVO, SAH, non-stroke<br><br>DC: 1229<br>Any stroke: 203<br>ICH: 169<br>LVO: 104<br>SAH: 57<br>Non-stroke: 696<br><br>VC: 1007<br>Any stroke: 253<br>ICH: 183<br>LVO: 131<br>SAH: 50<br>Non-stroke: 390	Uni- and multi-variable regression	DC (median): 72<br><br>VC (median): 75	NR (Evaluated in prehospital)	CT or MRI scan	• Antiplatelet use<br>• DOAC use<br>• Prior IS, ICH, and SAH<br>• Smoking<br>• Warfarin use	• Anisocoria<br>• Aphasia<br>• Conjugate deviation<br>• Convulsion<br>• Dizziness<br>• Dysarthria<br>• Facial palsy<br>• Headache<br>• Impaired LOC<br>• Nausea or vomiting<br>• Numbness<br>• Paralysis of lower limbs<br>• Paralysis of upper limbs<br>• Sudden onset<br>• Symptoms improved after onset<br>• Symptoms progressed after onset<br>• Unilateral spatial neglect	• SBP<br>• DBP<br>• ECG	• Patients with ICH were less likely to have a history of IS.<br>• Improvement following the onset of symptoms was associated with a decreased probability of ICH.<br>• Headache, dysarthria, paralysis of the upper limbs, conjugate deviation, and impaired LOC were common presentations of ICH. However, most of these symptoms were not specific.<br>• SBP ≥165 mm Hg and DBP ≥95 mm Hg were more frequently observed in ICH patients, while the presence of arrhythmia was associated with the absence of ICH.
Geisler [29], 2021; Germany	Retrospective study	Prehospital and in-hospital	ICH, IS, TIA, SM<br><br>DC: 416<br>ICH: 32<br>IS: 224<br>TIA: 41<br>SM: 119<br><br>VC: 285<br>ICH: 33<br>IS: 252	Uni- and multi-variable regression	DC (mean):<br>ICH: 71.5<br>IS: 74.9<br><br>VC (mean):<br>ICH: 73.3<br>IS: 73.9	≤4 h or unknown	CT scanner in MSU	• AF<br>• HTN	• Neurological deficits using NIHSS items<br>• Seizures	• SBP<br>• DBP<br>• MAP	• In the derivation set (ICH vs IS), ICH patients were more likely to be male.<br>• AF was found less frequently in the derivation set for the ICH group.<br>• ICH patients had higher median NIHSS scores in both sets (≥15).<br>• A greater number of ICH patients showed a decrease in LOC.<br>• The majority of seizures were found in the SM group.<br>• A higher proportion of ICH patients exhibited SBP ≥180 mm Hg, DBP ≥110 mm Hg, and MAP ≥130 mm Hg.

Chiquete [30], 2021; Mexico	Prospective study	Prehospital and in-hospital	ICH, IS, SAH<br><br>ICH: 107<br>IS: 209<br>SAH: 53	Uni- and multi-variable regression	ICH (median): 60<br>IS (median): 68<br>SAH (median): 62	≤6 h in most cases	CT or MRI scan	• AF<br>• DM<br>• HTN<br>• Obesity<br>• Previous stroke	• Focal motor deficit<br>• Headache<br>• Impaired LOC<br>• Language or speech disorder<br>• Vomiting	NR	• A combination of focal motor deficit and a history of HTN performed the best in predicting ICH patients among different stroke subtypes.
Gioia [31], 2016; Canada	Retrospective study	Prehospital and in-hospital	ICH, IS, TIA, SM<br><br>ICH: 51<br>IS: 367<br>TIA: 117<br>SM: 416	Uni-variable	ICH (mean): 73.1<br>IS (mean): 73.3<br>TIA (mean): 72.4<br>SM (mean): 66.9	Median time: 75 min	Brain imaging	• AF<br>• CAD<br>• CHF<br>• DM type 2<br>• Dyslipidaemia<br>• HTN<br>• Prior stroke, ICH, and TIA	NR	• SBP<br>• DBP<br>• MAP	• SM patients were younger than patients with IS/TIA/ICH.<br>• In comparison to the other groups, ICH patients had higher rates of previous ICH.<br>• ICH patients had a higher mean prehospital SBP (172.3 ± 31.7 mm Hg) compared to IS (154.9 ± 26.5 mm Hg), TIA (153.3 ± 23.1 mm Hg), and SM (145.4 ± 25.4 mm Hg).<br>• ICH patients also had a higher mean prehospital DBP (95.8 ± 22.1 mm Hg) compared to IS (87.7 ± 17.1 mm Hg), TIA (86.6 ± 15.9 mm Hg), and SM (84.7 ± 16.8 mm Hg).<br>• The mean prehospital MAP of the ICH group was higher (122.0 ± 20.2 mm Hg) compared to IS (110.2 ± 18.9 mm Hg), TIA (108.6 ± 16.6 mm Hg), and SM (105.1 ± 18.0 mm Hg).<br>• During EMS transport, the ICH group maintained the highest mean SBP.

Irisawa [32], 2013; Japan	Retrospective study	Prehospital and in-hospital	ICH, IS, SAH, non-stroke<br><br>ICH: 6699<br>IS: 24,754<br>SAH: 1631<br>Non-stroke: 73,622	Uni- and multi-variable regression	All patients (mean): 63.1	NR (Evaluated in prehospital)	NR	NR	• Impaired LOC	• SBP	• Among patients with stroke with impaired LOC, elevated SBP (≥200 mm Hg) in the prehospital setting was strongly associated with a final diagnosis of ICH.
Andersson Hagiwara [33], 2018; Sweden	Retrospective study	Prehospital and in-hospital	ICH, IS<br><br>ICH: 172<br>IS: 1164	Uni-variable	ICH (mean, median): 74, 78<br>IS (mean, median): 77, 78	<24 h from symptoms onset	CT scan	• AF<br>• Angina pectoris<br>• CHF<br>• DM<br>• HTN<br>• Intermittent claudication<br>• Malignancy<br>• Prior amaurosis fugax<br>• Prior MI<br>• Prior stroke and TIA<br>• Sleep apnoea	Prehospital assessment:<br>• Arm weakness<br>• Facial droop<br>• Impaired LOC<br>• Leg weakness<br>• Numbness<br>• Speech disturbances<br><br>In-hospital assessment:<br>• Aphasia/ dysphasia<br>• Compromised balance<br>• Double vision<br>• Dysphagia<br>• Facial droop<br>• Headache<br>• Hemiparesis/ plegia<br>• Impaired LOC<br>• Numbness<br>• Syncope<br>• Vertigo	• SBP<br>• DBP<br>• ECG<br>• HR<br>• SPO_2	• ICH patients were slightly younger than patients with IS.<br>• In terms of past medical history, a history of previous TIA was more common in the IS group.<br>• Patients with ICH woke up with symptoms less frequently than IS patients.<br>• Leg weakness and decreased LOC were more frequently observed in ICH patients in the prehospital setting.<br>• During the in-hospital assessment, patients with ICH tended to present more often with headaches, syncope, and decreased LOC.<br>• Both SBP >200 mm Hg, and DBP >90 mm Hg were more frequently observed in ICH patients in both prehospital and in-hospital settings.

Puustjärvi [34], 2015; Finland	Retrospective-prospective study	In-hospital	PC-ICH, PC-IS<br><br>PC-ICH: 363<br>PC-IS: 190	Uni- and multi-variable regression	ICH (median): 68<br>IS (median): 65	NR (Evaluated in prehospital)	CT or MRI scan	• AF<br>• Antiplatelet use<br>• CAD<br>• CHF<br>• DM<br>• Dyslipidaemia<br>• HTN<br>• Warfarin use	• Deterioration<br>• Headache<br>• Neurological deficits using NIHSS items<br>• Seizures<br>• Sudden onset<br>• Vomiting	• SBP<br>• DBP<br>• GCS<br>• BGL	• Patients with PC-ICH were older than those with PC-IS.<br>• PC-ICH patients were more likely to have a history of HTN and warfarin use, and less likely to have a history of AF or dyslipidaemia than PC-IS patients.<br>• Headache and vomiting at the onset were more frequently observed in the PC-ICH group.<br>• Upon arrival at the ER, PC-ICH patients had higher median BP (177/92 vs 153/86 mm Hg) as well as higher median BGL (7.4 vs 6.8 mmol/L) than PC-IS patients.
Bustamante [35], 2021; Spain	Prospective study	In-hospital	ICH, IS<br><br>ICH: 35<br>IS: 154	Uni- and multi-variable regression	ICH (median): 82<br>IS (median): 81	Within 4.5 h after symptoms onset	CT scan	• AF<br>• Alcohol intake<br>• CAD<br>• DM<br>• Dyslipidaemia<br>• HTN<br>• PAD<br>• Previous stroke<br>• Smoking	• Neurological deficits using NIHSS items	• SBP<br>• DBP<br>• BGL	• Patients with ICH tended to be more frequently male than IS patients.<br>• Patients with IS had higher rates of AF compared to ICH patients.<br>• ICH patients tended to present with a more severe stroke than patients with IS (median NIHSS 17 vs 13).<br>• The median admission BP of ICH patients was higher than that of IS patients (160/84 vs 149/77 mm Hg).
Montaner [36], 2012; Spain	Cross-sectional study	In-hospital	ICH, IS<br><br>ICH: 139<br>IS: 776	Uni- and multi-variable regression	ICH (mean): 70.35<br>IS (mean): 73.05	Within the first 24 h after symptoms onset	CT scan	• AF<br>• Alcohol intake<br>• CAD<br>• DM<br>• Dyslipidaemia<br>• Embolic cardiopathy<br>• HTN<br>• PAD<br>• Prior IS and ICH<br>• Smoking	• Neurological deficits using NIHSS items	NR	• Patients with ICH were younger and more likely to be male than IS patients.<br>• ICH patients had a higher history of ICH and alcohol use, but a lower history of dyslipidaemia, PAD, CAD, and AF, which were more prevalent among IS patients.<br>• Patients with ICH experienced a more severe stroke than those with IS (median NIHSS 11 vs 6).

Rozanski [37], 2017; Germany	Prospective study	Prehospital and in-hospital	ICH, IS<br><br>ICH: 25<br>IS: 49	Uni-variable	ICH (mean): 69.4<br>IS (mean): 75.3	Median time: 63 min	CT scanner in MSU	• AF<br>• DM<br>• Dyslipidaemia<br>• HTN<br>• Smoking	• Neurological deficits using NIHSS items	• SBP	• Patients with ICH were younger than those with IS.<br>• Hypercholesterolaemia (dyslipidaemia) was less common among ICH patients compared to IS patients.<br>• Patients with ICH presented with more severe strokes than IS patients (median NIHSS 15 vs 6).<br>• ICH patients had a higher mean SBP than IS patients (204 vs 162 mm Hg).
Bustamante [38], 2017; Spain	Prospective study	In-hospital	ICH, IS<br><br>ICH: 174<br>IS: 941	Uni- and multi-variable regression	ICH (median): 70<br>IS (median): 76	<6 h from symptoms onset	Brain imaging	• AF<br>• Alcohol intake<br>• CAD<br>• DM<br>• Dyslipidaemia<br>• HTN<br>• Previous stroke<br>• Smoking	• Neurological deficits using NIHSS items	• SBP<br>• DBP<br>• BGL	• ICH patients were younger and more often male than IS patients.<br>• Patients with ICH were more likely to have a history of HTN and less likely to have a history of AF or CAD than IS patients.<br>• ICH patients presented with more severe neurological deficits compared to patients with IS (median NIHSS 12 vs 7).<br>• Patients with ICH had higher median SBP (174 vs 155 mm Hg), DBP (92 vs 82 mm Hg), and BGL (7.1 vs 6.7 mmol/L*) than patients with IS.
Llombart [39], 2016; Spain	Retrospective study	In-hospital	ICH, IS<br><br>ICH: 28<br>IS: 38	Uni- and multi-variable regression	ICH (mean): 76.03<br>IS (mean): 72.28	Within 6 h from the onset of symptoms	CT scan	• AF<br>• CAD<br>• DM<br>• Dyslipidaemia<br>• HTN<br>• Previous stroke<br>• Smoking	• Neurological deficits using NIHSS items	NR	• A history of HTN and CAD was more common among IS patients than ICH patients.

*Converted from mg/dL to mmol/L by dividing by 18.

Abbreviations: ACS, acute coronary syndrome; AF, atrial fibrillation; BGL, blood glucose level; BP, blood pressure; CAD, coronary artery disease; CHF, congestive heart failure; CT, computed tomography; DBP, diastolic blood pressure; DC, derivation cohort; DM, diabetes mellitus; DOAC, direct oral anticoagulant; ECG, electrocardiogram; EMS, emergency medical services; ER, emergency room; GCS, Glasgow Coma Scale; HR, heart rate; HTN, hypertension; ICH, intracerebral haemorrhage; IS, ischaemic stroke; LOC, level of consciousness; LVO, large vessel occlusion; MAP, mean arterial pressure; MI, myocardial infarction; MRI, magnetic resonance imaging; MSU, mobile stroke unit; NIHSS, National Institutes of Health Stroke Scale; NR, not reported; PAD, peripheral artery disease; PC-ICH, posterior circulation intracerebral haemorrhage; PC-IS, posterior circulation ischaemic stroke; SAH, subarachnoid haemorrhage; SBP, systolic blood pressure; SM, stroke mimic; SPO_2, peripheral oxygen saturation; TIA, transient ischaemic attack; VC, validation cohort.

Supplementary Table 2. Characteristics of the included studies for portable technologies.

Included study, year and country	Design	Technology and device employed	Study population	Sample size (Number)	Time to results	Comparator	User expertise	Diagnostic accuracy					Potential limitations
								SEN (95% CI)	SPE (95% CI)	AUC (95% CI)	PPV (95% CI)	NPV (95% CI)	
Persson [40], 2014; Sweden	Proof-of-concept study	Microwave<br><br>2 prototype systems	Patients with stroke, healthy controls	Study 1: 20<br><br>ICH: 9<br><br>IS: 11<br><br>Study 2: 90<br><br>ICH: 10<br><br>IS: 15<br><br>Healthy controls: 65	NR	CT scan	Staff members from the engineering, neurophysiology, and nursing departments	The first clinical study (study 1): ICH vs IS					• The development of brain oedema after IS may limit the diagnostic accuracy of this imaging technique.
								NR	NR	0.88	NR	NR	
								The second clinical study (study 2): ICH vs IS					
								NR	NR	0.85	NR	NR	
								The second clinical study (study 2): ICH vs healthy controls					
								NR	NR	0.87	NR	NR	
Kellner [41], 2018; USA	Pilot study	VIPS<br><br>Cerebrotech Visor	Suspected stroke patients, healthy controls	248<br><br>Severe strokes: 57 (2 with ICH ≥60 ml)<br><br>Minor strokes: 26 (10 with ICH <60 ml)<br><br>Healthy controls: 79<br><br>Other subjects: 86	30 sec	Brain imaging and prehospital stroke scales	Trained staff	Severe strokes vs minor strokes					• Metallic implants (intracranial or extracranial) may interfere with the device's ability to provide accurate measurements.<br>• No evidence was provided that VIPS could distinguish ICH from other conditions (e.g., IS).
								93% (83–98)	92% (75–99)	0.93 (0.85–0.97)	96% (88–99)	86% (70–94)	
								Severe strokes vs all subjects enrolled					
								93% (83–98)	87% (81–92)	0.93 (0.89–0.96)	70% (61–77)	98% (94–99)	

Almubayyidh M, *et al. BMJ Open* 2024; 14:e079316. doi: 10.1136/bmjopen-2023-079316

Seidel [42], 1995; Germany	Prospective study	Ultrasound<br><br>HP SONOS 1000	Suspected stroke patients	84<br><br>ICH: 15<br><br>IS/TIA (in MCA): 48<br><br>IS/TIA (in PCA): 4<br><br>Other subjects: 17	NR	CT scan	NR	TCCS as a first diagnostic modality for ICH<br>88%	96%	NR	88%	96%	• The detection of small ICH (<1×1cm) is limited.<br>• Brain tumours like gliomas, and haemorrhagic infarcts could be misdiagnosed as a spontaneous ICH.<br>• A total of 20% (17/84) of the enrolled patients could not be assessed due to inadequate bone windows.
Mäurer [43], 1998; Germany	Prospective study	Ultrasound<br><br>Siemens Sonoline Ellegra or Acuson 128 XP/4	Suspected stroke patients	151<br><br>ICH: 60<br><br>IS: 67<br><br>Non-stroke: 24	NR	CT scan	Neuroradiologist	ICH vs IS<br>94%	95%	NR	91%	95%	• The assessment of the frontal and parasagittal brain regions is challenging when the acoustic bone window is narrow.<br>• Increased echo density of the white matter in patients with cerebral microangiopathy may mimic the sonographic pattern of ICH.

Almubayyidh M, *et al. BMJ Open* 2024; 14:e079316. doi: 10.1136/bmjopen-2023-079316

Antipova [44], 2020; UK	Exploratory study	Ultrasound<br><br>SonoSite M-Turbo, Philips Sparq, or Philips CX50 ultrasound	Suspected stroke patients	107<br><br>ICH: 18<br><br>IS: 63<br><br>TIA: 13<br><br>SM: 13	20 min	CT scan	Expert sonographer	ICH vs IS/TIA/SM based on TCCS and clinical examination					• Inadequate acoustic windows (18/107) and user dependency may hinder the potential application.
								63%	99%	0.912 (0.829–0.996)	91%	92%	
Michelson [45], 2015; USA	Retrospective study	EEG<br><br>BrainScope	Patients with stroke, SM controls	183<br><br>HS: 17<br><br>IS: 31<br><br>SM controls: 135	10 min	CT or MRI scan	Expert EEG technologist	Classification result of HS					• Previous TIAs/strokes and the use of neuroactive medications could alter EEG findings.<br>• The device was not demonstrated to be capable of distinguishing between stroke subtypes.
								94.1%	50.4% (43.0–58.7)	NR	NR	NR	
Erani [46], 2020; USA	Proof-of-concept study	EEG<br><br>Quick-20	Suspected stroke patients	100<br><br>ICH: 7<br><br>IS: 43<br><br>TIA: 13<br><br>Other diagnoses: 37	13 min	CT or MRI scan	Trained staff	Detection of stroke/TIA					• This device was not evaluated for its ability to distinguish ICH from other diagnoses.
								65%	80%	78.2	NR	NR	

Abbreviations: AUC, area under curve; CI, confidence interval; CT, computed tomography; EEG, electroencephalography; HS, haemorrhagic stroke; ICH, intracerebral haemorrhage; IS, ischaemic stroke; MCA, middle cerebral artery; MRI, magnetic resonance imaging; NPV, negative predictive value; NR, not reported; PCA, posterior cerebral artery; PPV, positive predictive value; SEN, sensitivity; SM, stroke mimic; SPE, specificity; TCCS, transcranial colour-coded duplex sonography; TIA, transient ischaemic attack; UK, United Kingdom; USA, United States of America; VIPS, volumetric impedance phase shift spectroscopy.
