# Supplementary material for: Prehospital identification of intracerebral haemorrhage: a scoping review of early clinical features and portable devices
Source: BMJ Open. 2024 Apr 19;14(4):e079316. doi: 10.1136/bmjopen-2023-079316 (PMC11033659; doi:10.1136/bmjopen-2023-079316)
Supplement: Supplementary data [file bmjopen-2023-079316supp003.pdf]

Appendix 2. Search strategies for the scoping review

1- The search strategy for clinical features

Database searched: MEDLINE (Ovid)

#	Query
1	exp Brain Ischemia/ or exp Stroke/ or exp Hemorrhagic Stroke/ or exp Cerebral Hemorrhage/ or exp Intracranial Hemorrhages/
2	Intracerebral hemorrhage.mp.
3	1 or 2
4	exp cerebrovascular accident/di
5	exp stroke/di
6	4 or 5
7	(recogni$ or identi$ or strati$ or different$ or probabilit$).mp.
8	3 and 7
9	6 or 8
10	exp Emergency Medical Services/ or Paramedic*.mp. or ambulance.mp. or triage.mp. or prehospital.mp. or pre hospital.mp. or EMS.mp. or emergency medical service*.mp.
11	Air Ambulances/
12	Emergency Service, Hospital/
13	Emergency Medicine/
14	Clinical feature.mp.
15	Stroke Score.mp.
16	Clinical differentiation.mp.
17	10 or 11 or 12 or 13 or 14 or 15 or 16
18	9 and 17
19	limit 18 to english language

Database searched: EMBASE (Ovid)

#	Query
1	exp Brain Ischemia/ or exp Stroke/ or exp Hemorrhagic Stroke/ or exp Cerebral Hemorrhage/ or exp Intracranial Hemorrhages/
2	Intracerebral hemorrhage.mp.
3	1 or 2
4	exp cerebrovascular accident/di [Diagnosis]
5	exp stroke/di
6	4 or 5
7	(recogni$ or identi$ or strati$ or distinguish$ or probabilit$).mp.
8	3 and 7
9	6 or 8
10	exp Emergency Medical Services/ or Paramedic*.mp. or ambulance.mp. or triage.mp. or prehospital.mp. or pre hospital.mp. or EMS.mp. or emergency medical service*.mp. or out-of-hospital.mp.
11	Air Ambulances/
12	Emergency Service, Hospital/
13	Stroke Score.mp.
14	Clinical differentiation.mp.
15	10 or 11 or 12 or 13 or 14
16	9 and 15
17	limit 16 to (human and english language)

Database searched: CENTRAL (Ovid)

#	Query
1	exp Brain Ischemia/ or exp Stroke/ or exp Hemorrhagic Stroke/ or exp Cerebral Hemorrhage/ or exp Intracranial Hemorrhages/
2	Intracerebral hemorrhage.mp.
3	1 or 2
4	exp cerebrovascular accident/di
5	exp stroke/di
6	4 or 5
7	(recogni$ or identi$ or strati$ or different$ or probabilit$).mp.
8	3 and 7
9	6 or 8
10	exp Emergency Medical Services/ or Paramedic*.mp. or ambulance.mp. or triage.mp. or prehospital.mp. or pre hospital.mp. or EMS.mp. or emergency medical service*.mp.
11	Air Ambulances/
12	Emergency Service, Hospital/
13	Emergency Medicine/
14	Clinical feature.mp.
15	Stroke Score.mp.
16	Clinical differentiation.mp.
17	10 or 11 or 12 or 13 or 14 or 15 or 16
18	9 and 17
19	limit 18 to english language

2- The search strategy for portable devices

Database searched: MEDLINE (Ovid)

#	Query
1	exp Brain Ischemia/ or exp Stroke/ or exp Hemorrhagic Stroke/ or exp Cerebral Hemorrhage/ or exp Intracranial Hemorrhages/
2	Intracerebral hemorrhage.mp.
3	1 or 2
4	(recogni$ or identi$ or strati$ or diagnos$ or detect$).mp.
5	exp Emergency Medical Services/ or Paramedic*.mp. or ambulance.mp. or triage.mp. or prehospital.mp. or pre hospital.mp. or EMS.mp. or emergency medical service*.mp.
6	Air Ambulances/
7	Emergency Service, Hospital/
8	(technology or spectroscopy or ultrasound or diagnostic imaging or imaging or transcranial doppler or radiofrequency or microwave or electroencephalography or device).mp.
9	5 or 6 or 7
10	3 and 4 and 8 and 9
11	limit 10 to (english language and humans)

Database searched: EMBASE (Ovid)

#	Query
1	exp Brain Ischemia/ or exp Stroke/ or exp Hemorrhagic Stroke/ or exp Cerebral Hemorrhage/ or exp Intracranial Hemorrhages/
2	Intracerebral hemorrhage.mp.
3	1 or 2
4	(recogni$ or identi$ or strati$ or diagnos$ or detect$).mp.
5	exp Emergency Medical Services/ or Paramedic*.mp. or ambulance.mp. or triage.mp. or prehospital.mp. or pre hospital.mp. or EMS.mp. or emergency medical service*.mp.
6	Air Ambulances/
7	Emergency Service, Hospital/
8	(technology or spectroscopy or ultrasound or diagnostic imaging or imaging or transcranial doppler or radiofrequency or microwave or electroencephalography or device).mp.
9	5 or 6 or 7
10	3 and 4 and 8 and 9
11	limit 10 to (english language and humans)

Database searched: CENTRAL (Ovid)

#	Query
1	exp Brain Ischemia/ or exp Stroke/ or exp Hemorrhagic Stroke/ or exp Cerebral Hemorrhage/ or exp Intracranial Hemorrhages/
2	Intracerebral hemorrhage.mp.
3	1 or 2
4	(recogni$ or identi$ or strati$ or diagnos$ or detect$).mp.
5	exp Emergency Medical Services/ or Paramedic*.mp. or ambulance.mp. or triage.mp. or prehospital.mp. or pre hospital.mp. or EMS.mp. or emergency medical service*.mp.
6	Air Ambulances/
7	Emergency Service, Hospital/
8	(technology or spectroscopy or ultrasound or diagnostic imaging or imaging or transcranial doppler or radiofrequency or microwave or electroencephalography or device).mp.
9	5 or 6 or 7
10	3 and 4 and 8 and 9
11	limit 10 to (english language and humans)
