# Supplementary material for: Prehospital identification of intracerebral haemorrhage: a scoping review of early clinical features and portable devices
Source: BMJ Open. 2024 Apr 19;14(4):e079316. doi: 10.1136/bmjopen-2023-079316 (PMC11033659; doi:10.1136/bmjopen-2023-079316)
Supplement: Author's manuscript [file bmjopen-2023-079316.draft_revisions.pdf]

BMJ Open

BMJ Open is committed to open peer review. As part of this commitment we make the peer review history of every article we publish publicly available.

When an article is published we post the peer reviewers' comments and the authors' responses online. We also post the versions of the paper that were used during peer review. These are the versions that the peer review comments apply to.

The versions of the paper that follow are the versions that were submitted during the peer review process. They are not the versions of record or the final published versions. They should not be cited or distributed as the published version of this manuscript.

BMJ Open is an open access journal and the full, final, typeset and author-corrected version of record of the manuscript is available on our site with no access controls, subscription charges or pay-per-view fees (http://bmjopen.bmj.com).

If you have any questions on BMJ Open's open peer review process please email info.bmjopen@bmj.com

BMJ Open

Prehospital identification of intracerebral haemorrhage: a scoping review of early clinical features and portable devices

Journal:	*BMJ Open*
Manuscript ID	bmjopen-2023-079316
Article Type:	Original research
Date Submitted by the Author:	28-Aug-2023

Complete List of Authors:	Almubayyidh, Mohammed; The University of Manchester, Division of Cardiovascular Sciences; King Saud University, Department of Aviation and Marine<br>Alghamdi, Ibrahim; The University of Manchester, Division of Cardiovascular Sciences; King Khalid University, Department of Emergency Medical Services<br>Parry-Jones, Adrian; The University of Manchester, Division of Cardiovascular Sciences; Northern Care Alliance NHS Foundation Trust, Manchester Centre for Clinical Neurosciences<br>Jenkins, David; The University of Manchester, Division of Informatics, Imaging and Data Science

Keywords:	Stroke < NEUROLOGY, STROKE MEDICINE, ACCIDENT & EMERGENCY MEDICINE

SCHOLARONE™
Manuscripts

I, the Submitting Author has the right to grant and does grant on behalf of all authors of the Work (as defined in the below author licence), an exclusive licence and/or a non-exclusive licence for contributions from authors who are: i) UK Crown employees; ii) where BMJ has agreed a CC-BY licence shall apply, and/or iii) in accordance with the terms applicable for US Federal Government officers or employees acting as part of their official duties; on a worldwide, perpetual, irrevocable, royalty-free basis to BMJ Publishing Group Ltd ("BMJ") its licensees and where the relevant Journal is co-owned by BMJ to the co-owners of the Journal, to publish the Work in this journal and any other BMJ products and to exploit all rights, as set out in our licence.

The Submitting Author accepts and understands that any supply made under these terms is made by BMJ to the Submitting Author unless you are acting as an employee on behalf of your employer or a postgraduate student of an affiliated institution which is paying any applicable article publishing charge ("APC") for Open Access articles. Where the Submitting Author wishes to make the Work available on an Open Access basis (and intends to pay the relevant APC), the terms of reuse of such Open Access shall be governed by a Creative Commons licence – details of these licences and which Creative Commons licence will apply to this Work are set out in our licence referred to above.

Other than as permitted in any relevant BMJ Author's Self Archiving Policies, I confirm this Work has not been accepted for publication elsewhere, is not being considered for publication elsewhere and does not duplicate material already published. I confirm all authors consent to publication of this Work and authorise the granting of this licence.

For peer review only

Prehospital identification of intracerebral haemorrhage: a scoping review of early clinical features and portable devices

Mohammed Almubayyidh[1,2*], Ibrahim Alghamdi[1,3], Adrian Parry-Jones[1,4], David Jenkins[5]

[1]Division of Cardiovascular Sciences, The University of Manchester, Manchester, UK

[2]Department of Aviation and Marine, Prince Sultan Bin Abdulaziz College for Emergency Medical Services, King Saud University, Riyadh, Saudi Arabia

[3]Department of Emergency Medical Services, College of Applied Medical Sciences, Khamis Mushait Campus, King Khalid University, Abha, Saudi Arabia

[4]Manchester Centre for Clinical Neurosciences, Northern Care Alliance NHS Foundation Trust, Salford, UK

[5]Division of Informatics, Imaging and Data Science, The University of Manchester, Manchester, UK

*Corresponding author:

Mohammed Almubayyidh, mohammed.almubayyidh@postgrad.manchester.ac.uk, Division of Cardiovascular Sciences, The University of Manchester, Manchester, UK

Abstract word count: 293

Text word count: 4989

For peer review only - http://bmjopen.bmj.com/site/about/guidelines.xhtml

ABSTRACT

Introduction

Prehospital identification of intracerebral haemorrhage (ICH) in suspected stroke cases may enable the initiation of appropriate treatments and facilitate better-informed transport decisions. This scoping review aims to examine the literature to identify early clinical features and portable devices for the detection of ICH in the prehospital setting. Additionally, the review aims to evaluate the feasibility of conducting a future meta-analysis.

Methods

Three databases were searched via Ovid (MEDLINE, EMBASE, and CENTRAL) from inception to August 2022 using pre-specified search strategies. One reviewer screened all titles, abstracts, and full-text articles for eligibility, while a second reviewer independently screened 20% of the literature during each screening stage. Data extracted were tabulated to summarise the key findings.

Results

A total of 6,803 articles were screened for eligibility, of which 25 studies were included for analysis. Among them, 15 studies reported on early clinical features, while 10 considered portable devices. Associations between age, sex, and comorbidities with the presence of ICH varied across studies. However, most studies reported that patients with ICH exhibited more severe neurological deficits (n=6) and higher blood pressure levels (n=11) at onset compared to other stroke and non-stroke diagnoses. Five technologies were identified for ICH detection: microwave imaging technology, volumetric impedance phase-shift spectroscopy, transcranial ultrasound, near-infrared spectroscopy, and electroencephalography. Microwave and ultrasound imaging techniques showed promise in distinguishing ICH from other diagnoses.

For peer review only - http://bmjopen.bmj.com/site/about/guidelines.xhtml

Conclusion

This scoping review has identified potential clinical features for the identification of ICH in suspected stroke patients. However, the considerable heterogeneity among the included studies precludes meta-analysis of available data. Moreover, we have explored portable devices to enhance ICH identification. While these devices have shown promise in detecting ICH, further testing is required to assess their ability to differentiate between stroke subtypes (ICH vs ischaemic stroke) and non-stroke diagnoses.

Keywords: Prehospital; Stroke; Intracerebral haemorrhage; Clinical features; Technology; Differential diagnosis.

STRENGTHS AND LIMITATIONS OF THIS STUDY

- To the best of our knowledge, this is the first review focused on identifying intracerebral haemorrhage (ICH) in the prehospital setting.
- No time of publication restrictions were applied to comprehensively map the available evidence.
- The review is limited to English language publications only.
- Consistent with the scoping review methodology, a formal risk of bias assessment was not conducted.

For peer review only

INTRODUCTION

Prehospital recognition of intracerebral haemorrhage (ICH) among suspected stroke cases is not part of standard care, but the ability to reliably do so may pave the way for much quicker delivery of time-critical treatments and triage to a hospital that can provide appropriate interventions (e.g., hyperacute stroke care, neurosurgery) [1,2]. The importance of the rapid identification of ICH patients has been highlighted by studies showing that early haematoma expansion (HE) frequently occurs within 2–3 hours of onset [2-4]. Thus, lowering blood pressure (BP) as well as administering anticoagulant reversal/haemostatic agents may limit HE, thereby increasing the likelihood of favourable outcomes [5-8].

While the initial diagnosis of stroke in the prehospital setting is based on stroke recognition tools (e.g., FAST—Face Arm Speech Test, CPSS—Cincinnati Prehospital Stroke Scale), these tools have been validated to identify all strokes rather than ICH specifically [9]. However, a number of clinical features were found to be predictive of ICH in stroke patients who experience persistent symptoms for more than 24 hours [10]. These features were used to develop clinical prediction rules to distinguish ICH from ischaemic stroke (IS) [10,11]. Nevertheless, the diagnostic accuracy of these rules is low, and they may not be applicable to patients presenting within a few hours of the onset of symptoms or during the prehospital phase [12,13]. Such tools for the identification of ICH may have the potential to improve patient outcomes. However, it is unclear which clinical features could be used to identify ICH in prehospital settings. Consequently, brain imaging (computed tomography [CT] and/or magnetic resonance imaging [MRI]) is the only accepted method for diagnosing ICH and other subtypes of stroke. This has led to the development of new portable imaging devices for the identification of stroke patients in prehospital settings [14,15].

The aim of this study is to perform a scoping review to summarise the early distinguishing clinical features of ICH and evaluate novel portable devices that can help prehospital personnel differentiate ICH from other causes of suspected stroke. Furthermore, this study aims to assess the feasibility of conducting a future meta-analysis to evaluate the results further quantitatively.

For peer review only - http://bmjopen.bmj.com/site/about/guidelines.xhtml

METHODS

The scoping review followed the guidelines of the Joanna Briggs Institute (JBI) [16,17]. It was reported according to the Preferred Reporting Items for Systematic Reviews and Meta-Analyses Extension for Scoping Reviews (PRISMA-ScR) checklist (Appendix 1) [18]. We adopted a scoping review approach because of a broad set of inclusion criteria. A detailed study protocol was published previously [19].

Eligibility criteria

The eligibility criteria for this review were classified using the 'Population, Concept, Context' framework suggested by the JBI (Table 1) [16]. The framework is applied as follows:

- Population: Adult patients (aged ≥16 years) with suspected stroke or intracranial haemorrhage confirmed by either CT or MRI scans. Studies were excluded if they enrolled children (aged <16 years) or combined the clinical features of spontaneous ICH patients with those of other conditions (e.g., subarachnoid haemorrhage [SAH]) in the same group without reporting them separately.
- Concept: First, to distinguish spontaneous ICH from other stroke and non-stroke diagnoses by investigating the early clinical features observed during the prehospital phase or within the initial 24 hours after the onset of symptoms upon arrival at the hospital. Second, to explore the use of portable devices in the detection and classification of ICH. Studies were excluded if they reported clinical features of ICH after 24 hours of symptoms onset, or if they investigated clinical features associated with ICH without comparing them against other suspected stroke cases. Further studies excluded from the review included those that did not provide information on the diagnostic accuracy of the tested technology, examined advances in conventional imaging techniques (CT and MRI), or exclusively tested the technology on phantoms or animal models.

For peer review only - http://bmjopen.bmj.com/site/about/guidelines.xhtml

- Context: Prehospital and in-hospital settings with no restrictions on the country of study, ethnicity, gender, or socioeconomic status. Studies conducted in primary care settings were excluded.

Sources of information and search strategy

We included data from primary research studies published in English. Conference abstracts, commentaries, surveys, case reports, preprints, animal studies, and non-English-language papers were excluded. The literature search of MEDLINE (via Ovid), EMBASE (via Ovid), and CENTRAL (via Ovid) databases was conducted in August 2022, using our pre-specified search strategies (Appendix 2) [19]. To ensure that all relevant studies were included, there was no restriction regarding the date of publication. In addition, we searched the reference lists of retrieved papers to identify additional relevant studies for this review.

Study selection

To eliminate duplicates and facilitate screening and collaboration, the identified studies were imported into EndNote and Rayyan Qatar Computing Research Institute (QCRI) software [20]. Two independent reviewers (MA and IA) selected eligible studies in two stages; initially, titles and abstracts were screened, and then full texts were assessed. MA screened all titles, abstracts, and full-text articles against the eligibility criteria. IA screened a random sample of 20% of the studies at both the title and abstract and full-text stages. Any disagreements in any phase of the selection process were resolved by discussion between reviewers to reach consensus or by a third reviewer (APJ or DAJ).

Data extraction

Two data extraction forms (Tables 2 and 3) were created using Microsoft Excel, considering the review objectives, the content of the included studies, and insights from previous reviews [12,21]. These tables were designed to present the data from the different studies in a standard format, allowing comparisons to be made between studies and their findings. The data were extracted by the first reviewer (MA), and the

For peer review only - http://bmjopen.bmj.com/site/about/guidelines.xhtml

second reviewer (IA) independently checked 20% of the extracted data to ensure accuracy.

Data synthesis and quality assessment of studies

Data were summarised as median, mean, and range when appropriate. Otherwise, a narrative approach was used to synthesise the findings of the included studies. Furthermore, since there is no expectation for a bias assessment on the PRISMA-ScR checklist [18], a formal risk of bias assessment was not conducted.

Patient and public involvement

No patients or public were involved in this study.

RESULTS

The search strategies yielded a total of 10,490 articles; after removing duplicates, 6,803 articles remained (Figure 1). Following the initial screening of titles and abstracts, 182 articles remained as potentially relevant and were retrieved for full-text review. After obtaining and reviewing the full texts, 159 studies were excluded with reasons, as illustrated in a PRISMA flowchart in Figure 1. The manual search of the reference lists of included studies revealed two additional studies, resulting in a total of 25 included studies.

Description of included studies

The retrieved studies were divided into two groups based on the aims of the scoping review: studies that reported the early clinical features of ICH patients (n=15) [22-36] and those that used portable devices to detect ICH (n=10) [37-46]. These groups were divided further according to the data extracted from them.

In the first group (Table 2), studies compared the clinical features among various populations, including stroke subtypes (n=12) and non-stroke diagnoses (n=3). Different statistical analyses were used in these studies to determine clinical features, such as univariable (n=3) and multivariable (n=12) regression analyses. Regardless of the statistical techniques used, the reported clinical features of interest were arranged

For peer review only - http://bmjopen.bmj.com/site/about/guidelines.xhtml

into three groups: patients' general information and medical history, early signs and symptoms, and vital signs. Studies that employed portable devices (Table 3) to detect or differentiate ICH and other diagnoses were divided into five sub-groups based on the underpinning technology: microwave imaging technology (n=1), volumetric impedance phase shift spectroscopy (VIPS) (n=1), transcranial ultrasound (n=3), near-infrared spectroscopy (NIRS) (n=3), and electroencephalography (EEG) (n=2). Additionally, most studies were conducted in Europe (n=15), followed equally by Asia (n=5) and North America (n=5).

Prediction of ICH in suspected stroke patients using early clinical features

Patients' general information and medical history

Patients with ICH were found to be younger (Table 2) [22-24,30,33-35], and more likely to be male [26,32,33,35] than those with other stroke and non-stroke diagnoses. However, this association was not uniform, as other studies found that neither age [25,26,28,32,36], nor sex [22-25,28,30,31,36], were predictors of the final diagnosis of ICH. Only one study identified an association between older age and increasing probability of posterior fossa ICH [31].

Several conditions or factors were identified as being more strongly associated with the presence of ICH (vs other diagnoses), including a history of hypertension (HTN) [27,28,31,35], prior ICH [23,28,33], haemodialysis [23], alcohol consumption [33] and the use of warfarin [31]. Conversely, patients with ICH were less likely to have a history of atrial fibrillation (AF) [23,24,26,31-33,35], coronary artery disease (CAD) [22,23,28,33,35,36], dyslipidaemia [23,28,31,33,34], diabetes mellitus (DM) [22,24], peripheral arteriopathy (PAD) [33], prior transient ischaemic attack (TIA) [28,30] or IS [25]. However, other studies found no or weak associations between ICH and HTN [23,24,26,30,32-34], prior TIA/IS [23,33], history of ICH [25], AF [22,30,34,36], CAD [30-32], dyslipidaemia [22,32,35,36], DM [23,28,30-36], PAD [32], alcohol consumption [32,35], or warfarin use [25]. In two studies [22,36], a history of HTN was found to be more common among IS patients than ICH.

For peer review only - http://bmjopen.bmj.com/site/about/guidelines.xhtml

For peer review only

Early signs and symptoms of ICH

Several symptoms were observed at the onset of ICH. Headache [23,30,31] and vomiting [23,24,31] occurred more frequently after ICH; however, three studies [22,25,27] did not find these symptoms to be specific to ICH. Additionally, five studies reported that impaired level of consciousness (LOC) was more prevalent among patients with ICH [22,23,26,29,30]. Meanwhile, two studies [25,27] found decreased LOC was not only associated with ICH, but also with large vessel occlusion (LVO) and SAH.

Seizures [23] and neurological deficits [23,26], including sensory or motor deficits [23,26], difficulty in following commands [23,26] and impaired vision [26] were also common presentations. Yet six studies [22,25-27,30,31] did not find the presence of seizures [22,26,31], or neurological deficits [22,25,27,30] to be specific discriminators of ICH. In terms of neurological deficits, one study found that compared to IS patients, ICH patients less frequently woke up with symptoms and they experienced greater leg weakness [30].

Eight studies [23,26,31-36] used the National Institutes of Health Stroke Scale (NIHSS) to assess stroke severity either in the prehospital setting [26] or at hospital admission [23,31-36]. Among these studies, six reported higher NIHSS scores at presentation in patients with ICH compared to those with IS, with median scores ranging from 11 to 17 for ICH patients and 6 to 13 for IS patients [23,26,32-35]. However, two studies found no discernible difference in NIHSS scores between ICH and IS patients [31,36].

Vital signs and their predictive capabilities of ICH

Most of the studies investigated vital signs, including systolic and diastolic BP (SBP and DBP, respectively) [22-26,28-32,34,35], mean arterial pressure (MAP) [26,28], heart rate (HR) or rhythm [22-25,30], peripheral oxygen saturation (SPO_2) [30], and blood glucose level [31,32,35].

In four studies that investigated the prehospital BP [24-26,30], the BP values were dichotomised as follows: SBP, >200 mm Hg [30], ≥180 mm Hg [24,26], ≥165 mm Hg [25]; DBP, ≥110 mm Hg [26], ≥100 mm Hg [24], ≥95 mm Hg [25], >90 mm Hg [30]; MAP,

For peer review only - http://bmjopen.bmj.com/site/about/guidelines.xhtml

≥130 mm Hg [26]. These studies found that a higher proportion of ICH patients had elevated BP at presentation than those with other stroke subtypes and non-stroke conditions.

Six studies reported that mean or median SBP [23,28,31,32,34,35], DBP [23,28,31,32,35], and MAP [28] values of ICH patients were higher at the time of onset than in patients with other diagnoses (SBP ranged from 160–204 mm Hg, DBP ranged from 84–102 mm Hg and the MAP was 122.0 ± 20.2 mm Hg). Only one study [22] found no differences in the SBP and DBP values of ICH and IS patients in the prehospital setting. However, another study found that elevated SBP (≥200 mm Hg) in the prehospital setting among stroke patients with impaired LOC was strongly associated with an increased risk of ICH [29].

With the exception of five studies, no differences were detected in vital signs such as HR [24,30] or rhythm [22,30], SPO_2 [30] and blood glucose [32] between ICH and IS patients. Three of the five studies reported that arrhythmia was less likely to be associated with ICH [23-25], and two observed higher median blood glucose levels at the time of ICH onset (7.1–7.4 mmol/L) compared to IS (6.7–6.8 mmol/L) [31,35].

Portable devices to detect and differentiate ICH

Microwave imaging technology

Two pilot studies were conducted to assess the diagnostic accuracy of microwave-based prototype devices in distinguishing ICH from IS, as well as ICH from healthy individuals [37]. In the first study, 20 stroke patients were examined using the first prototype device by engineering and neurophysiology staff. The device correctly differentiated 7 out of 11 IS patients from the ICH group, with an area under curve (AUC) of 0.88.

The second pilot study involved 25 stroke patients and 65 healthy individuals, with nursing staff testing the second custom-built helmet. The device accurately distinguished 14 out of 15 IS patients from the ICH group, achieving an AUC of 0.85 for distinguishing ICH from IS and 0.87 for distinguishing ICH patients from healthy individuals.

For peer review only - http://bmjopen.bmj.com/site/about/guidelines.xhtml

Volumetric impedance phase shift spectroscopy (VIPS)

The effectiveness of the VIPS visor device, developed by Cerebrotech, was investigated in a study aiming to assess its capability to measure the bioimpedance of each brain hemisphere for determining stroke severity [38]. The study enrolled 248 participants, including 41 'code stroke' patients, 79 healthy controls, and 128 patients with various neurological conditions at a comprehensive stroke centre. Trained personnel conducted three scans using the visor device, with a total scan duration of approximately 30 seconds.

The study aimed to evaluate the device's performance in distinguishing severe strokes from minor strokes and severe strokes from all other subjects. Severe strokes were defined as those requiring triage to a comprehensive stroke centre, including conditions such as LVO, NIHSS score of ≥6, ICH of ≥60 ml, and large territorial strokes.

The results of the study demonstrated that patients with severe strokes exhibited the highest mean bioimpedance asymmetry. The VIPS visor device achieved a sensitivity of 93% (95% confidence interval [CI]: 83–98%), specificity of 92% (95% CI: 75–99%), and an AUC of 0.93 (95% CI: 0.85–0.97) when differentiating severe strokes from minor strokes. Similarly, in distinguishing severe strokes from all tested subjects, the device showed a sensitivity of 93% (95% CI: 83–98%), a specificity of 87% (95% CI: 81–92%), and an AUC of 0.93 (95% CI: 0.89–0.96). The ability of VIPS to differentiate ICH from other conditions was not reported in this study.

Transcranial ultrasound

Three studies utilised transcranial ultrasound imaging to diagnose ICH and distinguish it from other suspected stroke causes [39-41]. In one clinical study [39], transcranial colour-coded duplex sonography (TCCS) scans were conducted on 84 patients using a Sonos 1000 Hewlett-Packard machine. ICH diagnosis was based on sharply demarcated hyperechogenic areas in the brain tissue. Among the patients, 52 had IS or TIA, 15 had ICH, and 17 were excluded due to inadequate temporal bone windows. TCCS identified ICH in 14 patients with focal hyperechogenicity, with only one small haemorrhage missed. Sensitivity and specificity were reported as 88% and 96%, respectively.

For peer review only - http://bmjopen.bmj.com/site/about/guidelines.xhtml

In another study [40], the diagnostic accuracy of TCCS was evaluated using Siemens Sonoline Ellegra or Acuson 128 XP/4 on 151 patients with acute hemiparesis. Among them, 60 had ICH, 67 had IS, and 24 had inconclusive CT scans. Ultrasound examination of 18 patients revealed insufficient acoustic bone windows. For the remaining 133 patients, TCCS detected 50 cases of ICH with a sensitivity of 94% and specificity of 95%.

A third study examined 107 suspected stroke patients using SonoSite M-Turbo, Philips Sparq, or Philips CX50 for TCCS [41]. Among the patients, 63 had acute IS, 18 had ICH, 13 had TIA, and 13 had stroke-mimicking conditions. Sonographic examination revealed an insufficient temporal bone window in 18 cases. Ultrasound imaging accurately identified ICH in 56% of patients, but failed in 33% of cases. The study presented a triage model that combined transcranial ultrasound and clinical assessment, resulting in a 10% improvement in ICH detection compared to clinical assessment alone. While the specificity of both techniques was reported as 99%, the sensitivity of the combined model was 63%, whereas clinical assessment alone yielded a sensitivity of 6%.

Near-infrared spectroscopy (NIRS)

Three studies investigated the accuracy of NIRS in detecting brain haematomas [42-44]. In a pilot study, the handheld NIRS Infrascanner was evaluated for its ability to detect intracranial haematomas in 35 patients with traumatic brain injuries (TBI) [42]. When compared to CT scans, which were considered the gold standard, the Infrascanner demonstrated a sensitivity of 88.89% (95% CI: 50.7–99.4%) and specificity of 81.25% (95% CI: 53.7–95.0%) in identifying intraparenchymal haematomas.

Two studies investigated the newer Infrascanner Model 2000 device in patients with TBI [43,44]. The device showed sensitivities ranging from 75% (95% CI: 64.63–83.62%) [43] to 100% (95% CI: 71.90–100%) [44] and specificities between 44.40% (95% CI: 35.80–44.40%) [44] and 50.43% (95% CI: 41.03–59.80%) [43]. When specific criteria for haematomas (volume >3.5 ml, distance from brain surface <2.5 cm) were considered, the Infrascanner's sensitivity increased from 75% to 89.36% [43]. It is

For peer review only - http://bmjopen.bmj.com/site/about/guidelines.xhtml

For peer review only

worth noting that the majority of enrolled patients in both studies had extracranial haemorrhages [43,44].

Electroencephalography (EEG)

Two studies examined the diagnostic capability of portable EEG devices for detecting strokes in emergency departments [45,46]. In one study [45], BrainScope's handheld device was used to identify strokes in 183 patients presenting with stroke-like symptoms. Among them, 31 patients were diagnosed with IS, 17 with haemorrhagic stroke, and 135 with stroke-mimicking conditions. The device recorded EEG data for 10 minutes while the patients kept their eyes closed, and the analysis was conducted using the Structural Brain Injury Index (SBII) algorithm. The SBII showed a sensitivity of 94.1% for detecting haemorrhagic strokes, with a specificity of 50.4% for stroke mimics serving as controls.

Another study assessed the Quick-20 EEG system for diagnosing acute stroke patients [46]. Out of the 100 patients involved, 63 were diagnosed with acute stroke/TIA, among whom 7 patients had ICH. EEG signals alone achieved a sensitivity of 65% and specificity of 80%, with an AUC of 78.2 for detecting acute stroke/TIA. Combining EEG with clinical data using deep learning improved diagnostic performance, yielding a sensitivity of 79%, a specificity of 80%, and an AUC of 87.8.

DISCUSSION

Overall, we identified fifteen studies that reported early clinical features of ICH among suspected stroke patients and ten studies that tested portable technologies to detect ICH and other conditions.

Early clinical features of ICH

Diagnosing ICH based solely on clinical features can be challenging, as many features of ICH overlap with other types of strokes [47]. This scoping review has identified potential clinical features that can help distinguish ICH patients from those with other stroke types or non-stroke diagnoses. The combination of these features might be useful in improving the early prediction of ICH. However, it is important to note that

For peer review only - http://bmjopen.bmj.com/site/about/guidelines.xhtml

the included studies investigated and reported different clinical features for ICH patients across various stroke subtypes, and some studies also included TIA and stroke-mimicking conditions in their analyses. In addition, some studies only conducted univariable analysis, and caution is advised when interpreting our results as there could be bias in the studies, such as confounding. Heterogeneity in the definition and categorisation of variables was also observed. Thus, limiting the ability to perform a meta-analysis of individual features.

In terms of patients' general information and medical history, age and sex did not consistently predict the final diagnosis of ICH. However, certain factors were found to be more strongly associated with the presence of ICH, including a history of HTN, prior ICH, haemodialysis, alcohol consumption, and the use of warfarin. Conversely, patients with ICH were less likely to have a history of AF, CAD, dyslipidaemia, DM, PAD, prior TIA, or IS. If available in the prehospital setting, electrocardiogram monitoring can be used to assess the presence of AF, which may help differentiate ICH from IS [23,26,48,49]. Nevertheless, the associations between these factors and ICH varied across studies. These results contradict a previous meta-analysis [12] that found a greater likelihood of ICH in male patients aged 60 years or less, while a history of TIA and PAD increased the probability of IS being diagnosed.

The initial signs and symptoms of ICH, such as headache, vomiting, and decreased LOC, also demonstrated variability across the included studies. While these manifestations were frequently observed, some studies did not find them to be exclusively indicative of ICH. This discrepancy could potentially be attributed to the inclusion of patients with SAH in certain studies [25,27]. SAH patients typically present with a sudden onset of headache, vomiting, decreased LOC, and neck stiffness [50,51]. Consequently, relying solely on these clinical features for the prediction of ICH may lack specificity when compared to SAH patients.

The majority of studies in this review consistently reported that patients with ICH experienced more severe neurological deficits at onset compared to other subtypes of stroke, TIA, and stroke-mimicking conditions. Furthermore, ICH patients were less commonly observed to have neurological deficits upon awakening when compared to patients with IS. These findings are in line with other studies that have reported a

For peer review only - http://bmjopen.bmj.com/site/about/guidelines.xhtml

higher frequency of hemiplegia/hemiparesis and sensory deficits in patients with cerebral haemorrhage [48,50], while stroke symptoms upon awakening were more frequently observed in patients with IS [50,52].

To assess and quantify neurological deficits, more than half of the studies utilised the NIHSS, which consistently showed higher scores in ICH patients. In fact, a recent study investigating the relationship between early NIHSS scores and ICH volume found that for every 10 ml increase in ICH volume, the NIHSS score increased by 4.5 points [53]. However, despite this, the NIHSS is not routinely used by prehospital personnel for stroke assessment due to its complexity and time-consuming nature [54]. In an effort to address these limitations, a recent study demonstrated that the NIHSS can be an accurate and time-efficient tool for paramedics in the field [55], suggesting its potential for improving the identification of ICH cases in the prehospital setting.

Several prediction tools have been proposed to differentiate ICH from other suspected stroke patients in the prehospital setting [22,24-27]. These tools can assist prehospital personnel in making accurate and timely decisions for stroke patients. However, their predictive abilities have not been thoroughly studied, compared, and evaluated in an independent study. Therefore, further research is necessary to assess their performance, reliability, and potential limitations or biases that may impact their effectiveness in real-world settings. The addition of the clinical features found in this review could further improve the performance of these tools.

Another important finding of this study is that patients with ICH had the highest BP parameters among all suspected stroke patients. Notably, there is a strong correlation between elevated SBP of ≥160 mm Hg and the initial haematoma volume in ICH patients [56]. A post-hoc analysis of the "FAST-MAG" trial revealed a distinct association between SBP and ultra-early neurological deterioration (defined as a deterioration of 2 or more points on the Glasgow Coma Scale within 2 hours of stroke onset) among ICH patients [57]. According to the study, neurological deterioration in patients with ICH was associated with higher SBP upon arrival at the ED. Additionally, imaging performed upon arrival showed that ICH patients with neurological deterioration had much larger haematoma volumes, suggesting that early HE may contribute to the deterioration [57]. Previous studies have also found an association

For peer review only - http://bmjopen.bmj.com/site/about/guidelines.xhtml

between prehospital BP values (SBP and DBP) and the occurrence of neurological deterioration in ICH [58,59]. These findings partially align with our results, in which there was a very powerful relationship between increased prehospital SBP and the occurrence of ICH among stroke patients with impaired LOC [29].

Other vital signs, such as HR and SPO_2, were compared and seemed to be insignificant in predicting the presence or absence of ICH. However, associations appear evident between cardiac arrhythmias in patients with IS or LVO, while higher blood glucose levels were observed in ICH patients upon hospital admission.

ICH detection technologies

Numerous portable technologies have been designed to identify and distinguish ICH from other stroke subtypes or stroke-mimicking diagnoses and to detect ICH in TBI patients. In addition to clinical features, this scoping review has identified portable devices that have the potential to improve the detection of ICH and could be used in various settings, including prehospital care.

In recent years, significant progress has been made in the prehospital diagnosis and treatment of ICH patients with the introduction of mobile stroke units (MSUs) [2,60,61]. However, due to the high costs associated with this technology, it is unlikely to become widely available for stroke care in the near future [62]. Thus, portable devices may serve as cost-effective alternatives to MSUs in the prehospital setting.

Some of the technologies evaluated showed greater accuracy than clinical assessments [38,41,46]. Antipova et al. [41] developed a triage model that combined transcranial sonographic findings with clinical assessment, resulting in significantly improved diagnostic sensitivity for detecting ICH. Hence, the combination of clinical features with the findings of these systems might further enhance the diagnostic accuracy of ICH.

The reviewed studies also highlighted the potential benefits of using these technologies for rapid diagnosis in the prehospital setting. For instance, in the VITAL study [38], the VIPS technology demonstrated an average detection time of 30 seconds for severe strokes. Other technologies evaluated had median application

For peer review only - http://bmjopen.bmj.com/site/about/guidelines.xhtml

For peer review only

times ranging from 3 to 20 minutes (Table 3), depending on the specific technology employed. Shortening the time-to-treatment duration is crucial in stroke management to improve patient outcomes [63]. Therefore, it is important to ensure that the use of diagnostic technologies does not cause delays in transporting patients to definitive care.

A number of limitations were identified for each technology. Most notably, the discriminatory ability to differentiate ICH from other diseases was not evaluated with VIPS and portable EEGs [38,45,46]. Additionally, while NIRS showed high sensitivity in detecting intracranial lesions, its effectiveness in accurately distinguishing between stroke subtypes, particularly in identifying deep ICH, may be hindered by its limited detection range of approximately 3 cm beneath the skull [42,43]. Conversely, microwave and ultrasound imaging techniques yielded good results in differentiating between stroke subtypes [37,39-41]. The diagnostic accuracy of microwave technology was not affected by the location of the stroke, but its ability to discriminate ICH from IS could be decreased with the development of cerebral oedema after IS [37]. On the other hand, transcranial ultrasound cannot produce useful images unless adequate temporal bone windows are available [39-41]. Furthermore, with this technology, pathological conditions such as haemorrhagic transformation of infarcts and gliomas could be misdiagnosed as ICH [39,40].

While several diagnostic modalities discussed in this review show promise for prehospital stroke care, their diagnostic accuracy requires further improvement and validation using a large, diverse cohort of suspected stroke patients. Such validation is crucial to establish their clinical usefulness in the prehospital setting, where both sensitivity and specificity are important in determining appropriate management. Given the significant differences in treatment strategies between ICH and IS, it is imperative that the detection technologies can effectively differentiate between the two with high sensitivity and specificity. Reliable differentiation can enable the prompt initiation of appropriate treatment measures, including BP reduction and anticoagulation reversal, and facilitate triaging patients to neurosurgical-capable facilities. Moreover, consideration should be given to the level of training required for personnel to competently operate these technologies [21]. For instance, devices such

For peer review only - http://bmjopen.bmj.com/site/about/guidelines.xhtml

as transcranial ultrasound necessitate highly trained operators, which may limit their feasibility in the prehospital setting.

Besides brain diagnostic devices, the use of blood biomarkers is also a promising approach for the early detection of stroke subtypes in the prehospital setting [34,64]. This is currently being investigated in a prehospital trial (Biomarkers for Initiating Onsite and Faster Ambulance Stroke Therapies, Bio-FAST, ClinicalTrials.gov Identifier: NCT04612218) with point-of-care test devices.

Limitations of the review

Our scoping review has some limitations that should be considered. Only one reviewer screened and analysed all of the data. Although a second reviewer independently screened 20% of the articles at the abstract, full-text, and data extraction stages, there is a risk of review errors and bias. Additionally, the search was limited to English publications, which could have resulted in the exclusion of important studies published in other languages. Lastly, owing to the nature of scoping reviews, the methodological quality of the studies included was not assessed [17]. This means that poor-quality studies were given equal weight as high-quality studies, which should be taken into account when interpreting the review findings.

CONCLUSION AND FUTURE WORK

Our study identifies potential early clinical features of ICH that distinguish it from suspected stroke patients. However, the substantial heterogeneity observed in populations, clinical features, and outcome variables among the included studies precludes the combination of these features in a meta-analysis. The variability in findings across studies highlights the need for further research and standardised tools to improve early prediction and differentiation of ICH. Future studies should focus on developing and validating predictive models or tools that incorporate easily obtainable clinical features for prehospital personnel. Additionally, it is important to consider an unselected cohort of suspected stroke patients in future studies to ensure that the findings are more widely applicable and representative of real-world practice.

For peer review only - http://bmjopen.bmj.com/site/about/guidelines.xhtml

Furthermore, large independent cohorts should be used to evaluate the performance, reliability, and potential limitations of these predictive tools.

In addition, this study presents portable technologies for the detection of ICH and other stroke subtypes. While some of these technologies have shown promising results, most are still in the early stages of development and will need to be tested in large multicentre or out-of-hospital settings. Moreover, the selected studies in this review reported different study designs, populations, and diagnostic performance, making comparisons between technologies difficult. To better understand the potential benefits and feasibility of using portable devices for prehospital stroke care, future studies should standardise the metrics of diagnostic test accuracy, the training requirements for device use, result acquisition time, and the technology's ability to differentiate between stroke subtypes (ICH vs IS) and non-stroke diagnoses.

ACKNOWLEDGEMENTS

The authors would like to express their sincere gratitude to the peer reviewers of the study's protocol for their valuable input and suggestions.

FUNDING STATEMENT

This study is part of MA's PhD research, which is funded by King Saud University, Riyadh, Saudi Arabia, through the Saudi Arabian Cultural Bureau in the United Kingdom.

COMPETING INTERESTS

None declared.

AUTHOR CONTRIBUTIONS

All authors included in this paper fulfil the criteria of authorship. MA is the primary author of this work under the supervision of APJ and DAJ. DAJ conceptualised the idea for the review. MA and IA screened the studies and performed data extraction. MA conducted data analyses and wrote the manuscript with support from APJ and DAJ.

For peer review only - http://bmjopen.bmj.com/site/about/guidelines.xhtml

For peer review only

The manuscript was critically reviewed and approved by all authors for final submission.

ETHICS APPROVAL

This is a scoping review; therefore, ethical approval is not required.

DATA AVAILABILITY STATEMENT

All data relevant to this study are included in the article or uploaded as supplementary information.

For peer review only

Table 1. Inclusion and exclusion criteria.

PCC	Included	Excluded
Population	• Studies of adult patients (≥16 years old) with suspected stroke or intracranial haemorrhage.	• Studies of children (<16 years old).<br>• Studies combined ICH clinical features with other aetiologies.
Concept	• Studies reported the clinical features of suspected stroke patients, obtained during prehospital care or within 24 hours of symptoms onset upon hospital admission.<br>• Studies evaluated portable devices to detect ICH.	• Studies examined the clinical features of patients after 24 hours of symptoms onset or focused on ICH-related clinical features without comparisons to other suspected stroke cases.<br>• Studies that failed to provide information on the diagnostic performance of the technology being tested, or examined advances in conventional detection methods, or restricted their testing to phantoms or animal models.
Context	• Prehospital and in-hospital studies.	• Primary care studies.

Abbreviations: ICH, intracerebral haemorrhage; PCC, population, concept, context.

For peer review only - http://bmjopen.bmj.com/site/about/guidelines.xhtml

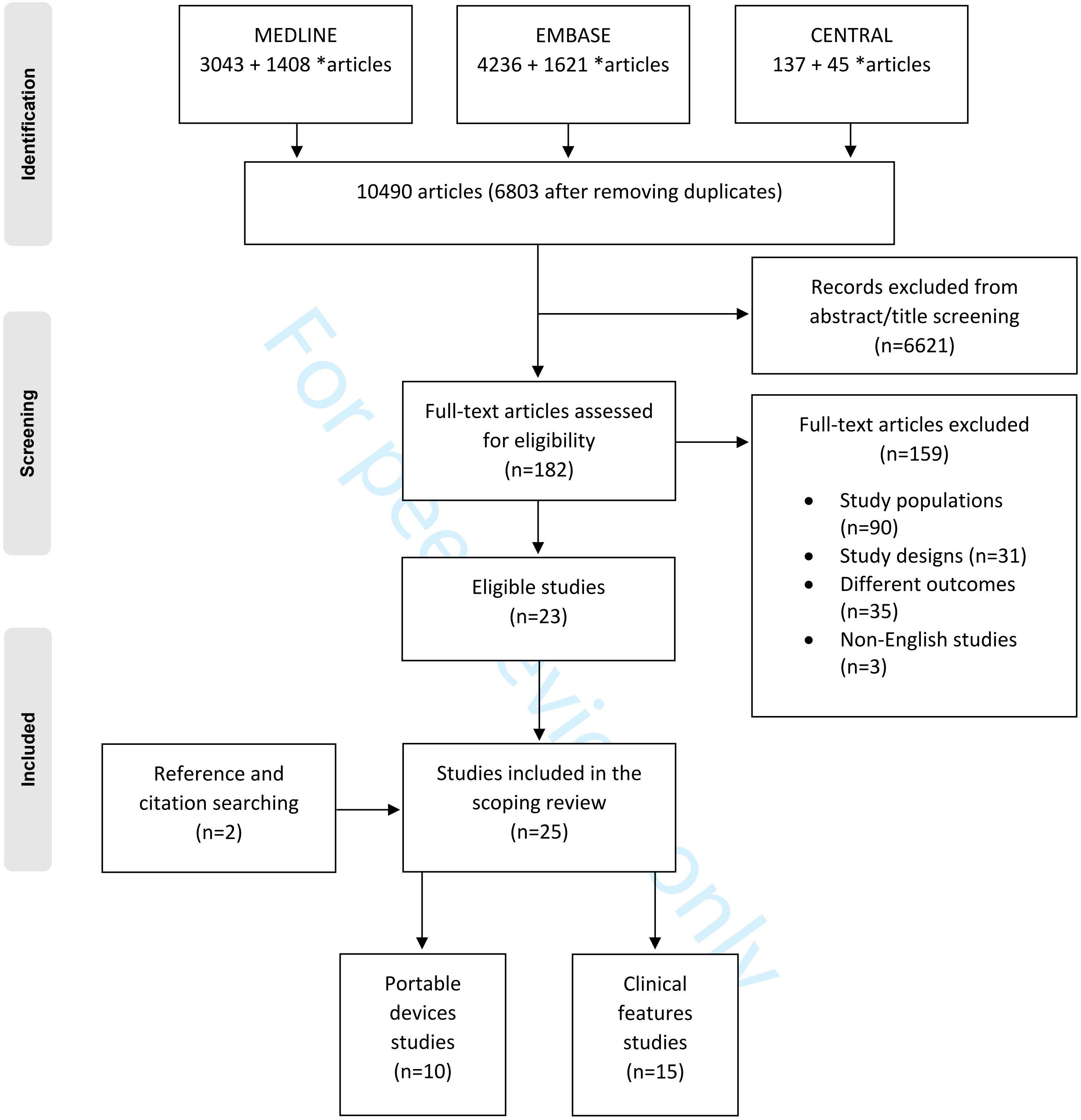

Figure 1. Flowchart of study selection process (*search results for clinical features + portable devices, respectively).

For peer review only - http://bmjopen.bmj.com/site/about/guidelines.xhtml

Table 2. Characteristics of the included studies for clinical features.

Included study, year and country	Design	Setting	Study population	Statistical method	Age (Years)	Onset-to-evaluation	Method of confirmation	Clinical features collected			Findings relevant to review
								Past medical history	Signs and symptoms	Vital signs	
Woisetschläger [22], 2000; Austria	Retrospective-prospective study	Prehospital and in-hospital	ICH, IS<br><br>ICH: 118<br>IS: 106	Uni- and multi-variable regression	ICH (mean): 58<br>IS (mean): 65	NR (Evaluated in prehospital)	CT scan	• AF<br>• Aspirin use<br>• Dicoumarin use<br>• DM<br>• HTN<br>• Hyperlipidaemia<br>• Prior MI<br>• Smoking	• Aphasia<br>• Headache<br>• Hemisymptoms<br>• Impaired LOC<br>• Seizures<br>• Syncope<br>• Vomiting	• SBP<br>• DBP<br>• ECG	• ICH patients were younger than IS patients.<br>• A history of HTN, DM, and MI was more commonly observed in IS patients.<br>• Impaired LOC was predictive of ICH.<br>• Unilateral weakness or sensory loss was observed more frequently in IS patients.
Yamashita [23], 2011; Japan	Retrospective study	Prehospital and in-hospital	ICH, IS<br><br>ICH: 100<br>IS: 127	Uni- and multi-variable regression	ICH (median): 68<br>IS (median): 74	Within 6 h of onset	CT or MRI scan	• ACS<br>• AF<br>• Antithrombotics use<br>• DM<br>• Haemodialysis<br>• HTN<br>• Hyperlipidaemia<br>• Prior TIA, IS, and ICH<br>• Smoking	• Headache<br>• Neurological deficits using NIHSS items<br>• Seizures<br>• Vomiting	• SBP<br>• DBP<br>• ECG	• ICH patients were younger than IS patients.<br>• Patients with ICH were more likely to have a history of ICH and haemodialysis, while patients with IS had a higher likelihood of a history of ACS, AF, and antithrombotic usage.<br>• Headache, vomiting, impaired LOC, and seizures were more frequently observed in the ICH group.<br>• The NIHSS median score was higher in the ICH group (16 vs 9).<br>• The median BP was higher in the ICH group, including both SBP (190 vs 164 mm Hg) and DBP (102 vs 90 mm Hg).
Jin [24], 2016; China	Prospective study	Prehospital and in-hospital	ICH, IS, SAH, non-stroke<br><br>ICH: 689<br>IS: 797<br>SAH: 109<br>Non-stroke: 394	Uni- and multi-variable regression	All patients (mean): 66	NR (Evaluated in prehospital)	CT or MRI scan	• AF<br>• DM<br>• HTN	• Vomiting	• SBP<br>• DBP<br>• ECG<br>• HR	• The comparison was between two groups: ICH vs IS/TIA.<br>• Age <65 years was found to be associated with the ICH group.<br>• IS/TIA patients were more likely to have a history of AF and DM.<br>• Vomiting was a common presenting symptom in the ICH group.<br>• SBP ≥180 mm Hg and DBP ≥100 mm Hg were more frequently observed in ICH patients, while arrhythmia was a common finding in the IS/TIA group.

For peer review only - http://bmjopen.bmj.com/site/about/guidelines.xhtml

For peer review only

Uchida [25], 2018; Japan	Prospective study	Prehospital and in-hospital	Any stroke, ICH, LVO, SAH, non-stroke<br><br>DC: 1229<br>Any stroke: 203<br>ICH: 169<br>LVO: 104<br>SAH: 57<br>Non-stroke: 696<br><br>VC: 1007<br>Any stroke: 253<br>ICH: 183<br>LVO: 131<br>SAH: 50<br>Non-stroke: 390	Uni- and multi-variable regression	DC (median): 72<br><br>VC (median): 75	NR (Evaluated in prehospital)	CT or MRI scan	• Antiplatelet use<br>• DOAC use<br>• Prior IS, ICH, and SAH<br>• Smoking<br>• Warfarin use	• Anisocoria<br>• Aphasia<br>• Conjugate deviation<br>• Convulsion<br>• Dizziness<br>• Dysarthria<br>• Facial palsy<br>• Headache<br>• Impaired LOC<br>• Nausea, or vomiting<br>• Numbness<br>• Paralysis of lower limbs<br>• Paralysis of upper limbs<br>• Sudden onset<br>• Symptoms improved after onset<br>• Symptoms progressed after onset<br>• Unilateral spatial neglect	• SBP<br>• DBP<br>• ECG	• Patients with ICH were less likely to have a history of IS.<br>• Improvement following the onset of symptoms was associated with a decreased probability of ICH.<br>• Headache, dysarthria, paralysis of the upper limbs, conjugate deviation, and impaired LOC were common presentations of ICH. However, most of these symptoms were not specific.<br>• SBP ≥165 mm Hg and DBP ≥95 mm Hg were more frequently observed in ICH patients, while the presence of arrhythmia was associated with the absence of ICH.
Geisler [26], 2021; Germany	Retrospective study	Prehospital and in-hospital	ICH, IS, TIA, SM<br><br>DC: 416<br>ICH: 32<br>IS: 224<br>TIA: 41<br>SM: 119<br><br>VC: 285<br>ICH: 33<br>IS: 252	Uni- and multi-variable regression	DC (mean):<br>ICH: 71.5<br>IS: 74.9<br><br>VC (mean):<br>ICH: 73.3<br>IS: 73.9	≤4 h or unknown	CT scanner in MSU	• AF<br>• HTN	• Neurological deficits using NIHSS items<br>• Seizures	• SBP<br>• DBP<br>• MAP	• In the derivation set (ICH vs IS), ICH patients were more likely to be male.<br>• AF was found less frequently in the derivation set for the ICH group.<br>• ICH patients had higher median NIHSS scores in both sets (≥15).<br>• A greater number of ICH patients showed a decrease in LOC.<br>• The majority of seizures were found in the SM group.<br>• A higher proportion of ICH patients exhibited SBP ≥180 mm Hg, DBP ≥110 mm Hg, and MAP ≥130 mm Hg.

For peer review only

For peer review only - http://bmjopen.bmj.com/site/about/guidelines.xhtml

Chiquete [27], 2021; Mexico	Prospective study	Prehospital and in-hospital	ICH, IS, SAH<br><br>ICH: 107<br>IS: 209<br>SAH: 53	Uni- and multi-variable regression	ICH (median): 60<br>IS (median): 68<br>SAH (median): 62	≤6 h in most cases	CT or MRI scan	• AF<br>• DM<br>• HTN<br>• Obesity<br>• Previous stroke	• Focal motor deficit<br>• Headache<br>• Impaired LOC<br>• Language or speech disorder<br>• Vomiting	NR	• A combination of focal motor deficit and a history of HTN performed the best in predicting ICH patients among different stroke subtypes.
Gioia [28], 2016; Canada	Retrospective study	Prehospital and in-hospital	ICH, IS, TIA, SM<br><br>ICH: 51<br>IS: 367<br>TIA: 117<br>SM: 416	Uni-variable	ICH (mean): 73.1<br>IS (mean): 73.3<br>TIA (mean): 72.4<br>SM (mean): 66.9	Median time: 75 min	Brain imaging	• AF<br>• CAD<br>• CHF<br>• DM type 2<br>• Dyslipidaemia<br>• HTN<br>• Prior stroke, ICH, and TIA	NR	• SBP<br>• DBP<br>• MAP	• SM patients were younger and had lower rates of HTN and AF than patients with IS/TIA/ICH.<br>• IS patients had higher rates of CAD and dyslipidaemia compared to other groups.<br>• In comparison to the other groups, ICH patients had higher rates of previous ICH, whereas TIA patients had higher rates of previous TIA.<br>• ICH patients had a higher mean prehospital SBP (172.3 ± 31.7 mm Hg) compared to IS (154.9 ± 26.5 mm Hg), TIA (153.3 ± 23.1 mm Hg), and SM (145.4 ± 25.4 mm Hg).<br>• ICH patients also had a higher mean prehospital DBP (95.8 ± 22.1 mm Hg) compared to IS (87.7 ± 17.1 mm Hg), TIA (86.6 ± 15.9 mm Hg), and SM (84.7 ± 16.8 mm Hg).<br>• The mean prehospital MAP of the ICH group was higher (122.0 ± 20.2 mm Hg) compared to IS (110.2 ± 18.9 mm Hg), TIA (108.6 ± 16.6 mm Hg), and SM (105.1 ± 18.0 mm Hg).<br>• During EMS transport, the ICH group maintained the highest mean SBP.

For peer review only

For peer review only - http://bmjopen.bmj.com/site/about/guidelines.xhtml

Irisawa [29], 2013; Japan	Retrospective study	Prehospital and in-hospital	ICH, IS, SAH, non-stroke<br><br>ICH: 6699<br>IS: 24,754<br>SAH: 1631<br>Non-stroke: 73,622	Uni- and multi-variable regression	All patients (mean): 63.1	NR (Evaluated in prehospital)	NR	NR	• Impaired LOC	• SBP	• Among stroke patients with impaired LOC, elevated SBP (≥200 mm Hg) in the prehospital setting was strongly associated with a final diagnosis of ICH.
Andersson Hagiwara [30], 2018; Sweden	Retrospective study	Prehospital and in-hospital	ICH, IS<br><br>ICH: 172<br>IS: 1164	Uni-variable	ICH (mean, median): 74, 78<br>IS (mean, median): 77, 78	<24 h from symptoms onset	CT scan	• AF<br>• Angina pectoris<br>• CHF<br>• DM<br>• HTN<br>• Intermittent claudication<br>• Malignancy<br>• Prior amaurosis fugax<br>• Prior MI<br>• Prior stroke, and TIA<br>• Sleep apnoea	Prehospital assessment:<br>• Arm weakness<br>• Facial droop<br>• Impaired LOC<br>• Leg weakness<br>• Numbness<br>• Speech disturbances<br><br>In-hospital assessment:<br>• Aphasia/ dysphasia<br>• Compromised balance<br>• Double vision<br>• Dysphagia<br>• Facial droop<br>• Headache<br>• Hemiparesis/ plegia<br>• Impaired LOC<br>• Numbness<br>• Syncope<br>• Vertigo	• SBP<br>• DBP<br>• ECG<br>• HR<br>• SPO_2	• ICH patients were slightly younger than patients with IS.<br>• In terms of past medical history, a history of previous TIA was more common in the IS group.<br>• Patients with ICH woke up with symptoms less frequently than IS patients.<br>• Leg weakness and decreased LOC were more frequently observed in ICH patients in the prehospital setting.<br>• During the in-hospital assessment, patients with ICH tended to present more often with headaches, syncope, and decreased LOC.<br>• Both SBP >200 mm Hg, and DBP >90 mm Hg were more frequently observed in ICH patients in both prehospital and in-hospital settings.

For peer review only

For peer review only - http://bmjopen.bmj.com/site/about/guidelines.xhtml

Puustjärvi [31], 2015; Finland	Retrospective -prospective study	In-hospital	PC-ICH, PC-IS<br><br>PC-ICH: 363<br>PC-IS: 190	Uni- and multi-variable regression	ICH (median): 68<br>IS (median): 65	NR (Evaluated in prehospital)	CT or MRI scan	• AF<br>• Antiplatelet use<br>• CAD<br>• CHF<br>• DM<br>• Dyslipidaemia<br>• HTN<br>• Warfarin use	• Headache<br>• Neurological deficits using NIHSS items<br>• Seizures<br>• Sudden onset<br>• Vomiting	• SBP<br>• DBP<br>• GCS<br>• BGL	• Patients with PC-ICH were older than those with PC-IS.<br>• PC-ICH patients were more likely to have a history of HTN and warfarin use, and less likely to have a history of AF or dyslipidaemia than PC-IS patients.<br>• Headache and vomiting at the onset were more frequently observed in the PC-ICH group.<br>• Upon arrival at the ER, PC-ICH patients had higher median BP (177/92 vs 153/86 mm Hg) as well as higher median BGL (7.4 vs 6.8 mmol/l) than PC-IS patients.
Bustamante [32], 2021; Spain	Prospective study	In-hospital	ICH, IS<br><br>ICH: 35<br>IS: 154	Uni- and multi-variable regression	ICH (median): 82<br>IS (median): 81	Within 4.5 h after symptoms onset	CT scan	• AF<br>• Alcohol intake<br>• CAD<br>• DM<br>• Dyslipidaemia<br>• HTN<br>• PAD<br>• Previous stroke<br>• Smoking	• Neurological deficits using NIHSS items	• SBP<br>• DBP<br>• BGL	• Patients with ICH tended to be more frequently male than IS patients.<br>• Patients with IS had higher rates of AF compared to ICH patients.<br>• ICH patients tended to present with a more severe stroke than patients with IS (median NIHSS 17 vs 13).<br>• The median admission BP of ICH patients was higher than that of IS patients (160/84 vs 149/77 mm Hg).
Montaner [33], 2012; Spain	Cross-sectional study	In-hospital	ICH, IS<br><br>ICH: 139<br>IS: 776	Uni- and multi-variable regression	ICH (mean): 70.35<br>IS (mean): 73.05	Within the first 24 h after symptoms onset	CT scan	• AF<br>• Alcohol intake<br>• CAD<br>• DM<br>• Dyslipidaemia<br>• Embolic cardiopathy<br>• HTN<br>• PAD<br>• Prior IS, and ICH<br>• Smoking	• Neurological deficits using NIHSS items	NR	• Patients with ICH were younger and more likely to be male than IS patients.<br>• ICH patients had a higher history of ICH and alcohol use, but a lower history of dyslipidaemia, PAD, CAD, and AF, which were more prevalent among IS patients.<br>• Patients with ICH experienced a more severe stroke than those with IS (median NIHSS 11 vs 6).

For peer review only - http://bmjopen.bmj.com/site/about/guidelines.xhtml

Rozanski [34], 2017; Germany	Prospective study	Prehospital and in-hospital	ICH, IS<br>ICH: 25<br>IS: 49	Uni-variable	ICH (mean): 69.4<br>IS (mean): 75.3	Median time: 63 min	CT scanner in MSU	• AF<br>• DM<br>• Dyslipidaemia<br>• HTN<br>• Smoking	• Neurological deficits using NIHSS items	• SBP	• Patients with ICH were younger than those with IS.<br>• Hypercholesterolaemia (dyslipidaemia) was less common among ICH patients compared to IS patients.<br>• Patients with ICH presented with more severe strokes than IS patients (median NIHSS 15 vs 6).<br>• ICH patients had a higher mean SBP than IS patients (204 vs 162 mm Hg).
Bustamante [35], 2017; Spain	Prospective study	In-hospital	ICH, IS<br>ICH: 174<br>IS: 941	Uni- and multi-variable regression	ICH (median): 70<br>IS (median): 76	<6 h from symptoms onset	Brain imaging	• AF<br>• Alcohol intake<br>• CAD<br>• DM<br>• Dyslipidaemia<br>• HTN<br>• Previous stroke<br>• Smoking	• Neurological deficits using NIHSS items	• SBP<br>• DBP<br>• BGL	• ICH patients were younger and more often male than IS patients.<br>• Patients with ICH were more likely to have a history of HTN and less likely to have a history of AF or CAD than IS patients.<br>• ICH patients presented with more severe neurological deficits compared to patients with IS (median NIHSS 12 vs 7).<br>• Patients with ICH had higher median SBP (174 vs 155 mm Hg), DBP (92 vs 82 mm Hg), and BGL (7.1 vs 6.7 mmol/L*) than patients with IS.
Llombart [36], 2016; Spain	Retrospective study	In-hospital	ICH, IS<br>ICH: 28<br>IS: 38	Uni- and multi-variable regression	ICH (mean): 76.03<br>IS (mean): 72.28	Within 6 h from the onset of symptoms	CT scan	• AF<br>• CAD<br>• DM<br>• Dyslipidaemia<br>• HTN<br>• Previous stroke<br>• Smoking	• Neurological deficits using NIHSS items	NR	• A history of HTN and CAD was more common among IS patients than ICH patients.

*Converted from mg/dL to mmol/L by dividing by 18.

Abbreviations: ACS, acute coronary syndrome; AF, atrial fibrillation; BGL, blood glucose level; BP, blood pressure; CAD, coronary artery disease; CHF, congestive heart failure; CT, computed tomography; DBP, diastolic blood pressure; DC, derivation cohort; DM, diabetes mellitus; DOAC, direct oral anticoagulant; ECG, electrocardiogram; EMS, emergency medical services; ER, emergency room; GCS, Glasgow Coma Scale; HR, heart rate; HTN, hypertension; ICH, intracerebral haemorrhage; IS, ischaemic stroke; LOC, level of consciousness; LVO, large vessel occlusion; MAP, mean arterial pressure; MI, myocardial infarction; MRI, magnetic resonance imaging; MSU, mobile stroke unit; NIHSS, National Institutes of Health Stroke Scale; NR, not reported; PAD, peripheral artery disease; PC-ICH, posterior circulation intracerebral haemorrhage; PC-IS, posterior circulation ischaemic stroke; SAH, subarachnoid haemorrhage; SBP, systolic blood pressure; SM, stroke mimic; SPO_2, peripheral oxygen saturation; TIA, transient ischaemic attack; VC, validation cohort.

For peer review only

For peer review only - http://bmjopen.bmj.com/site/about/guidelines.xhtml

Table 3. Characteristics of the included studies for portable technologies.

Included study, year and country	Design	Technology and device employed	Study population	Sample size (Number)	Time to results	Comparator	User expertise	Diagnostic accuracy					Potential limitations
								SEN (95% CI)	SPE (95% CI)	AUC (95% CI)	PPV (95% CI)	NPV (95% CI)	
Persson [37], 2014; Sweden	Proof-of-concept study	Microwave<br><br>2 prototype systems	Stroke patients, healthy controls	Study 1: 20<br><br>ICH: 9<br><br>IS: 11<br><br>Study 2: 90<br><br>ICH: 10<br><br>IS: 15<br><br>Healthy controls: 65	NR	CT scan	Staff members from the engineering, neurophysiology, and nursing departments	The first clinical study (study 1): ICH vs IS					• The development of brain oedema after IS may limit the diagnostic accuracy of this imaging technique.
								NR	NR	0.88	NR	NR	
								The second clinical study (study 2): ICH vs IS					
								NR	NR	0.85	NR	NR	
								The second clinical study (study 2): ICH vs healthy controls					
								NR	NR	0.87	NR	NR	
Kellner [38], 2018; USA	Pilot study	VIPS<br><br>Cerebrotech Visor	Suspected stroke patients, healthy controls	248<br><br>Severe strokes: 57 (2 with ICH ≥60 ml)<br><br>Minor strokes: 26 (10 with ICH <60 ml)<br><br>Healthy controls: 79<br><br>Other subjects: 86	30 sec	Brain imaging and prehospital stroke scales	Trained staff	Severe strokes vs minor strokes					• Metallic implants (intracranial or extracranial) may interfere with the device's ability to provide accurate measurements.<br>• No evidence was provided that VIPS could distinguish ICH from other conditions (e.g., IS).
								93% (83–98)	92% (75–99)	0.93 (0.85–0.97)	96% (88–99)	86% (70–94)	
								Severe strokes vs all subjects enrolled					
								93% (83–98)	87% (81–92)	0.93 (0.89–0.96)	70% (61–77)	98% (94–99)	

For peer review only - http://bmjopen.bmj.com/site/about/guidelines.xhtml

Seidel [39], 1995; Germany	Prospective study	Ultrasound<br><br>HP SONOS 1000	Suspected stroke patients	84<br><br>ICH: 15<br><br>IS/TIA (in MCA): 48<br><br>IS/TIA (in PCA): 4<br><br>Other subjects: 17	NR	CT scan	NR	TCCS as a first diagnostic modality for ICH					• The detection of small ICH (<1×1cm) is limited.<br>• Brain tumours like gliomas, and haemorrhagic infarcts could be misdiagnosed as a spontaneous ICH.<br>• A total of 20% (17/84) of the enrolled patients could not be assessed due to inadequate bone windows.
								88%	96%	NR	88%	96%	
Mäurer [40], 1998; Germany	Prospective study	Ultrasound<br><br>Siemens Sonoline Ellegra or Acuson 128 XP/4	Suspected stroke patients	151<br><br>ICH: 60<br><br>IS: 67<br><br>Non-stroke: 24	NR	CT scan	Neuroradiologist	ICH vs IS					• The assessment of the frontal and parasagittal brain regions is challenging when the acoustic bone window is narrow.<br>• Increased echo density of the white matter in patients with cerebral microangiopathy may mimic the sonographic pattern of ICH.
								94%	95%	NR	91%	95%	

For peer review only - http://bmjopen.bmj.com/site/about/guidelines.xhtml

Antipova [41], 2020; UK	Exploratory study	Ultrasound<br><br>SonoSite M-Turbo, Philips Sparq, or Philips CX50 ultrasound	Suspected stroke patients	107<br><br>ICH: 18<br><br>IS: 63<br><br>TIA: 13<br><br>SM: 13	20 min	CT scan	Expert sonographer	ICH vs IS/TIA/SM based on TCCS and clinical examination					• Inadequate acoustic windows (18/107) and user dependency may hinder the potential application.
								63%	99%	0.912 (0.829–0.996)	91%	92%	
Leon-Carrion [42], 2010; Spain	Pilot study	NIRS<br><br>Infrascanner	TBI patients	35<br><br>Intracranial haemorrhage: 19<br><br>Negative CT: 16	3 min	CT scan	Trained staff	Intra-axial haematomas (intraparenchymal)					• The detection depth of the device is limited to approximately 3 cm below the skull.
								88.89% (50.7–99.4)	81.25% (53.7–95.0)	NR	72.72% (39.3–92.7)	92.85% (64.1–99.6)	
Kontojannis [43], 2019; UK	Pilot study	NIRS<br><br>Infrascanner 2000	TBI patients	205<br><br>CT positive: 87<br><br>CT negative: 118	3.74 min	CT scan	Trained staff	Any intracranial haematomas (including ICH)					• The results of Infrascanner should not be used for on-site treatment due to the high rate of false positives.<br>• Infrascanner cannot be used to guide management of small volume or deep intracranial bleeds (e.g., ICH).
								75% (64.63–83.62)	50.43% (41.03–59.80)	NR	53.23% (47.76–58.62)	72.84% (64.16–80.07)	
								Intracranial haematomas with volume >3.5 ml and depth <2.5 cm from the brain's surface					
								89.36% (76.90–96.45)	48.73% (40.71–56.80)	NR	34.15% (30.20–38.33)	93.90% (86.88–97.28)	
Yuksen [44], 2020; Thailand	Prospective study	NIRS<br><br>Infrascanner 2000	TBI patients	47<br><br>CT positive: 11<br><br>CT negative: 36	3 min	CT scan	Trained staff	Any intracranial haematomas (including ICH)					• Infrascanner cannot detect the size or location of bleeding.<br>• The majority of patients in this study suffered from extracranial haematomas (90.9%).
								100% (71.90–100)	44.40% (35.80–44.40)	0.722	35.50% (25.50–35.50)	100% (80.70–100)	

For peer review only

For peer review only - http://bmjopen.bmj.com/site/about/guidelines.xhtml

Michelson [45], 2015; USA	Retrospective study	EEG<br>BrainScope	Stroke patients, SM controls	183<br>HS: 17<br>IS: 31<br>SM controls: 135	10 min	CT or MRI scan	Expert EEG technologist	Classification result of HS					• Previous TIAs/strokes and the use of neuroactive medications could alter EEG findings.<br>• The device was not demonstrated to be capable of distinguishing between stroke subtypes.
								94.1%	50.4% (43.0–58.7)	NR	NR	NR	
Erani [46], 2020; USA	Proof-of-concept study	EEG<br>Quick-20	Suspected stroke patients	100<br>ICH: 7<br>IS: 43<br>TIA: 13<br>Other diagnoses: 37	13 min	CT or MRI scan	Trained staff	Detection of stroke/TIA					• This device was not evaluated for its ability to distinguish ICH from other diagnoses.
								65%	80%	78.2	NR	NR	

Abbreviations: AUC, area under curve; CI, confidence interval; CT, computed tomography; EEG, electroencephalography; HS, haemorrhagic stroke; ICH, intracerebral haemorrhage; IS, ischaemic stroke; MCA, middle cerebral artery; MRI, magnetic resonance imaging; NIRS, near-infrared spectroscopy; NPV, negative predictive value; NR, not reported; PCA, posterior cerebral artery; PPV, positive predictive value; SEN, sensitivity; SM, stroke mimic; SPE, specificity; TBI, traumatic brain injury; TCCS, transcranial colour-coded duplex sonography; TIA, transient ischaemic attack; UK, United Kingdom; USA, United States of America; VIPS, volumetric impedance phase shift spectroscopy.

For peer review only

REFERENCES

1. Colton K, Richards CT, Pruitt PB, et al. Early Stroke Recognition and Time-based Emergency Care Performance Metrics for Intracerebral Hemorrhage. J Stroke Cerebrovasc Dis. 2020;29(2):104552.

2. Bowry R, Parker SA, Bratina P, et al. Hemorrhage Enlargement Is More Frequent in the First 2 Hours: A Prehospital Mobile Stroke Unit Study. Stroke. 2022;53(7):2352-60.

3. Brott T, Broderick J, Kothari R, et al. Early hemorrhage growth in patients with intracerebral hemorrhage. Stroke. 1997;28(1):1-5.

4. Broderick JP, Diringer MN, Hill MD, et al. Determinants of intracerebral hemorrhage growth: an exploratory analysis. Stroke. 2007;38(3):1072-5.

5. Rodriguez-Luna D, Rodriguez-Villatoro N, Juega JM, et al. Prehospital Systolic Blood Pressure Is Related to Intracerebral Hemorrhage Volume on Admission. Stroke. 2018;49(1):204-6.

6. Song L, Chen C, Chen X, et al. INTEnsive ambulance-delivered blood pressure Reduction in hyper-ACute stroke Trial (INTERACT4): study protocol for a randomized controlled trial. Trials. 2021;22(1):885.

7. Yassi N, Zhao H, Churilov L, et al. Tranexamic acid for intracerebral haemorrhage within 2 hours of onset: protocol of a phase II randomised placebo-controlled double-blind multicentre trial. Stroke Vasc Neurol. 2022;7(2):158-65.

8. Naidech AM, Grotta J, Elm J, et al. Recombinant factor VIIa for hemorrhagic stroke treatment at earliest possible time (FASTEST): Protocol for a phase III, double-blind, randomized, placebo-controlled trial. Int J Stroke. 2021:806-9.

9. Greenberg SM, Ziai WC, Cordonnier C, et al. 2022 Guideline for the Management of Patients With Spontaneous Intracerebral Hemorrhage: A Guideline From the American Heart Association/American Stroke Association. Stroke. 2022;53(7):e282-e361.

10. Sandercock PA, Allen CM, Corston RN, et al. Clinical diagnosis of intracranial haemorrhage using Guy's Hospital score. Br Med J (Clin Res Ed). 1985;291(6510):1675-7.

For peer review only - http://bmjopen.bmj.com/site/about/guidelines.xhtml

11. Poungvarin N, Viriyavejakul A, Komontri C. Siriraj stroke score and validation study to distinguish supratentorial intracerebral haemorrhage from infarction. BMJ. 1991;302(6792):1565-7.

12. Runchey S, McGee S. Does this patient have a hemorrhagic stroke? Clinical findings distinguishing hemorrhagic stroke from ischemic stroke. JAMA. 2010;303(22):2280-6.

13. Badam P, Solao V, Pai M, et al. Poor accuracy of the Siriraj and Guy's hospital stroke scores in distinguishing haemorrhagic from ischaemic stroke in a rural, tertiary care hospital. Natl Med J India. 2003;16(1):8-12.

14. Walsh KB. Non-invasive sensor technology for prehospital stroke diagnosis: Current status and future directions. Int J Stroke. 2019;14(6):592-602.

15. Lin MP, Sanossian N, Liebeskind DS. Imaging of prehospital stroke therapeutics. Expert Rev Cardiovasc Ther. 2015;13(9):1001-15.

16. Peters M, Godfrey C, Khalil H, et al. 2017 Guidance for the Conduct of JBI Scoping Reviews. 2017.

17. Peters MDJ, Marnie C, Colquhoun H, et al. Scoping reviews: reinforcing and advancing the methodology and application. Syst Rev. 2021;10(1):263.

18. Tricco AC, Lillie E, Zarin W, et al. PRISMA Extension for Scoping Reviews (PRISMA-ScR): Checklist and Explanation. Ann Intern Med. 2018;169(7):467-73.

19. Almubayyidh M, Alghamdi I, Parry-Jones AR, et al. Clinical features and novel technologies for prehospital detection of intracerebral haemorrhage: a scoping review protocol. BMJ Open. 2023;13(5):e070228.

20. Ouzzani M, Hammady H, Fedorowicz Z, et al. Rayyan-a web and mobile app for systematic reviews. Syst Rev. 2016;5(1):210.

21. Chennareddy S, Kalagara R, Smith C, et al. Portable stroke detection devices: a systematic scoping review of prehospital applications. BMC Emerg Med. 2022;22(1):111.

22. Woisetschläger C, Kittler H, Oschatz E, et al. Out-of-hospital diagnosis of cerebral infarction versus intracranial hemorrhage. Intensive Care Med. 2000;26(10):1561-5.

23. Yamashita S, Kimura K, Iguchi Y, et al. Kurashiki Prehospital Stroke Subtyping Score (KP3S) as a means of distinguishing ischemic from hemorrhagic stroke in emergency medical services. Eur Neurol. 2011;65(4):233-8.

For peer review only - http://bmjopen.bmj.com/site/about/guidelines.xhtml

24. Jin HQ, Wang JC, Sun YA, et al. Prehospital Identification of Stroke Subtypes in Chinese Rural Areas. Chin Med J (Engl). 2016;129(9):1041-6.

25. Uchida K, Yoshimura S, Hiyama N, et al. Clinical Prediction Rules to Classify Types of Stroke at Prehospital Stage. Stroke. 2018;49(8):1820-7.

26. Geisler F, Wesirow M, Ebinger M, et al. Probability assessment of intracerebral hemorrhage in prehospital emergency patients. Neurol Res Pract. 2021;3(1):1.

27. Chiquete E, Jiménez-Ruiz A, García-Grimshaw M, et al. Prediction of acute neurovascular syndromes with prehospital clinical features witnessed by bystanders. Neurol Sci. 2021;42(8):3217-24.

28. Gioia LC, Zewude RT, Kate MP, et al. Prehospital systolic blood pressure is higher in acute stroke compared with stroke mimics. Neurology. 2016;86(23):2146-53.

29. Irisawa T, Iwami T, Kitamura T, et al. An association between systolic blood pressure and stroke among patients with impaired consciousness in out-of-hospital emergency settings. BMC Emerg Med. 2013;13:24.

30. Andersson Hagiwara M, Wireklint Sundström B, Brink P, et al. A shorter system delay for haemorrhagic stroke than ischaemic stroke among patients who use emergency medical service. Acta Neurol Scand. 2018;137(5):523-30.

31. Puustjärvi V, Strbian D, Tiainen M, et al. Recognition of posterior circulation stroke. Acta Neurol Scand. 2015;131(6):389-93.

32. Bustamante A, Penalba A, Orset C, et al. Blood Biomarkers to Differentiate Ischemic and Hemorrhagic Strokes. Neurology. 2021;96(15):e1928-e39.

33. Montaner J, Mendioroz M, Delgado P, et al. Differentiating ischemic from hemorrhagic stroke using plasma biomarkers: the S100B/RAGE pathway. J Proteomics. 2012;75(15):4758-65.

34. Rozanski M, Waldschmidt C, Kunz A, et al. Glial Fibrillary Acidic Protein for Prehospital Diagnosis of Intracerebral Hemorrhage. Cerebrovasc Dis. 2017;43(1-2):76-81.

35. Bustamante A, López-Cancio E, Pich S, et al. Blood Biomarkers for the Early Diagnosis of Stroke: The Stroke-Chip Study. Stroke. 2017;48(9):2419-25.

36. Llombart V, García-Berrocoso T, Bustamante A, et al. Plasmatic retinol-binding protein 4 and glial fibrillary acidic protein as biomarkers to differentiate ischemic stroke and intracerebral hemorrhage. J Neurochem. 2016;136(2):416-24.

For peer review only - http://bmjopen.bmj.com/site/about/guidelines.xhtml

37. Persson M, Fhager A, Trefná HD, et al. Microwave-based stroke diagnosis making global prehospital thrombolytic treatment possible. IEEE Trans Biomed Eng. 2014;61(11):2806-17.
38. Kellner CP, Sauvageau E, Snyder KV, et al. The VITAL study and overall pooled analysis with the VIPS non-invasive stroke detection device. J Neurointerv Surg. 2018;10(11):1079-84.
39. Seidel G, Kaps M, Gerriets T. Potential and limitations of transcranial color-coded sonography in stroke patients. Stroke. 1995;26(11):2061-6.
40. Mäurer M, Shambal S, Berg D, et al. Differentiation between intracerebral hemorrhage and ischemic stroke by transcranial color-coded duplex-sonography. Stroke. 1998;29(12):2563-7.
41. Antipova D, Eadie L, Makin S, et al. The use of transcranial ultrasound and clinical assessment to diagnose ischaemic stroke due to large vessel occlusion in remote and rural areas. PLoS One. 2020;15(10):e0239653.
42. Leon-Carrion J, Dominguez-Roldan JM, Leon-Dominguez U, et al. The Infrascanner, a handheld device for screening in situ for the presence of brain haematomas. Brain Inj. 2010;24(10):1193-201.
43. Kontojannis V, Hostettler I, Brogan RJ, et al. Detection of intracranial hematomas in the emergency department using near infrared spectroscopy. Brain Inj. 2019;33(7):875-83.
44. Yuksen C, Sricharoen P, Puengsamran N, et al. Diagnostic properties of a portable near-infrared spectroscopy to detect intracranial hematoma in traumatic brain injury patients. Eur J Radiol Open. 2020;7:100246.
45. Michelson EA, Hanley D, Chabot R, et al. Identification of acute stroke using quantified brain electrical activity. Acad Emerg Med. 2015;22(1):67-72.
46. Erani F, Zolotova N, Vanderschelden B, et al. Electroencephalography Might Improve Diagnosis of Acute Stroke and Large Vessel Occlusion. Stroke. 2020;51(11):3361-5.
47. Broderick J, Connolly S, Feldmann E, et al. Guidelines for the Management of Spontaneous Intracerebral Hemorrhage in Adults. Stroke. 2007;38(6):2001-23.
48. Besson G, Robert C, Hommel M, et al. Is It Clinically Possible to Distinguish Nonhemorrhagic Infarct From Hemorrhagic Stroke? Stroke. 1995;26(7):1205-9.

For peer review only - http://bmjopen.bmj.com/site/about/guidelines.xhtml

49. Efstathiou SP, Tsioulos DI, Zacharos ID, et al. A new classification tool for clinical differentiation between haemorrhagic and ischaemic stroke. J Intern Med. 2002;252(2):121-9.
50. Massaro AR, Sacco RL, Scaff M, et al. Clinical discriminators between acute brain hemorrhage and infarction: a practical score for early patient identification. Arq Neuropsiquiatr. 2002;60(2-a):185-91.
51. Edlow JA, Caplan LR. Avoiding pitfalls in the diagnosis of subarachnoid hemorrhage. N Engl J Med. 2000;342(1):29-36.
52. Lee SE, Choi MH, Kang HJ, et al. Stepwise stroke recognition through clinical information, vital signs, and initial labs (CIVIL): Electronic health record-based observational cohort study. PLoS One. 2020;15(4):e0231113.
53. Farooq S, Shkirkova K, Villablanca P, et al. National Institutes of Health Stroke Scale Correlates Well with Initial Intracerebral Hemorrhage Volume. J Stroke Cerebrovasc Dis. 2022;31(4):106348.
54. Larsen K, Hov MR, Sandset EC. Prehospital stroke scales-the need for a gold standard in the field. Acta Neurol Scand. 2022;145(3):263-4.
55. Larsen K, Jæger HS, Hov MR, et al. Streamlining Acute Stroke Care by Introducing National Institutes of Health Stroke Scale in the Emergency Medical Services: A Prospective Cohort Study. Stroke. 2022;53(6):2050-7.
56. Ohwaki K, Yano E, Nagashima H, et al. Blood pressure management in acute intracerebral hemorrhage: relationship between elevated blood pressure and hematoma enlargement. Stroke. 2004;35(6):1364-7.
57. Shkirkova K, Saver JL, Starkman S, et al. Frequency, Predictors, and Outcomes of Prehospital and Early Postarrival Neurological Deterioration in Acute Stroke: Exploratory Analysis of the FAST-MAG Randomized Clinical Trial. JAMA Neurol. 2018;75(11):1364-74.
58. Tsou YJ, Lan KP, Fan JS. Relationship Between Changes in Prehospital Blood Pressure and Early Neurological Deterioration in Spontaneous Intracerebral Hemorrhage. Adv Emerg Nurs J. 2019;41(2):163-71.
59. Moon JS, Janjua N, Ahmed S, et al. Prehospital neurologic deterioration in patients with intracerebral hemorrhage. Crit Care Med. 2008;36(1):172-5.

For peer review only - http://bmjopen.bmj.com/site/about/guidelines.xhtml

60. Gomes JA, Ahrens CL, Hussain MS, et al. Prehospital reversal of warfarin-related coagulopathy in intracerebral hemorrhage in a mobile stroke treatment unit. Stroke. 2015;46(5):e118-20.

61. Cooley SR, Zhao H, Campbell BCV, et al. Mobile Stroke Units Facilitate Prehospital Management of Intracerebral Hemorrhage. Stroke. 2021;52(10):3163-6.

62. Walter S, Grunwald IQ, Helwig SA, et al. Mobile Stroke Units - Cost-Effective or Just an Expensive Hype? Curr Atheroscler Rep. 2018;20(10):49.

63. Pulvers JN, Watson JDG. If Time Is Brain Where Is the Improvement in Prehospital Time after Stroke? Front Neurol. 2017;8:617.

64. Mattila OS, Ashton NJ, Blennow K, et al. Ultra-Early Differential Diagnosis of Acute Cerebral Ischemia and Hemorrhagic Stroke by Measuring the Prehospital Release Rate of GFAP. Clin Chem. 2021;67(10):1361-72.

For peer review only

For peer review only - http://bmjopen.bmj.com/site/about/guidelines.xhtml

Appendix 1. Preferred Reporting Items for Systematic reviews and Meta-Analyses extension for Scoping Reviews (PRISMA-ScR) Checklist

SECTION	ITEM	PRISMA-ScR CHECKLIST ITEM	REPORTED ON PAGE #
**TITLE**			
Title	1	Identify the report as a scoping review.	1
**ABSTRACT**			
Structured summary	2	Provide a structured summary that includes (as applicable): background, objectives, eligibility criteria, sources of evidence, charting methods, results, and conclusions that relate to the review questions and objectives.	2–3
**INTRODUCTION**			
Rationale	3	Describe the rationale for the review in the context of what is already known. Explain why the review questions/objectives lend themselves to a scoping review approach.	4
Objectives	4	Provide an explicit statement of the questions and objectives being addressed with reference to their key elements (e.g., population or participants, concepts, and context) or other relevant key elements used to conceptualize the review questions and/or objectives.	4–5
**METHODS**			
Protocol and registration	5	Indicate whether a review protocol exists; state if and where it can be accessed (e.g., a Web address); and if available, provide registration information, including the registration number.	5
Eligibility criteria	6	Specify characteristics of the sources of evidence used as eligibility criteria (e.g., years considered, language, and publication status), and provide a rationale.	6
Information sources*	7	Describe all information sources in the search (e.g., databases with dates of coverage and contact with authors to identify additional sources), as well as the date the most recent search was executed.	6
Search	8	Present the full electronic search strategy for at least 1 database, including any limits used, such that it could be repeated.	Appendix 2
Selection of sources of evidence†	9	State the process for selecting sources of evidence (i.e., screening and eligibility) included in the scoping review.	6
Data charting process‡	10	Describe the methods of charting data from the included sources of evidence (e.g., calibrated forms or forms that have been tested by the team before their use, and whether data charting was done independently or in duplicate) and any processes for obtaining and confirming data from investigators.	6
Data items	11	List and define all variables for which data were sought and any assumptions and simplifications made.	Appendix 2
Critical appraisal of individual sources of evidence§	12	If done, provide a rationale for conducting a critical appraisal of included sources of evidence; describe the methods used and how this information was used in any data synthesis (if appropriate).	N/A
Synthesis of results	13	Describe the methods of handling and summarizing the data that were charted.	6-7

St.Michael's
Inspired Care.
Inspiring Science.

For peer review only - http://bmjopen.bmj.com/site/about/guidelines.xhtml

SECTION	ITEM	PRISMA-ScR CHECKLIST ITEM	REPORTED ON PAGE #
**RESULTS**			
Selection of sources of evidence	14	Give numbers of sources of evidence screened, assessed for eligibility, and included in the review, with reasons for exclusions at each stage, ideally using a flow diagram.	7,21
Characteristics of sources of evidence	15	For each source of evidence, present characteristics for which data were charted and provide the citations.	7
Critical appraisal within sources of evidence	16	If done, present data on critical appraisal of included sources of evidence (see item 12).	N/A
Results of individual sources of evidence	17	For each included source of evidence, present the relevant data that were charted that relate to the review questions and objectives.	22–31
Synthesis of results	18	Summarize and/or present the charting results as they relate to the review questions and objectives.	7–13
**DISCUSSION**			
Summary of evidence	19	Summarize the main results (including an overview of concepts, themes, and types of evidence available), link to the review questions and objectives, and consider the relevance to key groups.	13–17
Limitations	20	Discuss the limitations of the scoping review process.	17–18
Conclusions	21	Provide a general interpretation of the results with respect to the review questions and objectives, as well as potential implications and/or next steps.	18
**FUNDING**			
Funding	22	Describe sources of funding for the included sources of evidence, as well as sources of funding for the scoping review. Describe the role of the funders of the scoping review.	19

JBI = Joanna Briggs Institute; PRISMA-ScR = Preferred Reporting Items for Systematic reviews and Meta-Analyses extension for Scoping Reviews.

* Where *sources of evidence* (see second footnote) are compiled from, such as bibliographic databases, social media platforms, and Web sites.

† A more inclusive/heterogeneous term used to account for the different types of evidence or data sources (e.g., quantitative and/or qualitative research, expert opinion, and policy documents) that may be eligible in a scoping review as opposed to only studies. This is not to be confused with *information sources* (see first footnote).

‡ The frameworks by Arksey and O'Malley (6) and Levac and colleagues (7) and the JBI guidance (4, 5) refer to the process of data extraction in a scoping review as data charting.

§ The process of systematically examining research evidence to assess its validity, results, and relevance before using it to inform a decision. This term is used for items 12 and 19 instead of "risk of bias" (which is more applicable to systematic reviews of interventions) to include and acknowledge the various sources of evidence that may be used in a scoping review (e.g., quantitative and/or qualitative research, expert opinion, and policy document).

From: Tricco AC, Lillie E, Zarin W, O'Brien KK, Colquhoun H, Levac D, et al. PRISMA Extension for Scoping Reviews (PRISMAScR): Checklist and Explanation. Ann Intern Med. 2018;169:467–473. doi: 10.7326/M18-0850.

For peer review only

For peer review only - http://bmjopen.bmj.com/site/about/guidelines.xhtml

Appendix 2. Search strategies for the scoping review

1- The search strategy for clinical features

Database searched: MEDLINE (Ovid)

#	Query
1	exp Brain Ischemia/ or exp Stroke/ or exp Hemorrhagic Stroke/ or exp Cerebral Hemorrhage/ or exp Intracranial Hemorrhages/
2	Intracerebral hemorrhage.mp.
3	1 or 2
4	exp cerebrovascular accident/di
5	exp stroke/di
6	4 or 5
7	(recogni$ or identi$ or strati$ or different$ or probabilit$).mp.
8	3 and 7
9	6 or 8
10	exp Emergency Medical Services/ or Paramedic*.mp. or ambulance.mp. or triage.mp. or prehospital.mp. or pre hospital.mp. or EMS.mp. or emergency medical service*.mp.
11	Air Ambulances/
12	Emergency Service, Hospital/
13	Emergency Medicine/
14	Clinical feature.mp.
15	Stroke Score.mp.
16	Clinical differentiation.mp.
17	10 or 11 or 12 or 13 or 14 or 15 or 16
18	9 and 17
19	limit 18 to english language

For peer review only

Database searched: EMBASE (Ovid)

#	Query
1	exp Brain Ischemia/ or exp Stroke/ or exp Hemorrhagic Stroke/ or exp Cerebral Hemorrhage/ or exp Intracranial Hemorrhages/
2	Intracerebral hemorrhage.mp.
3	1 or 2
4	exp cerebrovascular accident/di [Diagnosis]
5	exp stroke/di
6	4 or 5
7	(recogni$ or identi$ or strati$ or distinguish$ or probabilit$).mp.
8	3 and 7
9	6 or 8
10	exp Emergency Medical Services/ or Paramedic*.mp. or ambulance.mp. or triage.mp. or prehospital.mp. or pre hospital.mp. or EMS.mp. or emergency medical service*.mp. or out-of-hospital.mp.
11	Air Ambulances/
12	Emergency Service, Hospital/
13	Stroke Score.mp.
14	Clinical differentiation.mp.
15	10 or 11 or 12 or 13 or 14
16	9 and 15
17	limit 16 to (human and english language)

For peer review only

Database searched: CENTRAL (Ovid)

#	Query
1	exp Brain Ischemia/ or exp Stroke/ or exp Hemorrhagic Stroke/ or exp Cerebral Hemorrhage/ or exp Intracranial Hemorrhages/
2	Intracerebral hemorrhage.mp.
3	1 or 2
4	exp cerebrovascular accident/di
5	exp stroke/di
6	4 or 5
7	(recogni$ or identi$ or strati$ or different$ or probabilit$).mp.
8	3 and 7
9	6 or 8
10	exp Emergency Medical Services/ or Paramedic*.mp. or ambulance.mp. or triage.mp. or prehospital.mp. or pre hospital.mp. or EMS.mp. or emergency medical service*.mp.
11	Air Ambulances/
12	Emergency Service, Hospital/
13	Emergency Medicine/
14	Clinical feature.mp.
15	Stroke Score.mp.
16	Clinical differentiation.mp.
17	10 or 11 or 12 or 13 or 14 or 15 or 16
18	9 and 17
19	limit 18 to english language

For peer review only

2- The search strategy for portable devices

Database searched: MEDLINE (Ovid)

#	Query
1	exp Brain Ischemia/ or exp Stroke/ or exp Hemorrhagic Stroke/ or exp Cerebral Hemorrhage/ or exp Intracranial Hemorrhages/
2	Intracerebral hemorrhage.mp.
3	1 or 2
4	(recogni$ or identi$ or strati$ or diagnos$ or detect$).mp.
5	exp Emergency Medical Services/ or Paramedic*.mp. or ambulance.mp. or triage.mp. or prehospital.mp. or pre hospital.mp. or EMS.mp. or emergency medical service*.mp.
6	Air Ambulances/
7	Emergency Service, Hospital/
8	(technology or spectroscopy or ultrasound or diagnostic imaging or imaging or transcranial doppler or radiofrequency or microwave or electroencephalography or device).mp.
9	5 or 6 or 7
10	3 and 4 and 8 and 9
11	limit 10 to (english language and humans)

For peer review only

Database searched: EMBASE (Ovid)

#	Query
1	exp Brain Ischemia/ or exp Stroke/ or exp Hemorrhagic Stroke/ or exp Cerebral Hemorrhage/ or exp Intracranial Hemorrhages/
2	Intracerebral hemorrhage.mp.
3	1 or 2
4	(recogni$ or identi$ or strati$ or diagnos$ or detect$).mp.
5	exp Emergency Medical Services/ or Paramedic*.mp. or ambulance.mp. or triage.mp. or prehospital.mp. or pre hospital.mp. or EMS.mp. or emergency medical service*.mp.
6	Air Ambulances/
7	Emergency Service, Hospital/
8	(technology or spectroscopy or ultrasound or diagnostic imaging or imaging or transcranial doppler or radiofrequency or microwave or electroencephalography or device).mp.
9	5 or 6 or 7
10	3 and 4 and 8 and 9
11	limit 10 to (english language and humans)

For peer review only

Database searched: CENTRAL (Ovid)

#	Query
1	exp Brain Ischemia/ or exp Stroke/ or exp Hemorrhagic Stroke/ or exp Cerebral Hemorrhage/ or exp Intracranial Hemorrhages/
2	Intracerebral hemorrhage.mp.
3	1 or 2
4	(recogni$ or identi$ or strati$ or diagnos$ or detect$).mp.
5	exp Emergency Medical Services/ or Paramedic*.mp. or ambulance.mp. or triage.mp. or prehospital.mp. or pre hospital.mp. or EMS.mp. or emergency medical service*.mp.
6	Air Ambulances/
7	Emergency Service, Hospital/
8	(technology or spectroscopy or ultrasound or diagnostic imaging or imaging or transcranial doppler or radiofrequency or microwave or electroencephalography or device).mp.
9	5 or 6 or 7
10	3 and 4 and 8 and 9
11	limit 10 to (english language and humans)

For peer review only

BMJ Open

Prehospital identification of intracerebral haemorrhage: a scoping review of early clinical features and portable devices

Journal:	*BMJ Open*
Manuscript ID	bmjopen-2023-079316.R1
Article Type:	Original research
Date Submitted by the Author:	01-Mar-2024
Complete List of Authors:	Almubayyidh, Mohammed; The University of Manchester, Division of Cardiovascular Sciences; King Saud University, Prince Sultan Bin Abdulaziz College for Emergency Medical Services, Department of Aviation and Marine<br>Alghamdi, Ibrahim; The University of Manchester, Division of Cardiovascular Sciences; King Khalid University, Department of Emergency Medical Services<br>Parry-Jones, Adrian; The University of Manchester, Division of Cardiovascular Sciences; Northern Care Alliance NHS Foundation Trust, Manchester Centre for Clinical Neurosciences<br>Jenkins, David; The University of Manchester, Division of Informatics, Imaging and Data Science
**Primary Subject Heading**:	Emergency medicine
Secondary Subject Heading:	Neurology
Keywords:	Stroke < NEUROLOGY, STROKE MEDICINE, ACCIDENT & EMERGENCY MEDICINE

SCHOLARONE™
Manuscripts

I, the Submitting Author has the right to grant and does grant on behalf of all authors of the Work (as defined in the below author licence), an exclusive licence and/or a non-exclusive licence for contributions from authors who are: i) UK Crown employees; ii) where BMJ has agreed a CC-BY licence shall apply, and/or iii) in accordance with the terms applicable for US Federal Government officers or employees acting as part of their official duties; on a worldwide, perpetual, irrevocable, royalty-free basis to BMJ Publishing Group Ltd ("BMJ") its licensees and where the relevant Journal is co-owned by BMJ to the co-owners of the Journal, to publish the Work in this journal and any other BMJ products and to exploit all rights, as set out in our licence.

The Submitting Author accepts and understands that any supply made under these terms is made by BMJ to the Submitting Author unless you are acting as an employee on behalf of your employer or a postgraduate student of an affiliated institution which is paying any applicable article publishing charge ("APC") for Open Access articles. Where the Submitting Author wishes to make the Work available on an Open Access basis (and intends to pay the relevant APC), the terms of reuse of such Open Access shall be governed by a Creative Commons licence – details of these licences and which Creative Commons licence will apply to this Work are set out in our licence referred to above.

Other than as permitted in any relevant BMJ Author's Self Archiving Policies, I confirm this Work has not been accepted for publication elsewhere, is not being considered for publication elsewhere and does not duplicate material already published. I confirm all authors consent to publication of this Work and authorise the granting of this licence.

For peer review only

Prehospital identification of intracerebral haemorrhage: a scoping review of early clinical features and portable devices

Mohammed Almubayyidh[1,2*], Ibrahim Alghamdi[1,3], Adrian Parry-Jones[1,4], David A Jenkins[5]

[1]Division of Cardiovascular Sciences, The University of Manchester, Manchester, UK

[2]Department of Aviation and Marine, Prince Sultan Bin Abdulaziz College for Emergency Medical Services, King Saud University, Riyadh, Saudi Arabia

[3]Department of Emergency Medical Services, College of Applied Medical Sciences, Khamis Mushait Campus, King Khalid University, Abha, Saudi Arabia

[4]Manchester Centre for Clinical Neurosciences, Northern Care Alliance NHS Foundation Trust, Salford, UK

[5]Division of Informatics, Imaging and Data Science, The University of Manchester, Manchester, UK

*Corresponding author:

Mohammed Almubayyidh, mohammed.almubayyidh@postgrad.manchester.ac.uk, Division of Cardiovascular Sciences, The University of Manchester, Manchester, UK

Abstract word count: 278

Text word count: 5234

For peer review only - http://bmjopen.bmj.com/site/about/guidelines.xhtml

For peer review only

ABSTRACT

Introduction

Prehospital identification of intracerebral haemorrhage (ICH) in suspected stroke cases may enable the initiation of appropriate treatments and facilitate better-informed transport decisions. This scoping review aims to examine the literature to identify early clinical features and portable devices for the detection of ICH in the prehospital setting.

Methods

Three databases were searched via Ovid (MEDLINE, EMBASE, and CENTRAL) from inception to August 2022 using pre-specified search strategies. One reviewer screened all titles, abstracts, and full-text articles for eligibility, while a second reviewer independently screened 20% of the literature during each screening stage. Data extracted were tabulated to summarise the key findings.

Results

A total of 6,803 articles were screened for eligibility, of which 22 studies were included for analysis. Among them, 15 studies reported on early clinical features, while 7 considered portable devices. Associations between age, sex, and comorbidities with the presence of ICH varied across studies. However, most studies reported that patients with ICH exhibited more severe neurological deficits (n=6) and higher blood pressure levels (n=11) at onset compared to other stroke and non-stroke diagnoses. Four technologies were identified for ICH detection: microwave imaging technology, volumetric impedance phase-shift spectroscopy, transcranial ultrasound, and electroencephalography. Microwave and ultrasound imaging techniques showed promise in distinguishing ICH from other diagnoses.

Conclusion

This scoping review has identified potential clinical features for the identification of ICH in suspected stroke patients. However, the considerable heterogeneity among the

For peer review only

For peer review only - http://bmjopen.bmj.com/site/about/guidelines.xhtml

included studies precludes meta-analysis of available data. Moreover, we have explored portable devices to enhance ICH identification. While these devices have shown promise in detecting ICH, further testing is required to assess their ability to differentiate between stroke subtypes (ICH vs ischaemic stroke) and non-stroke diagnoses.

Keywords: Prehospital; Stroke; Intracerebral haemorrhage; Clinical features; Technology; Differential diagnosis.

STRENGTHS AND LIMITATIONS OF THIS STUDY

- To the best of our knowledge, this is the first review focused on identifying spontaneous intracerebral haemorrhage (ICH) in the prehospital setting.
- No time of publication restrictions were applied to comprehensively map the available evidence.
- The review is limited to English language publications only.
- Consistent with the scoping review methodology, a formal risk of bias assessment was not conducted.

For peer review only

INTRODUCTION

Prehospital recognition of intracerebral haemorrhage (ICH) among suspected stroke cases is not part of standard care, but the ability to reliably do so may pave the way for much quicker delivery of time-critical treatments and triage to a hospital that can provide appropriate interventions (e.g., hyperacute stroke care, neurosurgery) [1,2]. The importance of the rapid identification of ICH patients has been highlighted by studies showing that early haematoma expansion (HE) frequently occurs within 2–3 hours of onset [2-4]. Thus, lowering blood pressure (BP) as well as administering anticoagulant reversal/haemostatic agents may limit HE, thereby increasing the likelihood of favourable outcomes [2,5]. These treatment strategies are currently under investigation in prehospital settings [6-8]. Facilitating the diagnosis of ICH in ambulances through simple and cost-effective methods could support the broader implementation of such therapeutic trials in the future.

While the initial diagnosis of stroke in the prehospital setting is based on stroke recognition tools (e.g., FAST—Face Arm Speech Test, CPSS—Cincinnati Prehospital Stroke Scale), these tools have been validated to identify all strokes rather than ICH specifically [9]. Recognising the importance of timely and accurate identification of ICH, the American Heart Association/American Stroke Association's recent guidelines for the management of patients with ICH have highlighted the need to improve the prehospital recognition of ICH [9]. The recent RACECAT trial further emphasises the significance of prehospital decisions in stroke management, which found that bypassing the closest stroke centre for direct transport to a thrombectomy-capable centre was associated with worse outcomes for patients with ICH [10]. This approach could be more beneficial for patients with large vessel occlusion (LVO) [11], underscoring the necessity for the prehospital differentiation of ICH, especially from LVO [12].

Several clinical features were found to be predictive of ICH in patients with stroke who experience persistent symptoms for more than 24 hours [13]. These features were used to develop clinical prediction rules to distinguish ICH from ischaemic stroke (IS) [13,14]. Nevertheless, the diagnostic accuracy of these rules is low, and they may not

For peer review only - http://bmjopen.bmj.com/site/about/guidelines.xhtml

be applicable to patients presenting within a few hours of the onset of symptoms or during the prehospital phase [15,16]. Such tools for the identification of ICH may have the potential to improve patient outcomes. However, it is unclear which clinical features could be used to identify ICH in prehospital settings. Consequently, brain imaging (computed tomography [CT] and/or magnetic resonance imaging [MRI]) is the only accepted method for diagnosing ICH and other subtypes of stroke. This has led to the development of new portable imaging devices for the detection of patients with stroke in prehospital settings [17,18].

The aim of this study is to perform a scoping review to summarise the early distinguishing clinical features of ICH and evaluate novel portable devices that can help prehospital personnel differentiate ICH from other causes of suspected stroke.

For peer review only

METHODS

The scoping review followed the guidelines of the Joanna Briggs Institute (JBI) [19,20]. It was reported according to the Preferred Reporting Items for Systematic Reviews and Meta-Analyses Extension for Scoping Reviews (PRISMA-ScR) checklist (Appendix 1) [21]. We adopted a scoping review approach because of a broad set of inclusion criteria. A detailed study protocol was published previously [22].

Eligibility criteria

The eligibility criteria for this review were classified using the 'Population, Concept, Context' framework suggested by the JBI (Table 1) [19]. The framework is applied as follows:

- Population: Adult patients (aged ≥16 years) with suspected stroke confirmed by either CT or MRI scans. Studies were excluded if they enrolled children (aged <16 years) or combined the clinical features of spontaneous ICH patients with those of other conditions (e.g., subarachnoid haemorrhage [SAH]) in the same group without reporting them separately.
- Concept: First, to distinguish spontaneous ICH from other stroke and non-stroke diagnoses by investigating the early clinical features observed during the prehospital phase or within the initial 24 hours after the onset of

symptoms upon arrival at the hospital. Second, to explore the use of portable devices in the detection and classification of ICH. Studies were excluded if they reported clinical features of ICH after 24 hours of symptoms onset, or if they investigated clinical features associated with ICH without comparing them against other suspected stroke cases. Further studies excluded from the review included those that did not provide information on the diagnostic accuracy of the tested technology, examined advances in conventional imaging techniques (CT and MRI), or exclusively tested the technology on phantoms or animal models.

- Context: Prehospital and in-hospital settings with no restrictions on the country of study, ethnicity, gender, or socioeconomic status. Studies conducted in primary care settings were excluded.

Sources of information and search strategy

We included data from peer-reviewed primary research studies published in English. Conference abstracts, commentaries, surveys, case reports, preprints, animal studies, and non-English-language papers were excluded. The literature search of MEDLINE (via Ovid), EMBASE (via Ovid), and CENTRAL (via Ovid) databases was conducted in August 2022, using our pre-specified search strategies (Appendix 2) [22]. To ensure that all relevant studies were included, there was no restriction regarding the date of publication. In addition, we searched the reference lists of retrieved papers to identify additional relevant studies for this review.

Study selection

To eliminate duplicates and facilitate screening and collaboration, the identified studies were imported into EndNote and Rayyan Qatar Computing Research Institute (QCRI) software [23]. Two independent reviewers (MA and IA) selected eligible studies in two stages; initially, titles and abstracts were screened, and then full texts were assessed. MA screened all titles, abstracts, and full-text articles against the eligibility criteria. IA screened a random sample of 20% of the studies at both the title and abstract and full-text stages. Any disagreements in any phase of the selection process

For peer review only - http://bmjopen.bmj.com/site/about/guidelines.xhtml

were resolved by discussion between reviewers to reach consensus or by a third reviewer (APJ or DAJ).

Data extraction

Two data extraction forms (Supplementary Tables 1 and 2) were created using Microsoft Excel, considering the review objectives, the content of the included studies, and insights from previous reviews [15,24]. These tables were designed to present the data from the different studies in a standard format, allowing comparisons to be made between studies and their findings. The data were extracted by the first reviewer (MA), and the second reviewer (IA) independently checked 20% of the extracted data to ensure accuracy.

Data synthesis and quality assessment of studies

Data were summarised as median, mean, and range when appropriate. Otherwise, a narrative approach was used to synthesise the findings of the included studies. In addition, since the PRISMA-ScR checklist does not require an assessment of bias [21], a formal risk of bias assessment was not conducted.

Patient and public involvement

No patients or public were involved in this study.

RESULTS

The search strategies yielded a total of 10,490 articles; after removing duplicates, 6,803 articles remained (Figure 1). Following the initial screening of titles and abstracts, 182 articles remained as potentially relevant and were retrieved for full-text review. After obtaining and reviewing the full texts, 162 studies were excluded with reasons, as illustrated in a PRISMA flowchart in Figure 1. The manual search of the reference lists of included studies revealed two additional studies, resulting in a total of 22 included studies.

For peer review only - http://bmjopen.bmj.com/site/about/guidelines.xhtml

Description of included studies

The retrieved studies were divided into two groups based on the aims of the scoping review: studies that reported the early clinical features of ICH patients (n=15) [25-39] and those that used portable devices to detect ICH (n=7) [40-46]. These groups were divided further according to the data extracted from them.

In the first group (Supplementary Table 1), studies compared the clinical features among various populations, including stroke subtypes (n=12) and non-stroke diagnoses (n=3). Different statistical analyses were used in these studies to determine clinical features, such as univariable (n=3) and multivariable (n=12) regression analyses. Regardless of the statistical techniques used, the reported clinical features of interest were arranged during the analysis process into three groups (Table 2): patients' general information and medical history, early signs and symptoms, and vital signs. Studies that employed portable devices (Supplementary Table 2) to detect or differentiate ICH and other diagnoses were divided into four sub-groups based on the underpinning technology: microwave imaging technology (n=1), volumetric impedance phase shift spectroscopy (VIPS) (n=1), transcranial ultrasound (n=3), and electroencephalography (EEG) (n=2). Additionally, most studies were conducted in Europe (n=13), followed by North America (n=5) and Asia (n=4).

Prediction of ICH in suspected stroke patients using early clinical features

Patients' general information and medical history

Several studies found that patients with ICH were younger (Supplementary Table 1) [25-27,33,36-38], and more likely to be male [29,35,36,38] than those with other stroke and non-stroke diagnoses. However, this association was not uniformly reported, as other studies found that neither age [28,29,31,35,39], nor sex [25-28,31,33,34,39], were predictors of the final diagnosis of ICH. Only one study identified an association between older age and increasing probability of posterior fossa ICH [34].

Several conditions or factors were identified as being more strongly associated with the presence of ICH (vs other diagnoses), including a history of hypertension (HTN)

For peer review only

[30,34,38], prior ICH [26,31,36], haemodialysis [26], alcohol consumption [36] and the use of warfarin [34]. Conversely, patients with ICH were less likely to have a history of atrial fibrillation (AF) [26,27,29,34-36,38], coronary artery disease (CAD) [25,26,36,38,39], dyslipidaemia [26,34,36,37], diabetes mellitus (DM) [25,27], peripheral arteriopathy (PAD) [36], prior transient ischaemic attack (TIA) [33] or IS [28]. However, other studies found no or weak associations between ICH and HTN [26,27,29,33,35-37], prior TIA/IS [26,36], history of ICH [28], AF [25,33,37,39], CAD [33-35], dyslipidaemia [25,35,38,39], DM [26,31,33-39], PAD [35], alcohol consumption [35,38], or warfarin use [28]. In two studies [25,39], a history of HTN was found to be more common among IS patients than ICH.

Early signs and symptoms of ICH

Several symptoms were reported at the onset of ICH. Among them, headache [26,33,34] and vomiting [26,27,34] were more frequently recorded; however, three studies [25,28,30] did not find these symptoms to be specific to ICH. Additionally, five studies reported that impaired level of consciousness (LOC) was more prevalent among patients with ICH [25,26,29,32,33]. Meanwhile, two studies [28,30] found decreased LOC was not only associated with ICH, but also with LVO and SAH.

Seizures [26] and neurological deficits [26,29], including sensory or motor deficits [26,29], difficulty in following commands [26,29] and impaired vision [29] were also common presentations. Yet six studies [25,28-30,33,34] did not find the presence of seizures [25,29,34], or neurological deficits [25,28,30,33] to be specific discriminators of ICH. In terms of neurological deficits, one study found that compared to IS patients, ICH patients less frequently woke up with symptoms and they experienced greater leg weakness [33].

Eight studies [26,29,34-39] used the National Institutes of Health Stroke Scale (NIHSS) to assess stroke severity either in the prehospital setting [29] or at hospital admission [26,34-39]. Among these studies, six reported higher NIHSS scores at presentation in patients with ICH compared to those with IS, with median scores ranging from 11 to 17 for ICH patients and 6 to 13 for IS patients [26,29,35-38]. However, two studies found no discernible difference in NIHSS scores between ICH and IS patients [34,39].

For peer review only - http://bmjopen.bmj.com/site/about/guidelines.xhtml

Vital signs and their predictive capabilities of ICH

Most of the studies investigated vital signs, including systolic and diastolic BP (SBP and DBP, respectively) [25-29,31-35,37,38], mean arterial pressure (MAP) [29,31], heart rate (HR) or rhythm [25-28,33], peripheral oxygen saturation (SPO_2) [33], and blood glucose level [34,35,38].

In four studies that investigated the prehospital BP [27-29,33], the BP values were dichotomised as follows: SBP, >200 mm Hg [33], ≥180 mm Hg [27,29], ≥165 mm Hg [28]; DBP, ≥110 mm Hg [29], ≥100 mm Hg [27], ≥95 mm Hg [28], >90 mm Hg [33]; MAP, ≥130 mm Hg [29]. These studies found that a higher proportion of ICH patients had elevated BP at presentation than those with other stroke subtypes and non-stroke conditions.

Six studies reported that mean or median SBP [26,31,34,35,37,38], DBP [26,31,34,35,38], and MAP [31] values of ICH patients were higher at the time of onset than in patients with other diagnoses (SBP ranged from 160–204 mm Hg, DBP ranged from 84–102 mm Hg and the MAP was 122.0 ± 20.2 mm Hg). Only one study [25] found no differences in the SBP and DBP values of ICH and IS patients in the prehospital setting. However, another study found that elevated SBP (≥200 mm Hg) in the prehospital setting among patients with stroke with impaired LOC was strongly associated with an increased risk of ICH [32].

With the exception of five studies, no differences were detected in vital signs such as HR [27,33] or rhythm [25,33], SPO_2 [33] and blood glucose [35] between ICH and IS patients. Three of the five studies reported that arrhythmia was less likely to be associated with ICH [26-28], and two observed higher median blood glucose levels at the time of ICH onset (7.1–7.4 mmol/L) compared to IS (6.7–6.8 mmol/L) [34,38].

Portable devices to detect and differentiate ICH

Microwave imaging technology

Two pilot studies were conducted to assess the diagnostic accuracy of microwave-based prototype devices in distinguishing ICH from IS, as well as ICH from healthy individuals [40]. In the first study, 20 patients with stroke were examined within 7–

For peer review only - http://bmjopen.bmj.com/site/about/guidelines.xhtml

132 hours of onset, using the first prototype device by engineering and neurophysiology staff. When aimed at identifying all patients with ICH, the haemorrhagic detector correctly differentiated 7 out of 11 IS patients from the ICH group, with an area under curve (AUC) of 0.88.

The second pilot study involved 25 patients with stroke within 4–27 hours of onset and 65 healthy individuals, with nursing staff testing the second custom-built helmet. When aimed at identifying all patients with ICH, the haemorrhagic detector accurately distinguished 14 out of 15 IS patients from the ICH group, achieving an AUC of 0.85 for distinguishing ICH from IS and 0.87 for distinguishing ICH patients from healthy individuals.

Volumetric impedance phase shift spectroscopy (VIPS)

The effectiveness of the VIPS visor device, developed by Cerebrotech, was investigated in a study aiming to assess its capability to measure the bioimpedance of each brain hemisphere for determining stroke severity [41]. The study enrolled 248 participants, including 41 'code stroke' patients, 79 healthy controls, and 128 patients with various neurological conditions at a comprehensive stroke centre. Trained personnel conducted three scans using the visor device, with a total scan duration of approximately 30 seconds.

The study aimed to evaluate the device's performance in distinguishing severe strokes from minor strokes and severe strokes from all other subjects. Severe strokes were defined as those requiring triage to a comprehensive stroke centre, including conditions such as LVO, NIHSS score of ≥6, ICH of ≥60 ml, and large territorial strokes.

The results of the study demonstrated that patients with severe strokes exhibited the highest mean bioimpedance asymmetry. The VIPS visor device achieved a sensitivity of 93% (95% confidence interval [CI]: 83–98%), specificity of 92% (95% CI: 75–99%), and an AUC of 0.93 (95% CI: 0.85–0.97) when differentiating severe strokes from minor strokes. Similarly, in distinguishing severe strokes from all tested subjects, the device showed a sensitivity of 93% (95% CI: 83–98%), a specificity of 87% (95% CI: 81–92%), and an AUC of 0.93 (95% CI: 0.89–0.96). The ability of VIPS to differentiate ICH from other conditions was not reported in this study.

For peer review only - http://bmjopen.bmj.com/site/about/guidelines.xhtml

Transcranial ultrasound

Three studies utilised transcranial ultrasound imaging to diagnose ICH and distinguish it from other suspected stroke causes [42-44]. In one clinical study [42], transcranial colour-coded duplex sonography (TCCS) scans were conducted on 84 patients using a Sonos 1000 Hewlett-Packard machine. ICH diagnosis was based on sharply demarcated hyperechogenic areas in the brain tissue. Among the patients, 52 had IS or TIA, 15 had ICH, and 17 were excluded due to inadequate temporal bone windows. TCCS identified ICH in 14 patients with focal hyperechogenicity, with only one small haemorrhage missed. Sensitivity and specificity were reported as 88% and 96%, respectively.

In another study [43], the diagnostic accuracy of TCCS was evaluated using Siemens Sonoline Ellegra or Acuson 128 XP/4 on 151 patients with acute hemiparesis. Among them, 60 had ICH, 67 had IS, and 24 had inconclusive CT scans. Ultrasound examination of 18 patients revealed insufficient acoustic bone windows. For the remaining 133 patients, TCCS detected 50 cases of ICH with a sensitivity of 94% and specificity of 95%.

A third study examined 107 suspected stroke patients using SonoSite M-Turbo, Philips Sparq, or Philips CX50 for TCCS [44]. Among the patients, 63 had acute IS, 18 had ICH, 13 had TIA, and 13 had stroke-mimicking conditions. Sonographic examination revealed an insufficient temporal bone window in 18 cases. Ultrasound imaging accurately identified ICH in 56% of patients, but failed in 33% of cases. The study presented a triage model that combined transcranial ultrasound and clinical assessment, resulting in a 10% improvement in ICH detection compared to clinical assessment alone. While the specificity of both techniques was reported as 99%, the sensitivity of the combined model was 63%, whereas clinical assessment alone yielded a sensitivity of 6%.

Electroencephalography (EEG)

Two studies examined the diagnostic capability of portable EEG devices for detecting strokes in emergency departments (ED) [45,46]. In one study [45], BrainScope's handheld device was used to identify strokes in 183 patients presenting with stroke-like symptoms. Among them, 31 patients were diagnosed with IS, 17 with

For peer review only - http://bmjopen.bmj.com/site/about/guidelines.xhtml

haemorrhagic stroke, and 135 with stroke-mimicking conditions. The device recorded EEG data for 10 minutes while the patients kept their eyes closed, and the analysis was conducted using the Structural Brain Injury Index (SBII) algorithm. The SBII showed a sensitivity of 94.1% for detecting haemorrhagic strokes, with a specificity of 50.4% for stroke mimics serving as controls.

Another study assessed the Quick-20 EEG system for diagnosing patients with acute stroke [46]. Out of the 100 patients involved, 63 were diagnosed with acute stroke/TIA, among whom 7 patients had ICH. EEG signals alone achieved a sensitivity of 65% and specificity of 80%, with an AUC of 78.2 for detecting acute stroke/TIA. Combining EEG with clinical data using deep learning improved diagnostic performance, yielding a sensitivity of 79%, a specificity of 80%, and an AUC of 87.8.

DISCUSSION

Overall, we identified fifteen studies that reported early clinical features of ICH among suspected stroke patients and seven studies that tested portable technologies to detect ICH and other conditions.

Early clinical features of ICH in suspected stroke patients

Diagnosing ICH based solely on clinical features can be difficult, as many features of ICH overlap with other types of strokes [47]. This scoping review has identified potential clinical features that can help distinguish ICH patients from those with other stroke types or non-stroke diagnoses. The combination of these features might be useful in improving the early prediction of ICH. However, it is important to note that the included studies investigated and reported different clinical features for ICH patients across various stroke subtypes, and some studies also included TIA and stroke-mimicking conditions in their analyses. In addition, some studies only conducted univariable analysis, and caution is advised when interpreting our results as there could be bias in the studies, such as confounding. Heterogeneity in the definition and categorisation of variables was also observed. Owing to these methodological limitations, we recognise that conducting a meta-analysis of individual

For peer review only - http://bmjopen.bmj.com/site/about/guidelines.xhtml

features would be highly challenging and unlikely to yield meaningful insights relevant to the aims of this review.

Given the considerable heterogeneity among the included studies, identifying consistent predictors for the diagnosis of ICH was challenging. In terms of patients' general information and medical history, neither age nor sex consistently predicted the final diagnosis of ICH across the studies. However, certain factors were reported to be more strongly associated with the presence of ICH, including a history of HTN, prior ICH, haemodialysis, alcohol consumption, and the use of warfarin. In contrast, patients with ICH were less frequently found to have a history of AF, CAD, dyslipidaemia, DM, PAD, prior TIA, or IS. If available in the prehospital setting, electrocardiogram monitoring can be used to assess the presence of AF, which may help differentiate ICH from IS [26,29,48,49]. Nevertheless, the associations between these factors and ICH varied across studies. These results contradict a previous meta-analysis [15] that found a greater likelihood of ICH in male patients aged 60 years or less, while a history of TIA, PAD, and AF increased the probability of IS being diagnosed.

The initial signs and symptoms of ICH, such as headache, vomiting, and decreased LOC, also demonstrated variability across the included studies. While these manifestations were frequently observed, some studies did not find them to be exclusively indicative of ICH. This discrepancy could potentially be attributed to the inclusion of patients with SAH in certain studies [28,30]. SAH patients typically present with a sudden onset of headache, vomiting, decreased LOC, and neck stiffness [50,51]. Consequently, relying solely on these clinical features for the prediction of ICH may lack specificity when compared to SAH patients.

The majority of studies in this review consistently reported that patients with ICH experienced more severe neurological deficits at onset compared to other subtypes of stroke, TIA, and stroke-mimicking conditions. Furthermore, ICH patients were less commonly observed to have neurological deficits upon awakening when compared to patients with IS. These findings are in line with other studies that have reported a higher frequency of hemiplegia/hemiparesis and sensory deficits in patients with

For peer review only - http://bmjopen.bmj.com/site/about/guidelines.xhtml

cerebral haemorrhage [48,50], while stroke symptoms upon awakening were more frequently observed in patients with IS [50,52].

To assess and quantify neurological deficits, more than half of the studies utilised the NIHSS, which consistently showed higher scores in ICH patients. In fact, a recent study investigating the relationship between early NIHSS scores and ICH volume found that for every 10 ml increase in ICH volume, the NIHSS score increased by 4.5 points [53]. However, despite this, the NIHSS is not routinely used by prehospital personnel for stroke assessment due to its complexity and time-consuming nature [54]. In an effort to address these limitations, a recent study demonstrated that the NIHSS can be an accurate and time-efficient tool for paramedics in the field [55], suggesting the potential of utilising the NIHSS items to construct a prehospital prediction model for ICH [29].

Several prediction models have been proposed to differentiate ICH from other suspected stroke patients in the prehospital setting [25,27-30]. These tools can assist prehospital personnel in making accurate and timely decisions for patients with stroke. However, their predictive abilities have not been thoroughly studied, compared, and evaluated in an independent study. Therefore, further research is necessary to assess their performance, reliability, and potential limitations or biases that may impact their effectiveness in real-world settings. The addition of the clinical features found in this review could further improve the performance of these tools.

Another important finding of this study is that patients with ICH had the highest BP parameters among all suspected stroke patients. Notably, there is a strong correlation between elevated SBP of ≥160 mm Hg and the initial haematoma volume in ICH patients [56]. A post-hoc analysis of the "FAST-MAG" trial revealed a distinct association between SBP and ultra-early neurological deterioration (defined as a deterioration of 2 or more points on the Glasgow Coma Scale within 2 hours of stroke onset) among ICH patients [57]. According to the study, neurological deterioration in patients with ICH was associated with higher SBP upon arrival at the ED. Additionally, imaging performed upon arrival showed that ICH patients with neurological deterioration had much larger haematoma volumes, suggesting that early HE may contribute to the deterioration [57]. Previous studies have also found an association

For peer review only - http://bmjopen.bmj.com/site/about/guidelines.xhtml

between prehospital BP values (SBP and DBP) and the occurrence of neurological deterioration in ICH [58,59]. These findings partially align with our results, in which there was a very powerful relationship between increased prehospital SBP and the occurrence of ICH among patients with stroke with impaired LOC [32].

Other vital signs, such as HR and $SPO_{2,}$ were compared in some of the included studies and seemed to be insignificant in predicting the presence or absence of ICH. However, associations appear evident between cardiac arrhythmias in patients with IS or LVO, while higher blood glucose levels were reported in patients with ICH upon hospital admission.

Portable technologies for ICH detection and differentiation

Numerous portable technologies have been designed to identify and distinguish ICH from other stroke subtypes or stroke-mimicking diagnoses. In addition to clinical features, this scoping review has identified portable devices that have the potential to improve the detection of ICH and could be used in various settings, including prehospital care.

In recent years, significant progress has been made in the prehospital diagnosis and treatment of ICH patients with the introduction of mobile stroke units (MSUs) [2,60,61]. However, due to the high costs associated with this technology, it is unlikely to become widely available for stroke care in the near future [62]. Thus, portable devices may serve as cost-effective alternatives to MSUs in the prehospital setting.

Some of the technologies evaluated showed greater accuracy than clinical assessments [41,44,46]. Antipova et al. [44] developed a triage model that combined transcranial sonographic findings with clinical assessment, resulting in significantly improved diagnostic sensitivity for detecting ICH. Hence, the combination of clinical features with the findings of these systems might further enhance the diagnostic accuracy of ICH.

The reviewed studies also highlighted the potential benefits of using these technologies for rapid diagnosis in the prehospital setting. For instance, in the VITAL study [41], the VIPS technology demonstrated an average detection time of 30

For peer review only - http://bmjopen.bmj.com/site/about/guidelines.xhtml

seconds for severe strokes. Other technologies evaluated had median application times ranging from 10 to 20 minutes (Supplementary Table 2), depending on the specific technology employed. Shortening the time-to-treatment duration is crucial in stroke management to improve patient outcomes [63]. Therefore, it is important to ensure that the use of diagnostic technologies does not cause delays in transporting patients to definitive care.

A number of limitations were identified for each technology. Most notably, the discriminatory ability to differentiate ICH from other diseases was not evaluated with VIPS and portable EEGs [41,45,46]. Conversely, microwave and ultrasound imaging techniques yielded good results in differentiating between stroke subtypes [40,42-44]. However, the diagnostic accuracy of microwave technology to discriminate ICH from IS could be decreased with the development of cerebral oedema after IS [40]. On the other hand, transcranial ultrasound cannot produce useful images unless adequate temporal bone windows are available [42-44]. Furthermore, with this technology, pathological conditions such as haemorrhagic transformation of infarcts and gliomas could be misdiagnosed as ICH [42,43].

While several diagnostic modalities discussed in this review show promise for prehospital stroke care, their diagnostic accuracy requires further improvement and validation using a large, diverse cohort of suspected stroke patients. Such validation is crucial to establish their clinical usefulness in the prehospital setting, where both sensitivity and specificity are important in determining appropriate management. Given the significant differences in treatment strategies between ICH and IS, it is imperative that the detection technologies can effectively differentiate between the two with high sensitivity and specificity. Reliable differentiation can enable the prompt initiation of appropriate treatment measures for ICH, including BP reduction and anticoagulation reversal, and facilitate triaging these patients to neurosurgical-capable facilities. Moreover, consideration should be given to the level of training required for personnel to competently operate these technologies [24]. For instance, devices such as transcranial ultrasound necessitate highly trained operators, which may limit their feasibility in the prehospital setting.

For peer review only - http://bmjopen.bmj.com/site/about/guidelines.xhtml

Other novel approaches to detect and distinguish ICH

There are other novel technologies that exist for detecting and distinguishing ICH, but these fall beyond the scope of this review, either due to the technologies not being investigated in suspected stroke cases or their lack of portability. One of the most studied among them is near-infrared spectroscopy (NIRS). In a pilot study, the handheld NIRS Infrascanner was evaluated for its ability to detect intracranial haematomas in 35 patients with traumatic brain injuries [64]. When compared to CT scans, which were considered the gold standard, the Infrascanner demonstrated a sensitivity of 88.89% (95% CI: 50.7–99.4%) and specificity of 81.25% (95% CI: 53.7–95.0%) in identifying intraparenchymal haematomas. However, its effectiveness in accurately distinguishing between stroke subtypes, particularly in identifying deep ICH, may be hindered by its limited detection range of approximately 3 cm beneath the skull [64,65].

Besides brain diagnostic devices, the use of blood biomarkers is also a promising approach for the early detection and differentiation of stroke subtypes [37,66]. However, it remains unclear whether they can be used as a point-of-care diagnostic method in the prehospital setting [67]. This is currently being investigated in a prehospital trial (Biomarkers for Initiating Onsite and Faster Ambulance Stroke Therapies, Bio-FAST, ClinicalTrials.gov Identifier: NCT04612218) with point-of-care test devices.

Limitations of the review

Our scoping review has some limitations that should be considered. Only one reviewer screened and analysed all of the data. Although a second reviewer independently screened 20% of the articles at the abstract, full-text, and data extraction stages, there is a risk of review errors and bias. Additionally, the search was limited to English publications, which could have resulted in the exclusion of important studies published in other languages. Furthermore, some of the included studies examined the clinical features of ICH up to 24 hours of symptoms onset, which requires cautious interpretation of the findings regarding their external validity for prehospital evaluations. Lastly, owing to the nature of scoping reviews, the methodological quality

For peer review only - http://bmjopen.bmj.com/site/about/guidelines.xhtml

of the studies included was not assessed [20]. This means that poor-quality studies were given equal weight as high-quality studies, which should be taken into account when interpreting the review findings.

CONCLUSION AND FUTURE WORK

Our study identifies potential early clinical features of ICH that distinguish it from suspected stroke patients. However, the substantial heterogeneity observed in populations, clinical features, and outcome variables among the included studies precludes the combination of these features in a meta-analysis. The variability in findings across studies highlights the need for further research and standardised tools to improve early prediction and differentiation of ICH. Future studies should focus on developing and validating predictive models or tools that incorporate easily obtainable clinical features for prehospital personnel. Additionally, it is important to consider an unselected cohort of suspected stroke patients in future studies to ensure that the findings are more widely applicable and representative of real-world practice. Furthermore, large independent cohorts should be used to evaluate the performance, reliability, and potential limitations of these predictive tools.

In addition, this study presents portable technologies for the detection of ICH and other stroke subtypes. Whilst some of these technologies have shown promising results, the majority are in the early stages of development. Their effectiveness, accuracy, and practicality in real-world settings remain to be fully explored through extensive testing in large cohorts of suspected stroke cases. Furthermore, the selected studies in this review reported different study designs, comparison groups, and diagnostic aims, making it challenging to draw definitive conclusions about the superiority or preference for one technology over another. To better understand the potential benefits and feasibility of using portable devices for prehospital stroke care, future research should not only focus on reporting the diagnostic performance of the devices but also consider the practical aspects of their use in prehospital settings. This includes assessing the training requirements for device use, the time needed to acquire and interpret results, and the ability of the technology to differentiate between stroke subtypes (ICH vs IS) and non-stroke diagnoses.

For peer review only - http://bmjopen.bmj.com/site/about/guidelines.xhtml

ACKNOWLEDGEMENTS

The authors wish to express their sincere gratitude to the peer reviewers of this study for their valuable input and suggestions.

FUNDING STATEMENT

This study is part of MA's PhD research, which is funded by King Saud University, Riyadh, Saudi Arabia (Grant no: NA), through the Saudi Arabian Cultural Bureau in the United Kingdom (Grant no: NA).

COMPETING INTERESTS

None declared.

AUTHOR CONTRIBUTIONS

All authors included in this paper fulfil the criteria of authorship. MA is the primary author of this work under the supervision of APJ and DAJ. DAJ conceptualised the idea for the review. MA and IA screened the studies and performed data extraction. MA conducted data analyses and wrote the manuscript with support from APJ and DAJ. The manuscript was critically reviewed and approved by all authors for final submission.

ETHICS APPROVAL

This is a scoping review; therefore, ethical approval is not required.

DATA AVAILABILITY STATEMENT

All data relevant to this study are included in the article or uploaded as supplementary information.

For peer review only - http://bmjopen.bmj.com/site/about/guidelines.xhtml

Table 1. Inclusion and exclusion criteria.

PCC	Included	Excluded
Population	• Studies of adult patients (≥16 years old) with suspected stroke.	• Studies of children (<16 years old).<br>• Studies combined ICH clinical features with other aetiologies.
Concept	• Studies reported the clinical features of suspected stroke patients, obtained during prehospital care or within 24 hours of symptoms onset upon hospital admission.<br>• Studies evaluated portable devices to detect ICH.	• Studies examined the clinical features of patients after 24 hours of symptoms onset or focused on ICH-related clinical features without comparisons to other suspected stroke cases.<br>• Studies that failed to provide information on the diagnostic performance of the technology being tested, or examined advances in conventional detection methods, or restricted their testing to phantoms or animal models.
Context	• Prehospital and in-hospital studies.	• Primary care studies.
Abbreviations: ICH, intracerebral haemorrhage; PCC, population, concept, context.		

For peer review only

For peer review only - http://bmjopen.bmj.com/site/about/guidelines.xhtml

Table 2. Summary of reported clinical features for predicting ICH in suspected stroke patients.

Category	Findings	References
Patients' general information and medical history	• Age and sex showed inconsistent predictive value for ICH.<br>• Hypertension, prior ICH, haemodialysis, alcohol consumption, and warfarin use were more frequently associated with ICH.<br>• Atrial fibrillation, coronary artery disease, dyslipidaemia, diabetes mellitus, peripheral artery disease, and previous TIA/IS were less commonly associated with ICH.<br>• Some studies found weak or no associations between the aforementioned factors or conditions and the prediction of ICH.	[25-31,33-39]
Signs and symptoms	• Headaches, vomiting, impaired consciousness, and seizures were reported, but these symptoms were not specific to ICH.<br>• Awakening with stroke symptoms was less likely to be associated with ICH.<br>• Patients with ICH exhibited more neurological deficits as measured by the NIHSS.	[25-30,32-39]
Vital signs	• Elevated blood pressure levels were more consistently observed at the onset of ICH.<br>• Arrhythmias were less frequently seen in patients with ICH.<br>• High blood glucose levels were reported in patients with ICH.	[25-29,31-35,37,38]

Abbreviations: ICH, intracerebral haemorrhage; IS, ischaemic stroke; NIHSS, National Institutes of Health Stroke Scale; TIA, transient ischaemic attack.

For peer review only - http://bmjopen.bmj.com/site/about/guidelines.xhtml

FIGURE CAPTION

Figure 1. Flowchart of study selection process (*search results for clinical features + portable devices, respectively).

For peer review only

For peer review only - http://bmjopen.bmj.com/site/about/guidelines.xhtml

REFERENCES

1. Colton K, Richards CT, Pruitt PB, et al. Early Stroke Recognition and Time-based Emergency Care Performance Metrics for Intracerebral Hemorrhage. J Stroke Cerebrovasc Dis. 2020;29(2):104552.

2. Bowry R, Parker SA, Bratina P, et al. Hemorrhage Enlargement Is More Frequent in the First 2 Hours: A Prehospital Mobile Stroke Unit Study. Stroke. 2022;53(7):2352-60.

3. Brott T, Broderick J, Kothari R, et al. Early hemorrhage growth in patients with intracerebral hemorrhage. Stroke. 1997;28(1):1-5.

4. Broderick JP, Diringer MN, Hill MD, et al. Determinants of intracerebral hemorrhage growth: an exploratory analysis. Stroke. 2007;38(3):1072-5.

5. Rodriguez-Luna D, Rodriguez-Villatoro N, Juega JM, et al. Prehospital Systolic Blood Pressure Is Related to Intracerebral Hemorrhage Volume on Admission. Stroke. 2018;49(1):204-6.

6. Song L, Chen C, Chen X, et al. INTEnsive ambulance-delivered blood pressure Reduction in hyper-ACute stroke Trial (INTERACT4): study protocol for a randomized controlled trial. Trials. 2021;22(1):885.

7. Yassi N, Zhao H, Churilov L, et al. Tranexamic acid for intracerebral haemorrhage within 2 hours of onset: protocol of a phase II randomised placebo-controlled double-blind multicentre trial. Stroke Vasc Neurol. 2022;7(2):158-65.

8. Naidech AM, Grotta J, Elm J, et al. Recombinant factor VIIa for hemorrhagic stroke treatment at earliest possible time (FASTEST): Protocol for a phase III, double-blind, randomized, placebo-controlled trial. Int J Stroke. 2021:806-9.

9. Greenberg SM, Ziai WC, Cordonnier C, et al. 2022 Guideline for the Management of Patients With Spontaneous Intracerebral Hemorrhage: A Guideline From the American Heart Association/American Stroke Association. Stroke. 2022;53(7):e282-e361.

10. Ramos-Pachón A, Rodríguez-Luna D, Martí-Fàbregas J, et al. Effect of Bypassing the Closest Stroke Center in Patients with Intracerebral Hemorrhage: A Secondary Analysis of the RACECAT Randomized Clinical Trial. JAMA Neurol. 2023;80(10):1028-36.

For peer review only - http://bmjopen.bmj.com/site/about/guidelines.xhtml

11. Nguyen TTM, van den Wijngaard IR, Bosch J, et al. Comparison of Prehospital Scales for Predicting Large Anterior Vessel Occlusion in the Ambulance Setting. JAMA Neurol. 2021;78(2):157-64.

12. Richards CT, Oostema JA, Chapman SN, et al. Prehospital Stroke Care Part 2: On-Scene Evaluation and Management by Emergency Medical Services Practitioners. Stroke. 2023;54(5):1416-25.

13. Sandercock PA, Allen CM, Corston RN, et al. Clinical diagnosis of intracranial haemorrhage using Guy's Hospital score. Br Med J (Clin Res Ed). 1985;291(6510):1675-7.

14. Poungvarin N, Viriyavejakul A, Komontri C. Siriraj stroke score and validation study to distinguish supratentorial intracerebral haemorrhage from infarction. BMJ. 1991;302(6792):1565-7.

15. Runchey S, McGee S. Does this patient have a hemorrhagic stroke? Clinical findings distinguishing hemorrhagic stroke from ischemic stroke. JAMA. 2010;303(22):2280-6.

16. Badam P, Solao V, Pai M, et al. Poor accuracy of the Siriraj and Guy's hospital stroke scores in distinguishing haemorrhagic from ischaemic stroke in a rural, tertiary care hospital. Natl Med J India. 2003;16(1):8-12.

17. Walsh KB. Non-invasive sensor technology for prehospital stroke diagnosis: Current status and future directions. Int J Stroke. 2019;14(6):592-602.

18. Lin MP, Sanossian N, Liebeskind DS. Imaging of prehospital stroke therapeutics. Expert Rev Cardiovasc Ther. 2015;13(9):1001-15.

19. Peters M, Godfrey C, Khalil H, et al. 2017 Guidance for the Conduct of JBI Scoping Reviews. 2017.

20. Peters MDJ, Marnie C, Colquhoun H, et al. Scoping reviews: reinforcing and advancing the methodology and application. Syst Rev. 2021;10(1):263.

21. Tricco AC, Lillie E, Zarin W, et al. PRISMA Extension for Scoping Reviews (PRISMA-ScR): Checklist and Explanation. Ann Intern Med. 2018;169(7):467-73.

22. Almubayyidh M, Alghamdi I, Parry-Jones AR, et al. Clinical features and novel technologies for prehospital detection of intracerebral haemorrhage: a scoping review protocol. BMJ Open. 2023;13(5):e070228.

23. Ouzzani M, Hammady H, Fedorowicz Z, et al. Rayyan-a web and mobile app for systematic reviews. Syst Rev. 2016;5(1):210.

For peer review only

For peer review only - http://bmjopen.bmj.com/site/about/guidelines.xhtml

24. Chennareddy S, Kalagara R, Smith C, et al. Portable stroke detection devices: a systematic scoping review of prehospital applications. BMC Emerg Med. 2022;22(1):111.

25. Woisetschläger C, Kittler H, Oschatz E, et al. Out-of-hospital diagnosis of cerebral infarction versus intracranial hemorrhage. Intensive Care Med. 2000;26(10):1561-5.

26. Yamashita S, Kimura K, Iguchi Y, et al. Kurashiki Prehospital Stroke Subtyping Score (KP3S) as a means of distinguishing ischemic from hemorrhagic stroke in emergency medical services. Eur Neurol. 2011;65(4):233-8.

27. Jin HQ, Wang JC, Sun YA, et al. Prehospital Identification of Stroke Subtypes in Chinese Rural Areas. Chin Med J (Engl). 2016;129(9):1041-6.

28. Uchida K, Yoshimura S, Hiyama N, et al. Clinical Prediction Rules to Classify Types of Stroke at Prehospital Stage. Stroke. 2018;49(8):1820-7.

29. Geisler F, Wesirow M, Ebinger M, et al. Probability assessment of intracerebral hemorrhage in prehospital emergency patients. Neurol Res Pract. 2021;3(1):1.

30. Chiquete E, Jiménez-Ruiz A, García-Grimshaw M, et al. Prediction of acute neurovascular syndromes with prehospital clinical features witnessed by bystanders. Neurol Sci. 2021;42(8):3217-24.

31. Gioia LC, Zewude RT, Kate MP, et al. Prehospital systolic blood pressure is higher in acute stroke compared with stroke mimics. Neurology. 2016;86(23):2146-53.

32. Irisawa T, Iwami T, Kitamura T, et al. An association between systolic blood pressure and stroke among patients with impaired consciousness in out-of-hospital emergency settings. BMC Emerg Med. 2013;13:24.

33. Andersson Hagiwara M, Wireklint Sundström B, Brink P, et al. A shorter system delay for haemorrhagic stroke than ischaemic stroke among patients who use emergency medical service. Acta Neurol Scand. 2018;137(5):523-30.

34. Puustjärvi V, Strbian D, Tiainen M, et al. Recognition of posterior circulation stroke. Acta Neurol Scand. 2015;131(6):389-93.

35. Bustamante A, Penalba A, Orset C, et al. Blood Biomarkers to Differentiate Ischemic and Hemorrhagic Strokes. Neurology. 2021;96(15):e1928-e39.

36. Montaner J, Mendioroz M, Delgado P, et al. Differentiating ischemic from hemorrhagic stroke using plasma biomarkers: the S100B/RAGE pathway. J Proteomics. 2012;75(15):4758-65.

For peer review only - http://bmjopen.bmj.com/site/about/guidelines.xhtml

37. Rozanski M, Waldschmidt C, Kunz A, et al. Glial Fibrillary Acidic Protein for Prehospital Diagnosis of Intracerebral Hemorrhage. Cerebrovasc Dis. 2017;43(1-2):76-81.

38. Bustamante A, López-Cancio E, Pich S, et al. Blood Biomarkers for the Early Diagnosis of Stroke: The Stroke-Chip Study. Stroke. 2017;48(9):2419-25.

39. Llombart V, García-Berrocoso T, Bustamante A, et al. Plasmatic retinol-binding protein 4 and glial fibrillary acidic protein as biomarkers to differentiate ischemic stroke and intracerebral hemorrhage. J Neurochem. 2016;136(2):416-24.

40. Persson M, Fhager A, Trefná HD, et al. Microwave-based stroke diagnosis making global prehospital thrombolytic treatment possible. IEEE Trans Biomed Eng. 2014;61(11):2806-17.

41. Kellner CP, Sauvageau E, Snyder KV, et al. The VITAL study and overall pooled analysis with the VIPS non-invasive stroke detection device. J Neurointerv Surg. 2018;10(11):1079-84.

42. Seidel G, Kaps M, Gerriets T. Potential and limitations of transcranial color-coded sonography in stroke patients. Stroke. 1995;26(11):2061-6.

43. Mäurer M, Shambal S, Berg D, et al. Differentiation between intracerebral hemorrhage and ischemic stroke by transcranial color-coded duplex-sonography. Stroke. 1998;29(12):2563-7.

44. Antipova D, Eadie L, Makin S, et al. The use of transcranial ultrasound and clinical assessment to diagnose ischaemic stroke due to large vessel occlusion in remote and rural areas. PLoS One. 2020;15(10):e0239653.

45. Michelson EA, Hanley D, Chabot R, et al. Identification of acute stroke using quantified brain electrical activity. Acad Emerg Med. 2015;22(1):67-72.

46. Erani F, Zolotova N, Vanderschelden B, et al. Electroencephalography Might Improve Diagnosis of Acute Stroke and Large Vessel Occlusion. Stroke. 2020;51(11):3361-5.

47. Broderick J, Connolly S, Feldmann E, et al. Guidelines for the Management of Spontaneous Intracerebral Hemorrhage in Adults. Stroke. 2007;38(6):2001-23.

48. Besson G, Robert C, Hommel M, et al. Is It Clinically Possible to Distinguish Nonhemorrhagic Infarct From Hemorrhagic Stroke? Stroke. 1995;26(7):1205-9.

For peer review only - http://bmjopen.bmj.com/site/about/guidelines.xhtml

49. Efstathiou SP, Tsioulos DI, Zacharos ID, et al. A new classification tool for clinical differentiation between haemorrhagic and ischaemic stroke. J Intern Med. 2002;252(2):121-9.
50. Massaro AR, Sacco RL, Scaff M, et al. Clinical discriminators between acute brain hemorrhage and infarction: a practical score for early patient identification. Arq Neuropsiquiatr. 2002;60(2-a):185-91.
51. Edlow JA, Caplan LR. Avoiding pitfalls in the diagnosis of subarachnoid hemorrhage. N Engl J Med. 2000;342(1):29-36.
52. Lee SE, Choi MH, Kang HJ, et al. Stepwise stroke recognition through clinical information, vital signs, and initial labs (CIVIL): Electronic health record-based observational cohort study. PLoS One. 2020;15(4):e0231113.
53. Farooq S, Shkirkova K, Villablanca P, et al. National Institutes of Health Stroke Scale Correlates Well with Initial Intracerebral Hemorrhage Volume. J Stroke Cerebrovasc Dis. 2022;31(4):106348.
54. Larsen K, Hov MR, Sandset EC. Prehospital stroke scales-the need for a gold standard in the field. Acta Neurol Scand. 2022;145(3):263-4.
55. Larsen K, Jæger HS, Hov MR, et al. Streamlining Acute Stroke Care by Introducing National Institutes of Health Stroke Scale in the Emergency Medical Services: A Prospective Cohort Study. Stroke. 2022;53(6):2050-7.
56. Ohwaki K, Yano E, Nagashima H, et al. Blood pressure management in acute intracerebral hemorrhage: relationship between elevated blood pressure and hematoma enlargement. Stroke. 2004;35(6):1364-7.
57. Shkirkova K, Saver JL, Starkman S, et al. Frequency, Predictors, and Outcomes of Prehospital and Early Postarrival Neurological Deterioration in Acute Stroke: Exploratory Analysis of the FAST-MAG Randomized Clinical Trial. JAMA Neurol. 2018;75(11):1364-74.
58. Tsou YJ, Lan KP, Fan JS. Relationship Between Changes in Prehospital Blood Pressure and Early Neurological Deterioration in Spontaneous Intracerebral Hemorrhage. Adv Emerg Nurs J. 2019;41(2):163-71.
59. Moon JS, Janjua N, Ahmed S, et al. Prehospital neurologic deterioration in patients with intracerebral hemorrhage. Crit Care Med. 2008;36(1):172-5.

For peer review only

For peer review only - http://bmjopen.bmj.com/site/about/guidelines.xhtml

60. Gomes JA, Ahrens CL, Hussain MS, et al. Prehospital reversal of warfarin-related coagulopathy in intracerebral hemorrhage in a mobile stroke treatment unit. Stroke. 2015;46(5):e118-20.

61. Cooley SR, Zhao H, Campbell BCV, et al. Mobile Stroke Units Facilitate Prehospital Management of Intracerebral Hemorrhage. Stroke. 2021;52(10):3163-6.

62. Walter S, Grunwald IQ, Helwig SA, et al. Mobile Stroke Units - Cost-Effective or Just an Expensive Hype? Curr Atheroscler Rep. 2018;20(10):49.

63. Pulvers JN, Watson JDG. If Time Is Brain Where Is the Improvement in Prehospital Time after Stroke? Front Neurol. 2017;8:617.

64. Leon-Carrion J, Dominguez-Roldan JM, Leon-Dominguez U, et al. The Infrascanner, a handheld device for screening in situ for the presence of brain haematomas. Brain Inj. 2010;24(10):1193-201.

65. Kontojannis V, Hostettler I, Brogan RJ, et al. Detection of intracranial hematomas in the emergency department using near infrared spectroscopy. Brain Inj. 2019;33(7):875-83.

66. Mattila OS, Ashton NJ, Blennow K, et al. Ultra-Early Differential Diagnosis of Acute Cerebral Ischemia and Hemorrhagic Stroke by Measuring the Prehospital Release Rate of GFAP. Clin Chem. 2021;67(10):1361-72.

67. Dagonnier M, Donnan GA, Davis SM, et al. Acute Stroke Biomarkers: Are We There Yet? Front Neurol. 2021;12:619721.

For peer review only

For peer review only - http://bmjopen.bmj.com/site/about/guidelines.xhtml

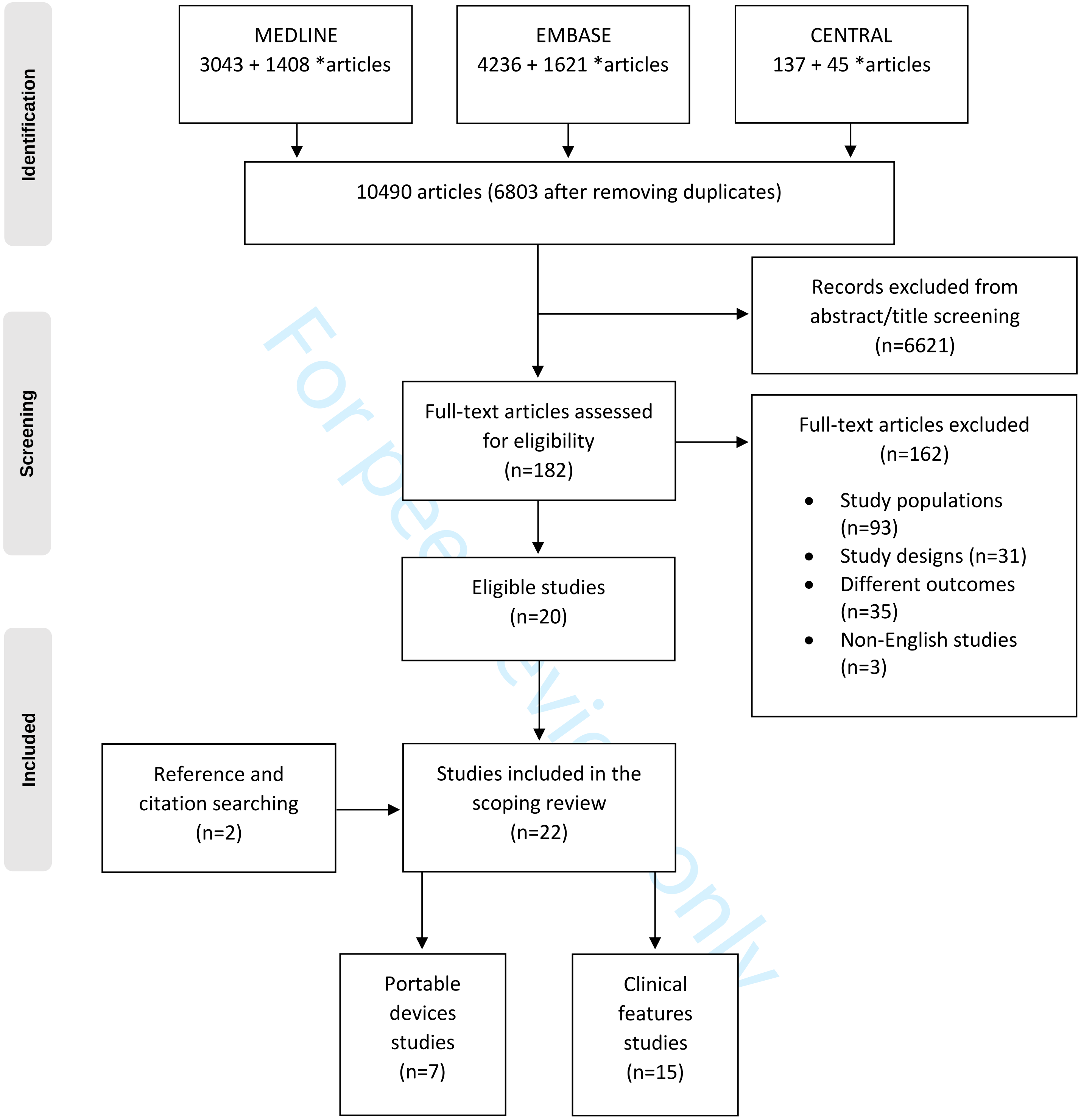
Identification
Screening
Included
MEDLINE
3043 + 1408 *articles
EMBASE
4236 + 1621 *articles
CENTRAL
137 + 45 *articles
10490 articles (6803 after removing duplicates)
Records excluded from abstract/title screening (n=6621)
Full-text articles assessed for eligibility (n=182)
Full-text articles excluded (n=162)
Study populations (n=93)
Study designs (n=31)
Different outcomes (n=35)
Non-English studies (n=3)
Eligible studies (n=20)
Reference and citation searching (n=2)
Studies included in the scoping review (n=22)
Portable devices studies (n=7)
Clinical features studies (n=15)

Appendix 1. Preferred Reporting Items for Systematic reviews and Meta-Analyses extension for Scoping Reviews (PRISMA-ScR) Checklist

SECTION	ITEM	PRISMA-ScR CHECKLIST ITEM	REPORTED ON PAGE #
**TITLE**			
Title	1	Identify the report as a scoping review.	1
**ABSTRACT**			
Structured summary	2	Provide a structured summary that includes (as applicable): background, objectives, eligibility criteria, sources of evidence, charting methods, results, and conclusions that relate to the review questions and objectives.	2–3
**INTRODUCTION**			
Rationale	3	Describe the rationale for the review in the context of what is already known. Explain why the review questions/objectives lend themselves to a scoping review approach.	4–5
Objectives	4	Provide an explicit statement of the questions and objectives being addressed with reference to their key elements (e.g., population or participants, concepts, and context) or other relevant key elements used to conceptualize the review questions and/or objectives.	4–6
**METHODS**			
Protocol and registration	5	Indicate whether a review protocol exists; state if and where it can be accessed (e.g., a Web address); and if available, provide registration information, including the registration number.	5
Eligibility criteria	6	Specify characteristics of the sources of evidence used as eligibility criteria (e.g., years considered, language, and publication status), and provide a rationale.	6
Information sources*	7	Describe all information sources in the search (e.g., databases with dates of coverage and contact with authors to identify additional sources), as well as the date the most recent search was executed.	6
Search	8	Present the full electronic search strategy for at least 1 database, including any limits used, such that it could be repeated.	Appendix 2
Selection of sources of evidence†	9	State the process for selecting sources of evidence (i.e., screening and eligibility) included in the scoping review.	6
Data charting process‡	10	Describe the methods of charting data from the included sources of evidence (e.g., calibrated forms or forms that have been tested by the team before their use, and whether data charting was done independently or in duplicate) and any processes for obtaining and confirming data from investigators.	7
Data items	11	List and define all variables for which data were sought and any assumptions and simplifications made.	Appendix 2
Critical appraisal of individual sources of evidence§	12	If done, provide a rationale for conducting a critical appraisal of included sources of evidence; describe the methods used and how this information was used in any data synthesis (if appropriate).	N/A
Synthesis of results	13	Describe the methods of handling and summarizing the data that were charted.	7

St. Michael's
Inspired Care. Inspiring Science.

For peer review only - http://bmjopen.bmj.com/site/about/guidelines.xhtml

SECTION	ITEM	PRISMA-ScR CHECKLIST ITEM	REPORTED ON PAGE #
**RESULTS**			
Selection of sources of evidence	14	Give numbers of sources of evidence screened, assessed for eligibility, and included in the review, with reasons for exclusions at each stage, ideally using a flow diagram.	7, Figure 1
Characteristics of sources of evidence	15	For each source of evidence, present characteristics for which data were charted and provide the citations.	7–8
Critical appraisal within sources of evidence	16	If done, present data on critical appraisal of included sources of evidence (see item 12).	N/A
Results of individual sources of evidence	17	For each included source of evidence, present the relevant data that were charted that relate to the review questions and objectives.	Supplementary Tables 1 and 2
Synthesis of results	18	Summarize and/or present the charting results as they relate to the review questions and objectives.	7–13, 22
**DISCUSSION**			
Summary of evidence	19	Summarize the main results (including an overview of concepts, themes, and types of evidence available), link to the review questions and objectives, and consider the relevance to key groups.	13–18
Limitations	20	Discuss the limitations of the scoping review process.	18
Conclusions	21	Provide a general interpretation of the results with respect to the review questions and objectives, as well as potential implications and/or next steps.	18–19
**FUNDING**			
Funding	22	Describe sources of funding for the included sources of evidence, as well as sources of funding for the scoping review. Describe the role of the funders of the scoping review.	19

JBI = Joanna Briggs Institute; PRISMA-ScR = Preferred Reporting Items for Systematic reviews and Meta-Analyses extension for Scoping Reviews.
* Where *sources of evidence* (see second footnote) are compiled from, such as bibliographic databases, social media platforms, and Web sites.
† A more inclusive/heterogeneous term used to account for the different types of evidence or data sources (e.g., quantitative and/or qualitative research, expert opinion, and policy documents) that may be eligible in a scoping review as opposed to only studies. This is not to be confused with *information sources* (see first footnote).
‡ The frameworks by Arksey and O'Malley (6) and Levac and colleagues (7) and the JBI guidance (4, 5) refer to the process of data extraction in a scoping review as data charting.
§ The process of systematically examining research evidence to assess its validity, results, and relevance before using it to inform a decision. This term is used for items 12 and 19 instead of "risk of bias" (which is more applicable to systematic reviews of interventions) to include and acknowledge the various sources of evidence that may be used in a scoping review (e.g., quantitative and/or qualitative research, expert opinion, and policy document).

From: Tricco AC, Lillie E, Zarin W, O'Brien KK, Colquhoun H, Levac D, et al. PRISMA Extension for Scoping Reviews (PRISMAScR): Checklist and Explanation. Ann Intern Med. 2018;169:467–473. doi: 10.7326/M18-0850.

For peer review only

Appendix 2. Search strategies for the scoping review

1- The search strategy for clinical features

Database searched: MEDLINE (Ovid)

#	Query
1	exp Brain Ischemia/ or exp Stroke/ or exp Hemorrhagic Stroke/ or exp Cerebral Hemorrhage/ or exp Intracranial Hemorrhages/
2	Intracerebral hemorrhage.mp.
3	1 or 2
4	exp cerebrovascular accident/di
5	exp stroke/di
6	4 or 5
7	(recogni$ or identi$ or strati$ or different$ or probabilit$).mp.
8	3 and 7
9	6 or 8
10	exp Emergency Medical Services/ or Paramedic*.mp. or ambulance.mp. or triage.mp. or prehospital.mp. or pre hospital.mp. or EMS.mp. or emergency medical service*.mp.
11	Air Ambulances/
12	Emergency Service, Hospital/
13	Emergency Medicine/
14	Clinical feature.mp.
15	Stroke Score.mp.
16	Clinical differentiation.mp.
17	10 or 11 or 12 or 13 or 14 or 15 or 16
18	9 and 17
19	limit 18 to english language

For peer review only - http://bmjopen.bmj.com/site/about/guidelines.xhtml

Database searched: EMBASE (Ovid)

#	Query
1	exp Brain Ischemia/ or exp Stroke/ or exp Hemorrhagic Stroke/ or exp Cerebral Hemorrhage/ or exp Intracranial Hemorrhages/
2	Intracerebral hemorrhage.mp.
3	1 or 2
4	exp cerebrovascular accident/di [Diagnosis]
5	exp stroke/di
6	4 or 5
7	(recogni$ or identi$ or strati$ or distinguish$ or probabilit$).mp.
8	3 and 7
9	6 or 8
10	exp Emergency Medical Services/ or Paramedic*.mp. or ambulance.mp. or triage.mp. or prehospital.mp. or pre hospital.mp. or EMS.mp. or emergency medical service*.mp. or out-of-hospital.mp.
11	Air Ambulances/
12	Emergency Service, Hospital/
13	Stroke Score.mp.
14	Clinical differentiation.mp.
15	10 or 11 or 12 or 13 or 14
16	9 and 15
17	limit 16 to (human and english language)

For peer review only

Database searched: CENTRAL (Ovid)

#	Query
1	exp Brain Ischemia/ or exp Stroke/ or exp Hemorrhagic Stroke/ or exp Cerebral Hemorrhage/ or exp Intracranial Hemorrhages/
2	Intracerebral hemorrhage.mp.
3	1 or 2
4	exp cerebrovascular accident/di
5	exp stroke/di
6	4 or 5
7	(recogni$ or identi$ or strati$ or different$ or probabilit$).mp.
8	3 and 7
9	6 or 8
10	exp Emergency Medical Services/ or Paramedic*.mp. or ambulance.mp. or triage.mp. or prehospital.mp. or pre hospital.mp. or EMS.mp. or emergency medical service*.mp.
11	Air Ambulances/
12	Emergency Service, Hospital/
13	Emergency Medicine/
14	Clinical feature.mp.
15	Stroke Score.mp.
16	Clinical differentiation.mp.
17	10 or 11 or 12 or 13 or 14 or 15 or 16
18	9 and 17
19	limit 18 to english language

For peer review only

2- The search strategy for portable devices

Database searched: MEDLINE (Ovid)

#	Query
1	exp Brain Ischemia/ or exp Stroke/ or exp Hemorrhagic Stroke/ or exp Cerebral Hemorrhage/ or exp Intracranial Hemorrhages/
2	Intracerebral hemorrhage.mp.
3	1 or 2
4	(recogni$ or identi$ or strati$ or diagnos$ or detect$).mp.
5	exp Emergency Medical Services/ or Paramedic*.mp. or ambulance.mp. or triage.mp. or prehospital.mp. or pre hospital.mp. or EMS.mp. or emergency medical service*.mp.
6	Air Ambulances/
7	Emergency Service, Hospital/
8	(technology or spectroscopy or ultrasound or diagnostic imaging or imaging or transcranial doppler or radiofrequency or microwave or electroencephalography or device).mp.
9	5 or 6 or 7
10	3 and 4 and 8 and 9
11	limit 10 to (english language and humans)

For peer review only

Database searched: EMBASE (Ovid)

#	Query
1	exp Brain Ischemia/ or exp Stroke/ or exp Hemorrhagic Stroke/ or exp Cerebral Hemorrhage/ or exp Intracranial Hemorrhages/
2	Intracerebral hemorrhage.mp.
3	1 or 2
4	(recogni$ or identi$ or strati$ or diagnos$ or detect$).mp.
5	exp Emergency Medical Services/ or Paramedic*.mp. or ambulance.mp. or triage.mp. or prehospital.mp. or pre hospital.mp. or EMS.mp. or emergency medical service*.mp.
6	Air Ambulances/
7	Emergency Service, Hospital/
8	(technology or spectroscopy or ultrasound or diagnostic imaging or imaging or transcranial doppler or radiofrequency or microwave or electroencephalography or device).mp.
9	5 or 6 or 7
10	3 and 4 and 8 and 9
11	limit 10 to (english language and humans)

For peer review only

Database searched: CENTRAL (Ovid)

#	Query
1	exp Brain Ischemia/ or exp Stroke/ or exp Hemorrhagic Stroke/ or exp Cerebral Hemorrhage/ or exp Intracranial Hemorrhages/
2	Intracerebral hemorrhage.mp.
3	1 or 2
4	(recogni$ or identi$ or strati$ or diagnos$ or detect$).mp.
5	exp Emergency Medical Services/ or Paramedic*.mp. or ambulance.mp. or triage.mp. or prehospital.mp. or pre hospital.mp. or EMS.mp. or emergency medical service*.mp.
6	Air Ambulances/
7	Emergency Service, Hospital/
8	(technology or spectroscopy or ultrasound or diagnostic imaging or imaging or transcranial doppler or radiofrequency or microwave or electroencephalography or device).mp.
9	5 or 6 or 7
10	3 and 4 and 8 and 9
11	limit 10 to (english language and humans)

For peer review only

Supplementary Table 1. Characteristics of the included studies for clinical features.

Included study, year and country	Design	Setting	Study population	Statistical method	Age (Years)	Onset-to-evaluation	Method of confirmation	Clinical features collected			Findings relevant to review
								Past medical history	Signs and symptoms	Vital signs	
Woisetschläger [25], 2000; Austria	Retrospective -prospective study	Prehospital and in-hospital	ICH, IS<br>ICH: 118<br>IS: 106	Uni- and multi-variable regression	ICH (mean): 58<br>IS (mean): 65	NR (Evaluated in prehospital)	CT scan	• AF<br>• Aspirin use<br>• Dicoumarin use<br>• DM<br>• HTN<br>• Hyperlipidaemia<br>• Prior MI<br>• Smoking	• Aphasia<br>• Headache<br>• Hemisymptoms<br>• Impaired LOC<br>• Seizures<br>• Syncope<br>• Vomiting	• SBP<br>• DBP<br>• ECG	• ICH patients were younger than IS patients.<br>• A history of HTN, DM, and MI was more commonly observed in IS patients.<br>• Impaired LOC was predictive of ICH.<br>• Unilateral weakness or sensory loss was observed more frequently in IS patients.
Yamashita [26], 2011; Japan	Retrospective study	Prehospital and in-hospital	ICH, IS<br>ICH: 100<br>IS: 127	Uni- and multi-variable regression	ICH (median): 68<br>IS (median): 74	Within 6 h of onset	CT or MRI scan	• ACS<br>• AF<br>• Antithrombotics use<br>• DM<br>• Haemodialysis<br>• HTN<br>• Hyperlipidaemia<br>• Prior TIA, IS, and ICH<br>• Smoking	• Headache<br>• Neurological deficits using NIHSS items<br>• Seizures<br>• Vomiting	• SBP<br>• DBP<br>• ECG	• ICH patients were younger than IS patients.<br>• Patients with ICH were more likely to have a history of ICH and haemodialysis, while patients with IS had a higher likelihood of a history of ACS, AF, antithrombotic usage, and hyperlipidaemia.<br>• Headache, vomiting, impaired LOC, and seizures were more frequently observed in the ICH group.<br>• The NIHSS median score was higher in the ICH group (16 vs 9).<br>• The median BP was higher in the ICH group, including both SBP (190 vs 164 mm Hg) and DBP (102 vs 90 mm Hg).
Jin [27], 2016; China	Prospective study	Prehospital and in-hospital	ICH, IS, SAH, non-stroke<br>ICH: 689<br>IS: 797<br>SAH: 109<br>Non-stroke: 394	Uni- and multi-variable regression	All patients (mean): 66	NR (Evaluated in prehospital)	CT or MRI scan	• AF<br>• DM<br>• HTN	• Vomiting	• SBP<br>• DBP<br>• ECG<br>• HR	• The comparison was between two groups: ICH vs IS/TIA.<br>• Age <65 years was found to be associated with the ICH group.<br>• IS/TIA patients were more likely to have a history of AF and DM.<br>• Vomiting was a common presenting symptom in the ICH group.<br>• SBP ≥180 mm Hg and DBP ≥100 mm Hg were more frequently observed in ICH patients, while arrhythmia was a common finding in the IS/TIA group.

For peer review only - http://bmjopen.bmj.com/site/about/guidelines.xhtml

Uchida [28], 2018; Japan	Prospective study	Prehospital and in-hospital	Any stroke, ICH, LVO, SAH, non-stroke<br><br>DC: 1229<br>Any stroke: 203<br>ICH: 169<br>LVO: 104<br>SAH: 57<br>Non-stroke: 696<br><br>VC: 1007<br>Any stroke: 253<br>ICH: 183<br>LVO: 131<br>SAH: 50<br>Non-stroke: 390	Uni- and multi-variable regression	DC (median): 72<br><br>VC (median): 75	NR (Evaluated in prehospital)	CT or MRI scan	• Antiplatelet use<br>• DOAC use<br>• Prior IS, ICH, and SAH<br>• Smoking<br>• Warfarin use	• Anisocoria<br>• Aphasia<br>• Conjugate deviation<br>• Convulsion<br>• Dizziness<br>• Dysarthria<br>• Facial palsy<br>• Headache<br>• Impaired LOC<br>• Nausea or vomiting<br>• Numbness<br>• Paralysis of lower limbs<br>• Paralysis of upper limbs<br>• Sudden onset<br>• Symptoms improved after onset<br>• Symptoms progressed after onset<br>• Unilateral spatial neglect	• SBP<br>• DBP<br>• ECG	• Patients with ICH were less likely to have a history of IS.<br>• Improvement following the onset of symptoms was associated with a decreased probability of ICH.<br>• Headache, dysarthria, paralysis of the upper limbs, conjugate deviation, and impaired LOC were common presentations of ICH. However, most of these symptoms were not specific.<br>• SBP ≥165 mm Hg and DBP ≥95 mm Hg were more frequently observed in ICH patients, while the presence of arrhythmia was associated with the absence of ICH.
Geisler [29], 2021; Germany	Retrospective study	Prehospital and in-hospital	ICH, IS, TIA, SM<br><br>DC: 416<br>ICH: 32<br>IS: 224<br>TIA: 41<br>SM: 119<br><br>VC: 285<br>ICH: 33<br>IS: 252	Uni- and multi-variable regression	DC (mean):<br>ICH: 71.5<br>IS: 74.9<br><br>VC (mean):<br>ICH: 73.3<br>IS: 73.9	≤4 h or unknown	CT scanner in MSU	• AF<br>• HTN	• Neurological deficits using NIHSS items<br>• Seizures	• SBP<br>• DBP<br>• MAP	• In the derivation set (ICH vs IS), ICH patients were more likely to be male.<br>• AF was found less frequently in the derivation set for the ICH group.<br>• ICH patients had higher median NIHSS scores in both sets (≥15).<br>• A greater number of ICH patients showed a decrease in LOC.<br>• The majority of seizures were found in the SM group.<br>• A higher proportion of ICH patients exhibited SBP ≥180 mm Hg, DBP ≥110 mm Hg, and MAP ≥130 mm Hg.

For peer review only - http://bmjopen.bmj.com/site/about/guidelines.xhtml

Chiquete [30], 2021; Mexico	Prospective study	Prehospital and in-hospital	ICH, IS, SAH<br><br>ICH: 107<br>IS: 209<br>SAH: 53	Uni- and multi-variable regression	ICH (median): 60<br>IS (median): 68<br>SAH (median): 62	≤6 h in most cases	CT or MRI scan	• AF<br>• DM<br>• HTN<br>• Obesity<br>• Previous stroke	• Focal motor deficit<br>• Headache<br>• Impaired LOC<br>• Language or speech disorder<br>• Vomiting	NR	• A combination of focal motor deficit and a history of HTN performed the best in predicting ICH patients among different stroke subtypes.
Gioia [31], 2016; Canada	Retrospective study	Prehospital and in-hospital	ICH, IS, TIA, SM<br><br>ICH: 51<br>IS: 367<br>TIA: 117<br>SM: 416	Uni-variable	ICH (mean): 73.1<br>IS (mean): 73.3<br>TIA (mean): 72.4<br>SM (mean): 66.9	Median time: 75 min	Brain imaging	• AF<br>• CAD<br>• CHF<br>• DM type 2<br>• Dyslipidaemia<br>• HTN<br>• Prior stroke, ICH, and TIA	NR	• SBP<br>• DBP<br>• MAP	• SM patients were younger than patients with IS/TIA/ICH.<br>• In comparison to the other groups, ICH patients had higher rates of previous ICH.<br>• ICH patients had a higher mean prehospital SBP (172.3 ± 31.7 mm Hg) compared to IS (154.9 ± 26.5 mm Hg), TIA (153.3 ± 23.1 mm Hg), and SM (145.4 ± 25.4 mm Hg).<br>• ICH patients also had a higher mean prehospital DBP (95.8 ± 22.1 mm Hg) compared to IS (87.7 ± 17.1 mm Hg), TIA (86.6 ± 15.9 mm Hg), and SM (84.7 ± 16.8 mm Hg).<br>• The mean prehospital MAP of the ICH group was higher (122.0 ± 20.2 mm Hg) compared to IS (110.2 ± 18.9 mm Hg), TIA (108.6 ± 16.6 mm Hg), and SM (105.1 ± 18.0 mm Hg).<br>• During EMS transport, the ICH group maintained the highest mean SBP.

For peer review only

For peer review only - http://bmjopen.bmj.com/site/about/guidelines.xhtml

Irisawa [32], 2013; Japan	Retrospective study	Prehospital and in-hospital	ICH, IS, SAH, non-stroke<br><br>ICH: 6699<br>IS: 24,754<br>SAH: 1631<br>Non-stroke: 73,622	Uni- and multi-variable regression	All patients (mean): 63.1	NR (Evaluated in prehospital)	NR	NR	• Impaired LOC	• SBP	• Among patients with stroke with impaired LOC, elevated SBP (≥200 mm Hg) in the prehospital setting was strongly associated with a final diagnosis of ICH.
Andersson Hagiwara [33], 2018; Sweden	Retrospective study	Prehospital and in-hospital	ICH, IS<br><br>ICH: 172<br>IS: 1164	Uni-variable	ICH (mean, median): 74, 78<br>IS (mean, median): 77, 78	<24 h from symptoms onset	CT scan	• AF<br>• Angina pectoris<br>• CHF<br>• DM<br>• HTN<br>• Intermittent claudication<br>• Malignancy<br>• Prior amaurosis fugax<br>• Prior MI<br>• Prior stroke and TIA<br>• Sleep apnoea	Prehospital assessment:<br>• Arm weakness<br>• Facial droop<br>• Impaired LOC<br>• Leg weakness<br>• Numbness<br>• Speech disturbances<br><br>In-hospital assessment:<br>• Aphasia/ dysphasia<br>• Compromised balance<br>• Double vision<br>• Dysphagia<br>• Facial droop<br>• Headache<br>• Hemiparesis/ plegia<br>• Impaired LOC<br>• Numbness<br>• Syncope<br>• Vertigo	• SBP<br>• DBP<br>• ECG<br>• HR<br>• SPO_2	• ICH patients were slightly younger than patients with IS.<br>• In terms of past medical history, a history of previous TIA was more common in the IS group.<br>• Patients with ICH woke up with symptoms less frequently than IS patients.<br>• Leg weakness and decreased LOC were more frequently observed in ICH patients in the prehospital setting.<br>• During the in-hospital assessment, patients with ICH tended to present more often with headaches, syncope, and decreased LOC.<br>• Both SBP >200 mm Hg, and DBP >90 mm Hg were more frequently observed in ICH patients in both prehospital and in-hospital settings.

For peer review only - http://bmjopen.bmj.com/site/about/guidelines.xhtml

Puustjärvi [34], 2015; Finland	Retrospective-prospective study	In-hospital	PC-ICH, PC-IS<br><br>PC-ICH: 363<br>PC-IS: 190	Uni- and multi-variable regression	ICH (median): 68<br>IS (median): 65	NR (Evaluated in prehospital)	CT or MRI scan	• AF<br>• Antiplatelet use<br>• CAD<br>• CHF<br>• DM<br>• Dyslipidaemia<br>• HTN<br>• Warfarin use	• Deterioration<br>• Headache<br>• Neurological deficits using NIHSS items<br>• Seizures<br>• Sudden onset<br>• Vomiting	• SBP<br>• DBP<br>• GCS<br>• BGL	• Patients with PC-ICH were older than those with PC-IS.<br>• PC-ICH patients were more likely to have a history of HTN and warfarin use, and less likely to have a history of AF or dyslipidaemia than PC-IS patients.<br>• Headache and vomiting at the onset were more frequently observed in the PC-ICH group.<br>• Upon arrival at the ER, PC-ICH patients had higher median BP (177/92 vs 153/86 mm Hg) as well as higher median BGL (7.4 vs 6.8 mmol/l) than PC-IS patients.
Bustamante [35], 2021; Spain	Prospective study	In-hospital	ICH, IS<br><br>ICH: 35<br>IS: 154	Uni- and multi-variable regression	ICH (median): 82<br>IS (median): 81	Within 4.5 h after symptoms onset	CT scan	• AF<br>• Alcohol intake<br>• CAD<br>• DM<br>• Dyslipidaemia<br>• HTN<br>• PAD<br>• Previous stroke<br>• Smoking	• Neurological deficits using NIHSS items	• SBP<br>• DBP<br>• BGL	• Patients with ICH tended to be more frequently male than IS patients.<br>• Patients with IS had higher rates of AF compared to ICH patients.<br>• ICH patients tended to present with a more severe stroke than patients with IS (median NIHSS 17 vs 13).<br>• The median admission BP of ICH patients was higher than that of IS patients (160/84 vs 149/77 mm Hg).
Montaner [36], 2012; Spain	Cross-sectional study	In-hospital	ICH, IS<br><br>ICH: 139<br>IS: 776	Uni- and multi-variable regression	ICH (mean): 70.35<br>IS (mean): 73.05	Within the first 24 h after symptoms onset	CT scan	• AF<br>• Alcohol intake<br>• CAD<br>• DM<br>• Dyslipidaemia<br>• Embolic cardiopathy<br>• HTN<br>• PAD<br>• Prior IS and ICH<br>• Smoking	• Neurological deficits using NIHSS items	NR	• Patients with ICH were younger and more likely to be male than IS patients.<br>• ICH patients had a higher history of ICH and alcohol use, but a lower history of dyslipidaemia, PAD, CAD, and AF, which were more prevalent among IS patients.<br>• Patients with ICH experienced a more severe stroke than those with IS (median NIHSS 11 vs 6).

For peer review only - http://bmjopen.bmj.com/site/about/guidelines.xhtml

Rozanski [37], 2017; Germany	Prospective study	Prehospital and in-hospital	ICH, IS<br><br>ICH: 25<br>IS: 49	Uni-variable	ICH (mean): 69.4<br>IS (mean): 75.3	Median time: 63 min	CT scanner in MSU	• AF<br>• DM<br>• Dyslipidaemia<br>• HTN<br>• Smoking	• Neurological deficits using NIHSS items	• SBP	• Patients with ICH were younger than those with IS.<br>• Hypercholesterolaemia (dyslipidaemia) was less common among ICH patients compared to IS patients.<br>• Patients with ICH presented with more severe strokes than IS patients (median NIHSS 15 vs 6).<br>• ICH patients had a higher mean SBP than IS patients (204 vs 162 mm Hg).
Bustamante [38], 2017; Spain	Prospective study	In-hospital	ICH, IS<br><br>ICH: 174<br>IS: 941	Uni- and multi-variable regression	ICH (median): 70<br>IS (median): 76	<6 h from symptoms onset	Brain imaging	• AF<br>• Alcohol intake<br>• CAD<br>• DM<br>• Dyslipidaemia<br>• HTN<br>• Previous stroke<br>• Smoking	• Neurological deficits using NIHSS items	• SBP<br>• DBP<br>• BGL	• ICH patients were younger and more often male than IS patients.<br>• Patients with ICH were more likely to have a history of HTN and less likely to have a history of AF or CAD than IS patients.<br>• ICH patients presented with more severe neurological deficits compared to patients with IS (median NIHSS 12 vs 7).<br>• Patients with ICH had higher median SBP (174 vs 155 mm Hg), DBP (92 vs 82 mm Hg), and BGL (7.1 vs 6.7 mmol/L*) than patients with IS.
Llombart [39], 2016; Spain	Retrospective study	In-hospital	ICH, IS<br><br>ICH: 28<br>IS: 38	Uni- and multi-variable regression	ICH (mean): 76.03<br>IS (mean): 72.28	Within 6 h from the onset of symptoms	CT scan	• AF<br>• CAD<br>• DM<br>• Dyslipidaemia<br>• HTN<br>• Previous stroke<br>• Smoking	• Neurological deficits using NIHSS items	NR	• A history of HTN and CAD was more common among IS patients than ICH patients.

*Converted from mg/dL to mmol/L by dividing by 18.

Abbreviations: ACS, acute coronary syndrome; AF, atrial fibrillation; BGL, blood glucose level; BP, blood pressure; CAD, coronary artery disease; CHF, congestive heart failure; CT, computed tomography; DBP, diastolic blood pressure; DC, derivation cohort; DM, diabetes mellitus; DOAC, direct oral anticoagulant; ECG, electrocardiogram; EMS, emergency medical services; ER, emergency room; GCS, Glasgow Coma Scale; HR, heart rate; HTN, hypertension; ICH, intracerebral haemorrhage; IS, ischaemic stroke; LOC, level of consciousness; LVO, large vessel occlusion; MAP, mean arterial pressure; MI, myocardial infarction; MRI, magnetic resonance imaging; MSU, mobile stroke unit; NIHSS, National Institutes of Health Stroke Scale; NR, not reported; PAD, peripheral artery disease; PC-ICH, posterior circulation intracerebral haemorrhage; PC-IS, posterior circulation ischaemic stroke; SAH, subarachnoid haemorrhage; SBP, systolic blood pressure; SM, stroke mimic; SPO_2, peripheral oxygen saturation; TIA, transient ischaemic attack; VC, validation cohort.

For peer review only - http://bmjopen.bmj.com/site/about/guidelines.xhtml

Supplementary Table 2. Characteristics of the included studies for portable technologies.

Included study, year and country	Design	Technology and device employed	Study population	Sample size (Number)	Time to results	Comparator	User expertise	Diagnostic accuracy					Potential limitations
								SEN (95% CI)	SPE (95% CI)	AUC (95% CI)	PPV (95% CI)	NPV (95% CI)	
Persson [40], 2014; Sweden	Proof-of-concept study	Microwave<br><br>2 prototype systems	Patients with stroke, healthy controls	Study 1: 20<br><br>ICH: 9<br><br>IS: 11<br><br>Study 2: 90<br><br>ICH: 10<br><br>IS: 15<br><br>Healthy controls: 65	NR	CT scan	Staff members from the engineering, neurophysiology, and nursing departments	The first clinical study (study 1): ICH vs IS					• The development of brain oedema after IS may limit the diagnostic accuracy of this imaging technique.
								NR	NR	0.88	NR	NR	
								The second clinical study (study 2): ICH vs IS					
								NR	NR	0.85	NR	NR	
								The second clinical study (study 2): ICH vs healthy controls					
								NR	NR	0.87	NR	NR	
Kellner [41], 2018; USA	Pilot study	VIPS<br><br>Cerebrotech Visor	Suspected stroke patients, healthy controls	248<br><br>Severe strokes: 57 (2 with ICH ≥60 ml)<br><br>Minor strokes: 26 (10 with ICH <60 ml)<br><br>Healthy controls: 79<br><br>Other subjects: 86	30 sec	Brain imaging and prehospital stroke scales	Trained staff	Severe strokes vs minor strokes					• Metallic implants (intracranial or extracranial) may interfere with the device's ability to provide accurate measurements.<br>• No evidence was provided that VIPS could distinguish ICH from other conditions (e.g., IS).
								93% (83–98)	92% (75–99)	0.93 (0.85–0.97)	96% (88–99)	86% (70–94)	
								Severe strokes vs all subjects enrolled					
								93% (83–98)	87% (81–92)	0.93 (0.89–0.96)	70% (61–77)	98% (94–99)	

For peer review only

For peer review only - http://bmjopen.bmj.com/site/about/guidelines.xhtml

Seidel [42], 1995; Germany	Prospective study	Ultrasound<br><br>HP SONOS 1000	Suspected stroke patients	84<br><br>ICH: 15<br><br>IS/TIA (in MCA): 48<br><br>IS/TIA (in PCA): 4<br><br>Other subjects: 17	NR	CT scan	NR	TCCS as a first diagnostic modality for ICH					• The detection of small ICH (<1×1cm) is limited.<br>• Brain tumours like gliomas, and haemorrhagic infarcts could be misdiagnosed as a spontaneous ICH.<br>• A total of 20% (17/84) of the enrolled patients could not be assessed due to inadequate bone windows.
								88%	96%	NR	88%	96%	
Mäurer [43], 1998; Germany	Prospective study	Ultrasound<br><br>Siemens Sonoline Ellegra or Acuson 128 XP/4	Suspected stroke patients	151<br><br>ICH: 60<br><br>IS: 67<br><br>Non-stroke: 24	NR	CT scan	Neuroradiologist	ICH vs IS					• The assessment of the frontal and parasagittal brain regions is challenging when the acoustic bone window is narrow.<br>• Increased echo density of the white matter in patients with cerebral microangiopathy may mimic the sonographic pattern of ICH.
								94%	95%	NR	91%	95%	

For peer review only

Antipova [44], 2020; UK	Exploratory study	Ultrasound<br><br>SonoSite M-Turbo, Philips Sparq, or Philips CX50 ultrasound	Suspected stroke patients	107<br><br>ICH: 18<br><br>IS: 63<br><br>TIA: 13<br><br>SM: 13	20 min	CT scan	Expert sonographer	ICH vs IS/TIA/SM based on TCCS and clinical examination					• Inadequate acoustic windows (18/107) and user dependency may hinder the potential application.
								63%	99%	0.912 (0.829–0.996)	91%	92%	
Michelson [45], 2015; USA	Retrospective study	EEG<br><br>BrainScope	Patients with stroke, SM controls	183<br><br>HS: 17<br><br>IS: 31<br><br>SM controls: 135	10 min	CT or MRI scan	Expert EEG technologist	Classification result of HS					• Previous TIAs/strokes and the use of neuroactive medications could alter EEG findings.<br>• The device was not demonstrated to be capable of distinguishing between stroke subtypes.
								94.1%	50.4% (43.0–58.7)	NR	NR	NR	
Erani [46], 2020; USA	Proof-of-concept study	EEG<br><br>Quick-20	Suspected stroke patients	100<br><br>ICH: 7<br><br>IS: 43<br><br>TIA: 13<br><br>Other diagnoses: 37	13 min	CT or MRI scan	Trained staff	Detection of stroke/TIA					• This device was not evaluated for its ability to distinguish ICH from other diagnoses.
								65%	80%	78.2	NR	NR	

Abbreviations: AUC, area under curve; CI, confidence interval; CT, computed tomography; EEG, electroencephalography; HS, haemorrhagic stroke; ICH, intracerebral haemorrhage; IS, ischaemic stroke; MCA, middle cerebral artery; MRI, magnetic resonance imaging; NPV, negative predictive value; NR, not reported; PCA, posterior cerebral artery; PPV, positive predictive value; SEN, sensitivity; SM, stroke mimic; SPE, specificity; TCCS, transcranial colour-coded duplex sonography; TIA, transient ischaemic attack; UK, United Kingdom; USA, United States of America; VIPS, volumetric impedance phase shift spectroscopy.

For peer review only

For peer review only - http://bmjopen.bmj.com/site/about/guidelines.xhtml

Prehospital identification of intracerebral haemorrhage: a scoping review of early clinical features and portable devices

Journal:	*BMJ Open*
Manuscript ID	bmjopen-2023-079316.R2
Article Type:	Original research
Date Submitted by the Author:	26-Mar-2024

Complete List of Authors:	Almubayyidh, Mohammed; The University of Manchester, Division of Cardiovascular Sciences; King Saud University, Prince Sultan Bin Abdulaziz College for Emergency Medical Services, Department of Aviation and Marine<br>Alghamdi, Ibrahim; The University of Manchester, Division of Cardiovascular Sciences; King Khalid University, Department of Emergency Medical Services<br>Parry-Jones, Adrian; The University of Manchester, Division of Cardiovascular Sciences; Northern Care Alliance NHS Foundation Trust, Manchester Centre for Clinical Neurosciences<br>Jenkins, David; The University of Manchester, Division of Informatics, Imaging and Data Science

**Primary Subject Heading**:	Emergency medicine
Secondary Subject Heading:	Neurology
Keywords:	Stroke < NEUROLOGY, STROKE MEDICINE, ACCIDENT & EMERGENCY MEDICINE

SCHOLARONE™
Manuscripts

I, the Submitting Author has the right to grant and does grant on behalf of all authors of the Work (as defined in the below author licence), an exclusive licence and/or a non-exclusive licence for contributions from authors who are: i) UK Crown employees; ii) where BMJ has agreed a CC-BY licence shall apply, and/or iii) in accordance with the terms applicable for US Federal Government officers or employees acting as part of their official duties; on a worldwide, perpetual, irrevocable, royalty-free basis to BMJ Publishing Group Ltd ("BMJ") its licensees and where the relevant Journal is co-owned by BMJ to the co-owners of the Journal, to publish the Work in this journal and any other BMJ products and to exploit all rights, as set out in our licence.

The Submitting Author accepts and understands that any supply made under these terms is made by BMJ to the Submitting Author unless you are acting as an employee on behalf of your employer or a postgraduate student of an affiliated institution which is paying any applicable article publishing charge ("APC") for Open Access articles. Where the Submitting Author wishes to make the Work available on an Open Access basis (and intends to pay the relevant APC), the terms of reuse of such Open Access shall be governed by a Creative Commons licence – details of these licences and which Creative Commons licence will apply to this Work are set out in our licence referred to above.

Other than as permitted in any relevant BMJ Author's Self Archiving Policies, I confirm this Work has not been accepted for publication elsewhere, is not being considered for publication elsewhere and does not duplicate material already published. I confirm all authors consent to publication of this Work and authorise the granting of this licence.

For peer review only

Prehospital identification of intracerebral haemorrhage: a scoping review of early clinical features and portable devices

Mohammed Almubayyidh[1,2*], Ibrahim Alghamdi[1,3], Adrian Parry-Jones[1,4], David A Jenkins[5]

[1]Division of Cardiovascular Sciences, The University of Manchester, Manchester, UK

[2]Department of Aviation and Marine, Prince Sultan Bin Abdulaziz College for Emergency Medical Services, King Saud University, Riyadh, Saudi Arabia

[3]Department of Emergency Medical Services, College of Applied Medical Sciences, Khamis Mushait Campus, King Khalid University, Abha, Saudi Arabia

[4]Manchester Centre for Clinical Neurosciences, Northern Care Alliance NHS Foundation Trust, Salford, UK

[5]Division of Informatics, Imaging and Data Science, The University of Manchester, Manchester, UK

*Corresponding author:

Mohammed Almubayyidh, mohammed.almubayyidh@postgrad.manchester.ac.uk, Division of Cardiovascular Sciences, The University of Manchester, Manchester, UK

Abstract word count: 276

Text word count: 5224

For peer review only - http://bmjopen.bmj.com/site/about/guidelines.xhtml

ABSTRACT

Introduction

Prehospital identification of intracerebral haemorrhage (ICH) in suspected stroke cases may enable the initiation of appropriate treatments and facilitate better-informed transport decisions. This scoping review aims to examine the literature to identify early clinical features and portable devices for the detection of ICH in the prehospital setting.

Methods

Three databases were searched via Ovid (MEDLINE, EMBASE, and CENTRAL) from inception to August 2022 using pre-specified search strategies. One reviewer screened all titles, abstracts, and full-text articles for eligibility, while a second reviewer independently screened 20% of the literature during each screening stage. Data extracted were tabulated to summarise the key findings.

Results

A total of 6,803 articles were screened for eligibility, of which 22 studies were included for analysis. Among them, 15 studies reported on early clinical features, while 7 considered portable devices. Associations between age, sex, and comorbidities with the presence of ICH varied across studies. However, most studies reported that patients with ICH exhibited more severe neurological deficits (n=6) and higher blood pressure levels (n=11) at onset compared to other stroke and non-stroke diagnoses. Four technologies were identified for ICH detection: microwave imaging technology, volumetric impedance phase shift spectroscopy, transcranial ultrasound, and electroencephalography. Microwave and ultrasound imaging techniques showed promise in distinguishing ICH from other diagnoses.

Conclusion

This scoping review has identified potential clinical features for the identification of ICH in suspected stroke patients. However, the considerable heterogeneity among the

For peer review only - http://bmjopen.bmj.com/site/about/guidelines.xhtml

included studies precludes meta-analysis of available data. Moreover, we have explored portable devices to enhance ICH identification. While these devices have shown promise in detecting ICH, further technological development is required to distinguish between stroke subtypes (ICH vs ischaemic stroke) and non-stroke diagnoses.

Keywords: Prehospital; Stroke; Intracerebral haemorrhage; Clinical features; Technology; Differential diagnosis.

STRENGTHS AND LIMITATIONS OF THIS STUDY

- To the best of our knowledge, this is the first review focused on identifying spontaneous intracerebral haemorrhage (ICH) in the prehospital setting.
- No time of publication restrictions were applied to comprehensively map the available evidence.
- The review is limited to English language publications only.
- Consistent with the scoping review methodology, a formal risk of bias assessment was not conducted.

For peer review only

For peer review only - http://bmjopen.bmj.com/site/about/guidelines.xhtml

INTRODUCTION

Prehospital recognition of intracerebral haemorrhage (ICH) among suspected stroke cases is not part of standard care, but the ability to reliably do so may pave the way for much quicker delivery of time-critical treatments and triage to a hospital that can provide appropriate interventions (e.g., hyperacute stroke care, neurosurgery) [1,2]. The importance of the rapid identification of ICH patients has been highlighted by studies showing that early haematoma expansion (HE) frequently occurs within 2–3 hours of onset [2-4]. Thus, lowering blood pressure (BP) as well as administering anticoagulant reversal/haemostatic agents may limit HE, thereby increasing the likelihood of favourable outcomes [2,5]. These treatment strategies are currently under investigation in prehospital settings [6-8]. Facilitating the diagnosis of ICH in ambulances through simple and cost-effective methods could support the broader implementation of such therapeutic trials in the future.

While the initial diagnosis of stroke in the prehospital setting is based on stroke recognition tools (e.g., FAST—Face Arm Speech Test, CPSS—Cincinnati Prehospital Stroke Scale), these tools have been validated to identify all strokes rather than ICH specifically [9]. Recognising the importance of timely and accurate identification of ICH, the American Heart Association/American Stroke Association's recent guidelines for the management of patients with ICH have highlighted the need to improve the prehospital recognition of ICH [9]. The recent RACECAT trial further emphasises the significance of prehospital decisions in stroke management, which found that bypassing the closest stroke centre for direct transport to a thrombectomy-capable centre was associated with worse outcomes for patients with ICH [10]. This approach could be more beneficial for patients with large vessel occlusion (LVO) [11], underscoring the necessity for the prehospital differentiation of ICH, especially from LVO [12].

Several clinical features were found to be predictive of ICH in patients with stroke who experience persistent symptoms for more than 24 hours [13]. These features were used to develop clinical prediction rules to distinguish ICH from ischaemic stroke (IS) [13,14]. Nevertheless, the diagnostic accuracy of these rules is low, and they may not

For peer review only - http://bmjopen.bmj.com/site/about/guidelines.xhtml

be applicable to patients presenting within a few hours of the onset of symptoms or during the prehospital phase [15,16]. Such tools for the identification of ICH may have the potential to improve patient outcomes. However, it is unclear which clinical features could be used to identify ICH in prehospital settings. Consequently, brain imaging (computed tomography [CT] and/or magnetic resonance imaging [MRI]) is the only accepted method for diagnosing ICH and other subtypes of stroke. This has led to the development of new portable imaging devices for the detection of patients with stroke in prehospital settings [17,18].

The aim of this study is to perform a scoping review to summarise the early distinguishing clinical features of ICH and evaluate novel portable devices that can help prehospital personnel differentiate ICH from other causes of suspected stroke.

METHODS

The scoping review followed the guidelines of the Joanna Briggs Institute (JBI) [19,20]. It was reported according to the Preferred Reporting Items for Systematic Reviews and Meta-Analyses Extension for Scoping Reviews (PRISMA-ScR) checklist (Appendix 1) [21]. We adopted a scoping review approach because of a broad set of inclusion criteria. A detailed study protocol was published previously [22].

Eligibility criteria

The eligibility criteria for this review were classified using the 'Population, Concept, Context' framework suggested by the JBI (Table 1) [19]. The framework is applied as follows:

- Population: Adult patients (aged ≥16 years) with suspected stroke confirmed by either CT or MRI scans. Studies were excluded if they enrolled children (aged <16 years) or combined the clinical features of spontaneous ICH patients with those of other conditions (e.g., subarachnoid haemorrhage [SAH]) in the same group without reporting them separately.
- Concept: First, to distinguish spontaneous ICH from other stroke and non-stroke diagnoses by investigating the early clinical features observed during the prehospital phase or within the initial 24 hours after the onset of

For peer review only - http://bmjopen.bmj.com/site/about/guidelines.xhtml

symptoms upon arrival at the hospital. Second, to explore the use of portable devices in the detection and classification of ICH. Studies were excluded if they reported clinical features of ICH after 24 hours of symptoms onset, or if they investigated clinical features associated with ICH without comparing them against other suspected stroke cases. Further studies excluded from the review included those that did not provide information on the diagnostic accuracy of the tested technology, examined advances in conventional imaging techniques (CT and MRI), or exclusively tested the technology on phantoms or animal models.

- Context: Prehospital and in-hospital settings with no restrictions on the country of study, ethnicity, gender, or socioeconomic status. Studies conducted in primary care settings were excluded.

Sources of information and search strategy

We included data from peer-reviewed primary research studies published in English. Conference abstracts, commentaries, surveys, case reports, preprints, animal studies, and non-English-language papers were excluded. The literature search of MEDLINE (via Ovid), EMBASE (via Ovid), and CENTRAL (via Ovid) databases was conducted in August 2022, using our pre-specified search strategies (Appendix 2) [22]. To ensure that all relevant studies were included, there was no restriction regarding the date of publication. In addition, we searched the reference lists of retrieved papers to identify additional relevant studies for this review.

Study selection

To eliminate duplicates and facilitate screening and collaboration, the identified studies were imported into EndNote and Rayyan Qatar Computing Research Institute (QCRI) software [23]. Two independent reviewers (MA and IA) selected eligible studies in two stages; initially, titles and abstracts were screened, and then full texts were assessed. MA screened all titles, abstracts, and full-text articles against the eligibility criteria. IA screened a random sample of 20% of the studies at both the title and abstract and full-text stages. Any disagreements in any phase of the selection process

For peer review only - http://bmjopen.bmj.com/site/about/guidelines.xhtml

were resolved by discussion between reviewers to reach consensus or by a third reviewer (APJ or DAJ).

Data extraction

Two data extraction forms (Supplementary Tables 1 and 2) were created using Microsoft Excel, considering the review objectives, the content of the included studies, and insights from previous reviews [15,24]. These tables were designed to present the data from the different studies in a standard format, allowing comparisons to be made between studies and their findings. The data were extracted by the first reviewer (MA), and the second reviewer (IA) independently checked 20% of the extracted data to ensure accuracy.

Data synthesis and quality assessment of studies

Data were summarised as median, mean, and range when appropriate. Otherwise, a narrative approach was used to synthesise the findings of the included studies. In addition, since the PRISMA-ScR checklist does not require an assessment of bias [21], a formal risk of bias assessment was not conducted.

Patient and public involvement

No patients or public were involved in this study.

RESULTS

The search strategies yielded a total of 10,490 articles; after removing duplicates, 6,803 articles remained (Figure 1). Following the initial screening of titles and abstracts, 182 articles remained as potentially relevant and were retrieved for full-text review. After obtaining and reviewing the full texts, 162 studies were excluded with reasons, as illustrated in a PRISMA flowchart in Figure 1. The manual search of the reference lists of included studies revealed two additional studies, resulting in a total of 22 included studies.

For peer review only

For peer review only - http://bmjopen.bmj.com/site/about/guidelines.xhtml

Description of included studies

The retrieved studies were divided into two groups based on the aims of the scoping review: studies that reported the early clinical features of ICH patients (n=15) [25-39] and those that used portable devices to detect ICH (n=7) [40-46]. These groups were divided further according to the data extracted from them.

In the first group (Supplementary Table 1), studies compared the clinical features among various populations, including stroke subtypes (n=12) and non-stroke diagnoses (n=3). Different statistical analyses were used in these studies to determine clinical features, such as univariable (n=3) and multivariable (n=12) regression analyses. Regardless of the statistical techniques used, the reported clinical features of interest were arranged during the analysis process into three groups (Table 2): patients' general information and medical history, early signs and symptoms, and vital signs. Studies that employed portable devices (Supplementary Table 2) to detect or differentiate ICH and other diagnoses were divided into four sub-groups based on the underpinning technology: microwave imaging technology (n=1), volumetric impedance phase shift spectroscopy (VIPS) (n=1), transcranial ultrasound (n=3), and electroencephalography (EEG) (n=2). Additionally, most studies were conducted in Europe (n=13), followed by North America (n=5) and Asia (n=4).

Prediction of ICH in suspected stroke patients using early clinical features

Patients' general information and medical history

Several studies found that patients with ICH were younger (Supplementary Table 1) [25-27,33,36-38], and more likely to be male [29,35,36,38] than those with other stroke and non-stroke diagnoses. However, this association was not uniformly reported, as other studies found that neither age [28,29,31,35,39], nor sex [25-28,31,33,34,39], were predictors of the final diagnosis of ICH. Only one study identified an association between older age and increasing probability of posterior fossa ICH [34].

Several conditions or factors were identified as being more strongly associated with the presence of ICH (vs other diagnoses), including a history of hypertension (HTN)

For peer review only - http://bmjopen.bmj.com/site/about/guidelines.xhtml

[30,34,38], prior ICH [26,31,36], haemodialysis [26], alcohol consumption [36] and the use of warfarin [34]. Conversely, patients with ICH were less likely to have a history of atrial fibrillation (AF) [26,27,29,34-36,38], coronary artery disease (CAD) [25,26,36,38,39], dyslipidaemia [26,34,36,37], diabetes mellitus (DM) [25,27], peripheral arteriopathy (PAD) [36], prior transient ischaemic attack (TIA) [33] or IS [28]. However, other studies found no or weak associations between ICH and HTN [26,27,29,33,35-37], prior TIA/IS [26,36], history of ICH [28], AF [25,33,37,39], CAD [33-35], dyslipidaemia [25,35,38,39], DM [26,31,33-39], PAD [35], alcohol consumption [35,38], or warfarin use [28]. In two studies [25,39], a history of HTN was found to be more common among IS patients than ICH.

Early signs and symptoms of ICH

Several symptoms were reported at the onset of ICH. Among them, headache [26,33,34] and vomiting [26,27,34] were more frequently recorded; however, three studies [25,28,30] did not find these symptoms to be specific to ICH. Additionally, five studies reported that impaired level of consciousness (LOC) was more prevalent among patients with ICH [25,26,29,32,33]. Meanwhile, two studies [28,30] found decreased LOC was not only associated with ICH, but also with LVO and SAH.

Seizures [26] and neurological deficits [26,29], including sensory or motor deficits [26,29], difficulty in following commands [26,29] and impaired vision [29] were also common presentations. Yet six studies [25,28-30,33,34] did not find the presence of seizures [25,29,34], or neurological deficits [25,28,30,33] to be specific discriminators of ICH. In terms of neurological deficits, one study found that compared to IS patients, ICH patients less frequently woke up with symptoms and they experienced greater leg weakness [33].

Eight studies [26,29,34-39] used the National Institutes of Health Stroke Scale (NIHSS) to assess stroke severity either in the prehospital setting [29] or at hospital admission [26,34-39]. Among these studies, six reported higher NIHSS scores at presentation in patients with ICH compared to those with IS, with median scores ranging from 11 to 17 for ICH patients and 6 to 13 for IS patients [26,29,35-38]. However, two studies found no discernible difference in NIHSS scores between ICH and IS patients [34,39].

For peer review only - http://bmjopen.bmj.com/site/about/guidelines.xhtml

Vital signs and their predictive capabilities of ICH

Most of the studies investigated vital signs, including systolic and diastolic BP (SBP and DBP, respectively) [25-29,31-35,37,38], mean arterial pressure (MAP) [29,31], heart rate (HR) or rhythm [25-28,33], peripheral oxygen saturation (SPO_2) [33], and blood glucose level [34,35,38].

In four studies that investigated the prehospital BP [27-29,33], the BP values were dichotomised as follows: SBP, >200 mm Hg [33], ≥180 mm Hg [27,29], ≥165 mm Hg [28]; DBP, ≥110 mm Hg [29], ≥100 mm Hg [27], ≥95 mm Hg [28], >90 mm Hg [33]; MAP, ≥130 mm Hg [29]. These studies found that a higher proportion of ICH patients had elevated BP at presentation than those with other stroke subtypes and non-stroke conditions.

Six studies reported that mean or median SBP [26,31,34,35,37,38], DBP [26,31,34,35,38], and MAP [31] values of ICH patients were higher at the time of onset than in patients with other diagnoses (SBP ranged from 160–204 mm Hg, DBP ranged from 84–102 mm Hg and the MAP was 122.0 ± 20.2 mm Hg). Only one study [25] found no differences in the SBP and DBP values of ICH and IS patients in the prehospital setting. However, another study found that elevated SBP (≥200 mm Hg) in the prehospital setting among patients with stroke with impaired LOC was strongly associated with an increased risk of ICH [32].

With the exception of five studies, no differences were detected in vital signs such as HR [27,33] or rhythm [25,33], SPO_2 [33] and blood glucose [35] between ICH and IS patients. Three of the five studies reported that arrhythmia was less likely to be associated with ICH [26-28], and two observed higher median blood glucose levels at the time of ICH onset (7.1–7.4 mmol/L) compared to IS (6.7–6.8 mmol/L) [34,38].

Portable devices to detect and differentiate ICH

Microwave imaging technology

Two pilot studies were conducted to assess the diagnostic accuracy of microwave-based prototype devices in distinguishing ICH from IS, as well as ICH from healthy individuals [40]. In the first study, 20 patients with stroke were examined within 7–

For peer review only - http://bmjopen.bmj.com/site/about/guidelines.xhtml

132 hours of onset, using the first prototype device by engineering and neurophysiology staff. When aimed at identifying all patients with ICH, the haemorrhagic detector correctly differentiated 7 out of 11 IS patients from the ICH group, with an area under curve (AUC) of 0.88.

The second pilot study involved 25 patients with stroke within 4–27 hours of onset and 65 healthy individuals, with nursing staff testing the second custom-built helmet. When aimed at identifying all patients with ICH, the haemorrhagic detector accurately distinguished 14 out of 15 IS patients from the ICH group, achieving an AUC of 0.85 for distinguishing ICH from IS and 0.87 for distinguishing ICH patients from healthy individuals.

Volumetric impedance phase shift spectroscopy (VIPS)

The effectiveness of the VIPS visor device, developed by Cerebrotech, was investigated in a study aiming to assess its capability to measure the bioimpedance of each brain hemisphere for determining stroke severity [41]. The study enrolled 248 participants, including 41 ‘code stroke’ patients, 79 healthy controls, and 128 patients with various neurological conditions at a comprehensive stroke centre. Trained personnel conducted three scans using the visor device, with a total scan duration of approximately 30 seconds.

The study aimed to evaluate the device's performance in distinguishing severe strokes from minor strokes and severe strokes from all other subjects. Severe strokes were defined as those requiring triage to a comprehensive stroke centre, including conditions such as LVO, NIHSS score of ≥6, ICH of ≥60 ml, and large territorial strokes.

The results of the study demonstrated that patients with severe strokes exhibited the highest mean bioimpedance asymmetry. The VIPS visor device achieved a sensitivity of 93% (95% confidence interval [CI]: 83–98%), specificity of 92% (95% CI: 75–99%), and an AUC of 0.93 (95% CI: 0.85–0.97) when differentiating severe strokes from minor strokes. Similarly, in distinguishing severe strokes from all tested subjects, the device showed a sensitivity of 93% (95% CI: 83–98%), a specificity of 87% (95% CI: 81–92%), and an AUC of 0.93 (95% CI: 0.89–0.96). The ability of VIPS to differentiate ICH from other conditions was not reported in this study.

For peer review only - http://bmjopen.bmj.com/site/about/guidelines.xhtml

Transcranial ultrasound

Three studies utilised transcranial ultrasound imaging to diagnose ICH and distinguish it from other suspected stroke causes [42-44]. In one clinical study [42], transcranial colour-coded duplex sonography (TCCS) scans were conducted on 84 patients using a Sonos 1000 Hewlett-Packard machine. ICH diagnosis was based on sharply demarcated hyperechogenic areas in the brain tissue. Among the patients, 52 had IS or TIA, 15 had ICH, and 17 were excluded due to inadequate temporal bone windows. TCCS identified ICH in 14 patients with focal hyperechogenicity, with only one small haemorrhage missed. Sensitivity and specificity were reported as 88% and 96%, respectively.

In another study [43], the diagnostic accuracy of TCCS was evaluated using Siemens Sonoline Ellegra or Acuson 128 XP/4 on 151 patients with acute hemiparesis. Among them, 60 had ICH, 67 had IS, and 24 had inconclusive CT scans. Ultrasound examination of 18 patients revealed insufficient acoustic bone windows. For the remaining 133 patients, TCCS detected 50 cases of ICH with a sensitivity of 94% and specificity of 95%.

A third study examined 107 suspected stroke patients using SonoSite M-Turbo, Philips Sparq, or Philips CX50 for TCCS [44]. Among the patients, 63 had acute IS, 18 had ICH, 13 had TIA, and 13 had stroke-mimicking conditions. Sonographic examination revealed an insufficient temporal bone window in 18 cases. Ultrasound imaging accurately identified ICH in 56% of patients, but failed in 33% of cases. The study presented a triage model that combined transcranial ultrasound and clinical assessment, resulting in a 10% improvement in ICH detection compared to clinical assessment alone. While the specificity of both techniques was reported as 99%, the sensitivity of the combined model was 63%, whereas clinical assessment alone yielded a sensitivity of 6%.

Electroencephalography (EEG)

Two studies examined the diagnostic capability of portable EEG devices for detecting strokes in emergency departments (ED) [45,46]. In one study [45], BrainScope's handheld device was used to identify strokes in 183 patients presenting with stroke-like symptoms. Among them, 31 patients were diagnosed with IS, 17 with

For peer review only - http://bmjopen.bmj.com/site/about/guidelines.xhtml

haemorrhagic stroke, and 135 with stroke-mimicking conditions. The device recorded EEG data for 10 minutes while the patients kept their eyes closed, and the analysis was conducted using the Structural Brain Injury Index (SBII) algorithm. The SBII showed a sensitivity of 94.1% for detecting haemorrhagic strokes, with a specificity of 50.4% for stroke mimics serving as controls.

Another study assessed the Quick-20 EEG system for diagnosing patients with acute stroke [46]. Out of the 100 patients involved, 63 were diagnosed with acute stroke/TIA, among whom 7 patients had ICH. EEG signals alone achieved a sensitivity of 65% and specificity of 80%, with an AUC of 78.2 for detecting acute stroke/TIA. Combining EEG with clinical data using deep learning improved diagnostic performance, yielding a sensitivity of 79%, a specificity of 80%, and an AUC of 87.8.

DISCUSSION

Overall, we identified fifteen studies that reported early clinical features of ICH among suspected stroke patients and seven studies that tested portable technologies to detect ICH and other conditions.

Early clinical features of ICH in suspected stroke patients

Diagnosing ICH based solely on clinical features can be difficult, as many features of ICH overlap with other types of strokes [47]. This scoping review has identified potential clinical features that can help distinguish ICH patients from those with other stroke types or non-stroke diagnoses. The combination of these features might be useful in improving the early prediction of ICH. However, it is important to note that the included studies investigated and reported different clinical features for ICH patients across various stroke subtypes, and some studies also included TIA and stroke-mimicking conditions in their analyses. In addition, some studies only conducted univariable analysis, and caution is advised when interpreting our results as there could be bias in the studies, such as confounding. Heterogeneity in the definition and categorisation of variables was also observed. Owing to these methodological limitations, we recognise that conducting a meta-analysis of individual

For peer review only - http://bmjopen.bmj.com/site/about/guidelines.xhtml

For peer review only

features would be highly challenging and unlikely to yield meaningful insights relevant to the aims of this review.

Given the considerable heterogeneity among the included studies, identifying consistent predictors for the diagnosis of ICH was challenging. In terms of patients' general information and medical history, neither age nor sex consistently predicted the final diagnosis of ICH across the studies. However, certain factors were reported to be more strongly associated with the presence of ICH, including a history of HTN, prior ICH, haemodialysis, alcohol consumption, and the use of warfarin. In contrast, patients with ICH were less frequently found to have a history of AF, CAD, dyslipidaemia, DM, PAD, prior TIA, or IS. If available in the prehospital setting, electrocardiogram monitoring can be used to assess the presence of AF, which may help differentiate ICH from IS [26,29,48,49]. Nevertheless, the associations between these factors and ICH varied across studies. These results contradict a previous meta-analysis [15] that found a greater likelihood of ICH in male patients aged 60 years or less, while a history of TIA, PAD, and AF increased the probability of IS being diagnosed.

The initial signs and symptoms of ICH, such as headache, vomiting, and decreased LOC, also demonstrated variability across the included studies. While these manifestations were frequently observed, some studies did not find them to be exclusively indicative of ICH. This discrepancy could potentially be attributed to the inclusion of patients with SAH in certain studies [28,30]. SAH patients typically present with a sudden onset of headache, vomiting, decreased LOC, and neck stiffness [50,51]. Consequently, relying solely on these clinical features for the prediction of ICH may lack specificity when compared to SAH patients.

The majority of studies in this review consistently reported that patients with ICH experienced more severe neurological deficits at onset compared to other subtypes of stroke, TIA, and stroke-mimicking conditions. Furthermore, ICH patients were less commonly observed to have neurological deficits upon awakening when compared to patients with IS. These findings are in line with other studies that have reported a higher frequency of hemiplegia/hemiparesis and sensory deficits in patients with

For peer review only - http://bmjopen.bmj.com/site/about/guidelines.xhtml

cerebral haemorrhage [48,50], while stroke symptoms upon awakening were more frequently observed in patients with IS [50,52].

To assess and quantify neurological deficits, more than half of the studies utilised the NIHSS, which consistently showed higher scores in ICH patients. In fact, a recent study investigating the relationship between early NIHSS scores and ICH volume found that for every 10 ml increase in ICH volume, the NIHSS score increased by 4.5 points [53]. However, despite this, the NIHSS is not routinely used by prehospital personnel for stroke assessment due to its complexity and time-consuming nature [54]. In an effort to address these limitations, a recent study demonstrated that the NIHSS can be an accurate and time-efficient tool for paramedics in the field [55], suggesting the potential of utilising the NIHSS items to construct a prehospital prediction model for ICH [29].

Several prediction models have been proposed to differentiate ICH from other suspected stroke patients in the prehospital setting [25,27-30]. These tools can assist prehospital personnel in making accurate and timely decisions for patients with stroke. However, their predictive abilities have not been thoroughly studied, compared, and evaluated in an independent study. Therefore, further research is necessary to assess their performance, reliability, and potential limitations or biases that may impact their effectiveness in real-world settings. The addition of the clinical features found in this review could further improve the performance of these tools.

Another important finding of this study is that patients with ICH had the highest BP parameters among all suspected stroke patients. Notably, there is a strong correlation between elevated SBP of ≥160 mm Hg and the initial haematoma volume in ICH patients [56]. A post-hoc analysis of the FAST-MAG trial revealed a distinct association between SBP and ultra-early neurological deterioration (defined as a deterioration of 2 or more points on the Glasgow Coma Scale within 2 hours of stroke onset) among ICH patients [57]. According to the study, neurological deterioration in patients with ICH was associated with higher SBP upon arrival at the ED. Additionally, imaging performed upon arrival showed that ICH patients with neurological deterioration had much larger haematoma volumes, suggesting that early HE may contribute to the deterioration [57]. Previous studies have also found an association between

For peer review only - http://bmjopen.bmj.com/site/about/guidelines.xhtml

prehospital BP values (SBP and DBP) and the occurrence of neurological deterioration in ICH [58,59]. These findings partially align with our results, in which there was a very powerful relationship between increased prehospital SBP and the occurrence of ICH among patients with stroke with impaired LOC [32].

Other vital signs, such as HR and $SPO_{2,}$ were compared in some of the included studies and seemed to be insignificant in predicting the presence or absence of ICH. However, associations appear evident between cardiac arrhythmias in patients with IS or LVO, while higher blood glucose levels were reported in patients with ICH upon hospital admission.

Portable technologies for ICH detection and differentiation

Numerous portable technologies have been designed to identify and distinguish ICH from other stroke subtypes or stroke-mimicking diagnoses. In addition to clinical features, this scoping review has identified portable devices that have the potential to improve the detection of ICH and could be used in various settings, including prehospital care.

In recent years, significant progress has been made in the prehospital diagnosis and treatment of ICH patients with the introduction of mobile stroke units (MSUs) [2,60,61]. However, due to the high costs associated with this technology, it is unlikely to become widely available for stroke care in the near future [62]. Thus, portable devices may serve as cost-effective alternatives to MSUs in the prehospital setting.

Some of the technologies evaluated showed greater accuracy than clinical assessments [41,44,46]. Antipova et al. [44] developed a triage model that combined transcranial sonographic findings with clinical assessment, resulting in significantly improved diagnostic sensitivity for detecting ICH. Hence, the combination of clinical features with the findings of these systems might further enhance the diagnostic accuracy of ICH.

The reviewed studies also highlighted the potential benefits of using these technologies for rapid diagnosis in the prehospital setting. For instance, in the VITAL study [41], the VIPS technology demonstrated an average detection time of 30

For peer review only - http://bmjopen.bmj.com/site/about/guidelines.xhtml

seconds for severe strokes. Other technologies evaluated had median application times ranging from 10 to 20 minutes (Supplementary Table 2), depending on the specific technology employed. Shortening the time-to-treatment duration is crucial in stroke management to improve patient outcomes [63]. Therefore, it is important to ensure that the use of diagnostic technologies does not cause delays in transporting patients to definitive care.

A number of limitations were identified for each technology. Most notably, the discriminatory ability to differentiate ICH from other diseases was not evaluated with VIPS and portable EEGs [41,45,46]. Conversely, microwave and ultrasound imaging techniques yielded good results in differentiating between stroke subtypes [40,42-44]. However, the diagnostic accuracy of microwave technology to discriminate ICH from IS could be decreased with the development of cerebral oedema after IS [40]. On the other hand, transcranial ultrasound cannot produce useful images unless adequate temporal bone windows are available [42-44]. Furthermore, with this technology, pathological conditions such as haemorrhagic transformation of infarcts and gliomas could be misdiagnosed as ICH [42,43].

While several diagnostic modalities discussed in this review show promise for prehospital stroke care, their diagnostic accuracy requires further improvement and validation using a large, diverse cohort of suspected stroke patients. Such validation is crucial to establish their clinical usefulness in the prehospital setting, where both sensitivity and specificity are important in determining appropriate management. Given the significant differences in treatment strategies between ICH and IS, it is imperative that the detection technologies can effectively differentiate between the two with high sensitivity and specificity. Reliable differentiation can enable the prompt initiation of appropriate treatment measures for ICH, including BP reduction and anticoagulation reversal, and facilitate triaging these patients to neurosurgical-capable facilities. Moreover, consideration should be given to the level of training required for personnel to competently operate these technologies [24]. For instance, devices such as transcranial ultrasound necessitate highly trained operators, which may limit their feasibility in the prehospital setting.

For peer review only - http://bmjopen.bmj.com/site/about/guidelines.xhtml

Other novel approaches to detect and distinguish ICH

There are other novel technologies that exist for detecting and distinguishing ICH, but these fall beyond the scope of this review, either due to the technologies not being investigated in suspected stroke cases or their lack of portability. One of the most studied among them is near-infrared spectroscopy (NIRS). In a pilot study, the handheld NIRS Infrascanner was evaluated for its ability to detect intracranial haematomas in 35 patients with traumatic brain injuries [64]. When compared to CT scans, which were considered the gold standard, the Infrascanner demonstrated a sensitivity of 88.89% (95% CI: 50.7–99.4%) and specificity of 81.25% (95% CI: 53.7–95.0%) in identifying intraparenchymal haematomas. However, its effectiveness in accurately distinguishing between stroke subtypes, particularly in identifying deep ICH, may be hindered by its limited detection range of approximately 3 cm beneath the skull [64,65].

Besides brain diagnostic devices, the use of blood biomarkers is also a promising approach for the early detection and differentiation of stroke subtypes [37,66]. However, it remains unclear whether they can be used as a point-of-care diagnostic method in the prehospital setting [67]. This is currently being investigated in a prehospital trial (Biomarkers for Initiating Onsite and Faster Ambulance Stroke Therapies, Bio-FAST, ClinicalTrials.gov Identifier: NCT04612218) with point-of-care test devices.

Limitations of the review

Our scoping review has some limitations that should be considered. Only one reviewer screened and analysed all of the data. Although a second reviewer independently screened 20% of the articles at the abstract, full-text, and data extraction stages, there is a risk of review errors and bias. Additionally, the search was limited to English publications, which could have resulted in the exclusion of important studies published in other languages. Furthermore, some of the included studies examined the clinical features of ICH up to 24 hours of symptoms onset, which requires cautious interpretation of the findings regarding their external validity for prehospital evaluations. Lastly, owing to the nature of scoping reviews, the methodological quality

For peer review only - http://bmjopen.bmj.com/site/about/guidelines.xhtml

of the studies included was not assessed [20]. This means that poor-quality studies were given equal weight as high-quality studies, which should be taken into account when interpreting the review findings.

CONCLUSION AND FUTURE WORK

Our study identifies potential early clinical features of ICH that distinguish it from suspected stroke patients. However, the substantial heterogeneity observed in populations, clinical features, and outcome variables among the included studies precludes the combination of these features in a meta-analysis. The variability in findings across studies highlights the need for further research and standardised tools to improve early prediction and differentiation of ICH. Future studies should focus on developing and validating predictive models or tools that incorporate easily obtainable clinical features for prehospital personnel. Additionally, it is important to consider an unselected cohort of suspected stroke patients in future studies to ensure that the findings are more widely applicable and representative of real-world practice. Furthermore, large independent cohorts should be used to evaluate the performance, reliability, and potential limitations of these predictive tools.

In addition, this study presents portable technologies for the detection of ICH and other stroke subtypes. Whilst some of these technologies have shown promising results, the majority are in the early stages of development and require further improvement and validation in large cohorts of suspected stroke cases. Furthermore, the selected studies in this review reported different study designs, comparison groups, and diagnostic aims, making it challenging to draw definitive conclusions about the superiority or preference for one technology over another. To better understand the potential benefits and feasibility of using portable devices for prehospital stroke care, future research should not only focus on reporting the diagnostic performance of the devices but also consider the practical aspects of their use in prehospital settings. This includes assessing the training requirements for device use, the time needed to acquire and interpret results, and the ability of the technology to differentiate between stroke subtypes (ICH vs IS) and non-stroke diagnoses.

For peer review only - http://bmjopen.bmj.com/site/about/guidelines.xhtml

ACKNOWLEDGEMENTS

The authors wish to express their sincere gratitude to the peer reviewers of this study for their valuable input and suggestions.

FUNDING STATEMENT

This study is part of MA's PhD research, which is funded by King Saud University, Riyadh, Saudi Arabia (Grant no: NA), through the Saudi Arabian Cultural Bureau in the United Kingdom (Grant no: NA).

For peer review only

COMPETING INTERESTS

None declared.

AUTHOR CONTRIBUTIONS

All authors included in this paper fulfil the criteria of authorship. MA is the primary author of this work under the supervision of APJ and DAJ. DAJ conceptualised the idea for the review. MA and IA screened the studies and performed data extraction. MA conducted data analyses and wrote the manuscript with support from APJ and DAJ. The manuscript was critically reviewed and approved by all authors for final submission.

ETHICS APPROVAL

This is a scoping review; therefore, ethical approval is not required.

DATA AVAILABILITY STATEMENT

All data relevant to this study are included in the article or uploaded as supplementary information.

For peer review only - http://bmjopen.bmj.com/site/about/guidelines.xhtml

Table 1. Inclusion and exclusion criteria.

PCC	Included	Excluded
Population	• Studies of adult patients (≥16 years old) with suspected stroke.	• Studies of children (<16 years old).<br>• Studies combined ICH clinical features with other aetiologies.
Concept	• Studies reported the clinical features of suspected stroke patients, obtained during prehospital care or within 24 hours of symptoms onset upon hospital admission.<br>• Studies evaluated portable devices to detect ICH.	• Studies examined the clinical features of patients after 24 hours of symptoms onset or focused on ICH-related clinical features without comparisons to other suspected stroke cases.<br>• Studies that failed to provide information on the diagnostic performance of the technology being tested, or examined advances in conventional detection methods, or restricted their testing to phantoms or animal models.
Context	• Prehospital and in-hospital studies.	• Primary care studies.

Abbreviations: ICH, intracerebral haemorrhage; PCC, population, concept, context.

For peer review only

Table 2. Summary of reported clinical features for predicting ICH in suspected stroke patients.

Category	Findings	References
Patients' general information and medical history	• Age and sex showed inconsistent predictive value for ICH.<br>• Hypertension, prior ICH, haemodialysis, alcohol consumption, and warfarin use were more frequently associated with ICH.<br>• Atrial fibrillation, coronary artery disease, dyslipidaemia, diabetes mellitus, peripheral artery disease, and previous TIA/IS were less commonly associated with ICH.<br>• Some studies found weak or no associations between the aforementioned factors or conditions and the prediction of ICH.	[25-31,33-39]
Signs and symptoms	• Headaches, vomiting, impaired consciousness, and seizures were reported, but these symptoms were not specific to ICH.<br>• Awakening with stroke symptoms was less likely to be associated with ICH.<br>• Patients with ICH exhibited more neurological deficits as measured by the NIHSS.	[25-30,32-39]
Vital signs	• Elevated blood pressure levels were more consistently observed at the onset of ICH.<br>• Arrhythmias were less frequently seen in patients with ICH.<br>• High blood glucose levels were reported in patients with ICH.	[25-29,31-35,37,38]

Abbreviations: ICH, intracerebral haemorrhage; IS, ischaemic stroke; NIHSS, National Institutes of Health Stroke Scale; TIA, transient ischaemic attack.

For peer review only - http://bmjopen.bmj.com/site/about/guidelines.xhtml

FIGURE CAPTION

Figure 1. Flowchart of study selection process (*search results for clinical features + portable devices, respectively).

For peer review only

For peer review only - http://bmjopen.bmj.com/site/about/guidelines.xhtml

REFERENCES

1. Colton K, Richards CT, Pruitt PB, et al. Early Stroke Recognition and Time-based Emergency Care Performance Metrics for Intracerebral Hemorrhage. J Stroke Cerebrovasc Dis. 2020;29(2):104552.

2. Bowry R, Parker SA, Bratina P, et al. Hemorrhage Enlargement Is More Frequent in the First 2 Hours: A Prehospital Mobile Stroke Unit Study. Stroke. 2022;53(7):2352-60.

3. Brott T, Broderick J, Kothari R, et al. Early hemorrhage growth in patients with intracerebral hemorrhage. Stroke. 1997;28(1):1-5.

4. Broderick JP, Diringer MN, Hill MD, et al. Determinants of intracerebral hemorrhage growth: an exploratory analysis. Stroke. 2007;38(3):1072-5.

5. Rodriguez-Luna D, Rodriguez-Villatoro N, Juega JM, et al. Prehospital Systolic Blood Pressure Is Related to Intracerebral Hemorrhage Volume on Admission. Stroke. 2018;49(1):204-6.

6. Song L, Chen C, Chen X, et al. INTEnsive ambulance-delivered blood pressure Reduction in hyper-ACute stroke Trial (INTERACT4): study protocol for a randomized controlled trial. Trials. 2021;22(1):885.

7. Yassi N, Zhao H, Churilov L, et al. Tranexamic acid for intracerebral haemorrhage within 2 hours of onset: protocol of a phase II randomised placebo-controlled double-blind multicentre trial. Stroke Vasc Neurol. 2022;7(2):158-65.

8. Naidech AM, Grotta J, Elm J, et al. Recombinant factor VIIa for hemorrhagic stroke treatment at earliest possible time (FASTEST): Protocol for a phase III, double-blind, randomized, placebo-controlled trial. Int J Stroke. 2021:806-9.

9. Greenberg SM, Ziai WC, Cordonnier C, et al. 2022 Guideline for the Management of Patients With Spontaneous Intracerebral Hemorrhage: A Guideline From the American Heart Association/American Stroke Association. Stroke. 2022;53(7):e282-e361.

10. Ramos-Pachón A, Rodríguez-Luna D, Martí-Fàbregas J, et al. Effect of Bypassing the Closest Stroke Center in Patients with Intracerebral Hemorrhage: A Secondary Analysis of the RACECAT Randomized Clinical Trial. JAMA Neurol. 2023;80(10):1028-36.

For peer review only

For peer review only - http://bmjopen.bmj.com/site/about/guidelines.xhtml

11. Nguyen TTM, van den Wijngaard IR, Bosch J, et al. Comparison of Prehospital Scales for Predicting Large Anterior Vessel Occlusion in the Ambulance Setting. JAMA Neurol. 2021;78(2):157-64.

12. Richards CT, Oostema JA, Chapman SN, et al. Prehospital Stroke Care Part 2: On-Scene Evaluation and Management by Emergency Medical Services Practitioners. Stroke. 2023;54(5):1416-25.

13. Sandercock PA, Allen CM, Corston RN, et al. Clinical diagnosis of intracranial haemorrhage using Guy's Hospital score. Br Med J (Clin Res Ed). 1985;291(6510):1675-7.

14. Poungvarin N, Viriyavejakul A, Komontri C. Siriraj stroke score and validation study to distinguish supratentorial intracerebral haemorrhage from infarction. BMJ. 1991;302(6792):1565-7.

15. Runchey S, McGee S. Does this patient have a hemorrhagic stroke? Clinical findings distinguishing hemorrhagic stroke from ischemic stroke. JAMA. 2010;303(22):2280-6.

16. Badam P, Solao V, Pai M, et al. Poor accuracy of the Siriraj and Guy's hospital stroke scores in distinguishing haemorrhagic from ischaemic stroke in a rural, tertiary care hospital. Natl Med J India. 2003;16(1):8-12.

17. Walsh KB. Non-invasive sensor technology for prehospital stroke diagnosis: Current status and future directions. Int J Stroke. 2019;14(6):592-602.

18. Lin MP, Sanossian N, Liebeskind DS. Imaging of prehospital stroke therapeutics. Expert Rev Cardiovasc Ther. 2015;13(9):1001-15.

19. Peters M, Godfrey C, Khalil H, et al. 2017 Guidance for the Conduct of JBI Scoping Reviews. 2017.

20. Peters MDJ, Marnie C, Colquhoun H, et al. Scoping reviews: reinforcing and advancing the methodology and application. Syst Rev. 2021;10(1):263.

21. Tricco AC, Lillie E, Zarin W, et al. PRISMA Extension for Scoping Reviews (PRISMA-ScR): Checklist and Explanation. Ann Intern Med. 2018;169(7):467-73.

22. Almubayyidh M, Alghamdi I, Parry-Jones AR, et al. Clinical features and novel technologies for prehospital detection of intracerebral haemorrhage: a scoping review protocol. BMJ Open. 2023;13(5):e070228.

23. Ouzzani M, Hammady H, Fedorowicz Z, et al. Rayyan-a web and mobile app for systematic reviews. Syst Rev. 2016;5(1):210.

For peer review only - http://bmjopen.bmj.com/site/about/guidelines.xhtml

24. Chennareddy S, Kalagara R, Smith C, et al. Portable stroke detection devices: a systematic scoping review of prehospital applications. BMC Emerg Med. 2022;22(1):111.

25. Woisetschläger C, Kittler H, Oschatz E, et al. Out-of-hospital diagnosis of cerebral infarction versus intracranial hemorrhage. Intensive Care Med. 2000;26(10):1561-5.

26. Yamashita S, Kimura K, Iguchi Y, et al. Kurashiki Prehospital Stroke Subtyping Score (KP3S) as a means of distinguishing ischemic from hemorrhagic stroke in emergency medical services. Eur Neurol. 2011;65(4):233-8.

27. Jin HQ, Wang JC, Sun YA, et al. Prehospital Identification of Stroke Subtypes in Chinese Rural Areas. Chin Med J (Engl). 2016;129(9):1041-6.

28. Uchida K, Yoshimura S, Hiyama N, et al. Clinical Prediction Rules to Classify Types of Stroke at Prehospital Stage. Stroke. 2018;49(8):1820-7.

29. Geisler F, Wesirow M, Ebinger M, et al. Probability assessment of intracerebral hemorrhage in prehospital emergency patients. Neurol Res Pract. 2021;3(1):1.

30. Chiquete E, Jiménez-Ruiz A, García-Grimshaw M, et al. Prediction of acute neurovascular syndromes with prehospital clinical features witnessed by bystanders. Neurol Sci. 2021;42(8):3217-24.

31. Gioia LC, Zewude RT, Kate MP, et al. Prehospital systolic blood pressure is higher in acute stroke compared with stroke mimics. Neurology. 2016;86(23):2146-53.

32. Irisawa T, Iwami T, Kitamura T, et al. An association between systolic blood pressure and stroke among patients with impaired consciousness in out-of-hospital emergency settings. BMC Emerg Med. 2013;13:24.

33. Andersson Hagiwara M, Wireklint Sundström B, Brink P, et al. A shorter system delay for haemorrhagic stroke than ischaemic stroke among patients who use emergency medical service. Acta Neurol Scand. 2018;137(5):523-30.

34. Puustjärvi V, Strbian D, Tiainen M, et al. Recognition of posterior circulation stroke. Acta Neurol Scand. 2015;131(6):389-93.

35. Bustamante A, Penalba A, Orset C, et al. Blood Biomarkers to Differentiate Ischemic and Hemorrhagic Strokes. Neurology. 2021;96(15):e1928-e39.

36. Montaner J, Mendioroz M, Delgado P, et al. Differentiating ischemic from hemorrhagic stroke using plasma biomarkers: the S100B/RAGE pathway. J Proteomics. 2012;75(15):4758-65.

For peer review only - http://bmjopen.bmj.com/site/about/guidelines.xhtml

37. Rozanski M, Waldschmidt C, Kunz A, et al. Glial Fibrillary Acidic Protein for Prehospital Diagnosis of Intracerebral Hemorrhage. Cerebrovasc Dis. 2017;43(1-2):76-81.

38. Bustamante A, López-Cancio E, Pich S, et al. Blood Biomarkers for the Early Diagnosis of Stroke: The Stroke-Chip Study. Stroke. 2017;48(9):2419-25.

39. Llombart V, García-Berrocoso T, Bustamante A, et al. Plasmatic retinol-binding protein 4 and glial fibrillary acidic protein as biomarkers to differentiate ischemic stroke and intracerebral hemorrhage. J Neurochem. 2016;136(2):416-24.

40. Persson M, Fhager A, Trefná HD, et al. Microwave-based stroke diagnosis making global prehospital thrombolytic treatment possible. IEEE Trans Biomed Eng. 2014;61(11):2806-17.

41. Kellner CP, Sauvageau E, Snyder KV, et al. The VITAL study and overall pooled analysis with the VIPS non-invasive stroke detection device. J Neurointerv Surg. 2018;10(11):1079-84.

42. Seidel G, Kaps M, Gerriets T. Potential and limitations of transcranial color-coded sonography in stroke patients. Stroke. 1995;26(11):2061-6.

43. Mäurer M, Shambal S, Berg D, et al. Differentiation between intracerebral hemorrhage and ischemic stroke by transcranial color-coded duplex-sonography. Stroke. 1998;29(12):2563-7.

44. Antipova D, Eadie L, Makin S, et al. The use of transcranial ultrasound and clinical assessment to diagnose ischaemic stroke due to large vessel occlusion in remote and rural areas. PLoS One. 2020;15(10):e0239653.

45. Michelson EA, Hanley D, Chabot R, et al. Identification of acute stroke using quantified brain electrical activity. Acad Emerg Med. 2015;22(1):67-72.

46. Erani F, Zolotova N, Vanderschelden B, et al. Electroencephalography Might Improve Diagnosis of Acute Stroke and Large Vessel Occlusion. Stroke. 2020;51(11):3361-5.

47. Broderick J, Connolly S, Feldmann E, et al. Guidelines for the Management of Spontaneous Intracerebral Hemorrhage in Adults. Stroke. 2007;38(6):2001-23.

48. Besson G, Robert C, Hommel M, et al. Is It Clinically Possible to Distinguish Nonhemorrhagic Infarct From Hemorrhagic Stroke? Stroke. 1995;26(7):1205-9.

For peer review only - http://bmjopen.bmj.com/site/about/guidelines.xhtml

49. Efstathiou SP, Tsioulos DI, Zacharos ID, et al. A new classification tool for clinical differentiation between haemorrhagic and ischaemic stroke. J Intern Med. 2002;252(2):121-9.

50. Massaro AR, Sacco RL, Scaff M, et al. Clinical discriminators between acute brain hemorrhage and infarction: a practical score for early patient identification. Arq Neuropsiquiatr. 2002;60(2-a):185-91.

51. Edlow JA, Caplan LR. Avoiding pitfalls in the diagnosis of subarachnoid hemorrhage. N Engl J Med. 2000;342(1):29-36.

52. Lee SE, Choi MH, Kang HJ, et al. Stepwise stroke recognition through clinical information, vital signs, and initial labs (CIVIL): Electronic health record-based observational cohort study. PLoS One. 2020;15(4):e0231113.

53. Farooq S, Shkirkova K, Villablanca P, et al. National Institutes of Health Stroke Scale Correlates Well with Initial Intracerebral Hemorrhage Volume. J Stroke Cerebrovasc Dis. 2022;31(4):106348.

54. Larsen K, Hov MR, Sandset EC. Prehospital stroke scales-the need for a gold standard in the field. Acta Neurol Scand. 2022;145(3):263-4.

55. Larsen K, Jæger HS, Hov MR, et al. Streamlining Acute Stroke Care by Introducing National Institutes of Health Stroke Scale in the Emergency Medical Services: A Prospective Cohort Study. Stroke. 2022;53(6):2050-7.

56. Ohwaki K, Yano E, Nagashima H, et al. Blood pressure management in acute intracerebral hemorrhage: relationship between elevated blood pressure and hematoma enlargement. Stroke. 2004;35(6):1364-7.

57. Shkirkova K, Saver JL, Starkman S, et al. Frequency, Predictors, and Outcomes of Prehospital and Early Postarrival Neurological Deterioration in Acute Stroke: Exploratory Analysis of the FAST-MAG Randomized Clinical Trial. JAMA Neurol. 2018;75(11):1364-74.

58. Tsou YJ, Lan KP, Fan JS. Relationship Between Changes in Prehospital Blood Pressure and Early Neurological Deterioration in Spontaneous Intracerebral Hemorrhage. Adv Emerg Nurs J. 2019;41(2):163-71.

59. Moon JS, Janjua N, Ahmed S, et al. Prehospital neurologic deterioration in patients with intracerebral hemorrhage. Crit Care Med. 2008;36(1):172-5.

For peer review only - http://bmjopen.bmj.com/site/about/guidelines.xhtml

60. Gomes JA, Ahrens CL, Hussain MS, et al. Prehospital reversal of warfarin-related coagulopathy in intracerebral hemorrhage in a mobile stroke treatment unit. Stroke. 2015;46(5):e118-20.
61. Cooley SR, Zhao H, Campbell BCV, et al. Mobile Stroke Units Facilitate Prehospital Management of Intracerebral Hemorrhage. Stroke. 2021;52(10):3163-6.
62. Walter S, Grunwald IQ, Helwig SA, et al. Mobile Stroke Units - Cost-Effective or Just an Expensive Hype? Curr Atheroscler Rep. 2018;20(10):49.
63. Pulvers JN, Watson JDG. If Time Is Brain Where Is the Improvement in Prehospital Time after Stroke? Front Neurol. 2017;8:617.
64. Leon-Carrion J, Dominguez-Roldan JM, Leon-Dominguez U, et al. The Infrascanner, a handheld device for screening in situ for the presence of brain haematomas. Brain Inj. 2010;24(10):1193-201.
65. Kontojannis V, Hostettler I, Brogan RJ, et al. Detection of intracranial hematomas in the emergency department using near infrared spectroscopy. Brain Inj. 2019;33(7):875-83.
66. Mattila OS, Ashton NJ, Blennow K, et al. Ultra-Early Differential Diagnosis of Acute Cerebral Ischemia and Hemorrhagic Stroke by Measuring the Prehospital Release Rate of GFAP. Clin Chem. 2021;67(10):1361-72.
67. Dagonnier M, Donnan GA, Davis SM, et al. Acute Stroke Biomarkers: Are We There Yet? Front Neurol. 2021;12:619721.

For peer review only

For peer review only - http://bmjopen.bmj.com/site/about/guidelines.xhtml

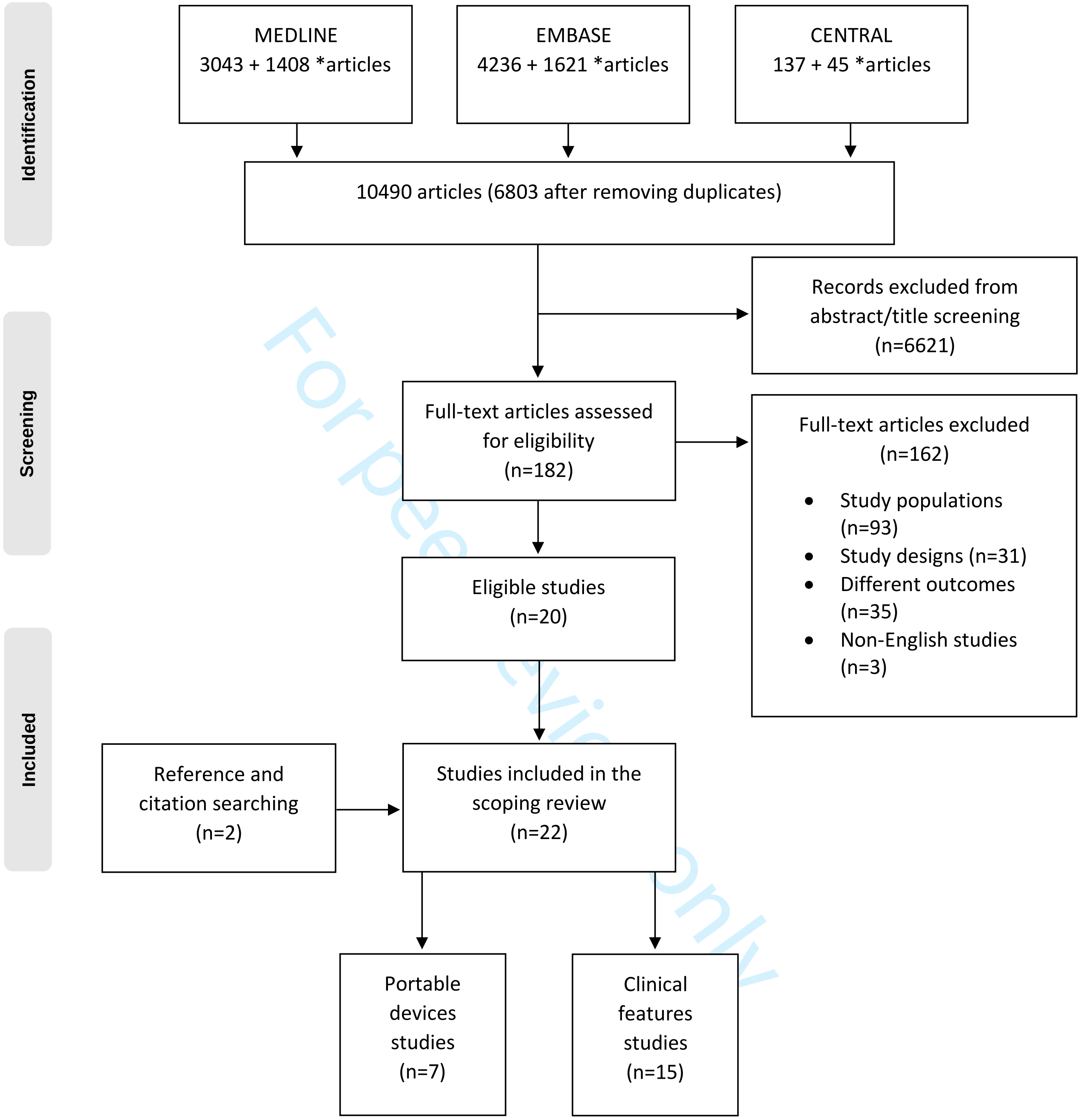
Identification
Screening
Included
MEDLINE
3043 + 1408 *articles
EMBASE
4236 + 1621 *articles
CENTRAL
137 + 45 *articles
10490 articles (6803 after removing duplicates)
Records excluded from abstract/title screening (n=6621)
Full-text articles assessed for eligibility (n=182)
Full-text articles excluded (n=162)
Study populations (n=93)
Study designs (n=31)
Different outcomes (n=35)
Non-English studies (n=3)
Eligible studies (n=20)
Reference and citation searching (n=2)
Studies included in the scoping review (n=22)
Portable devices studies (n=7)
Clinical features studies (n=15)

Supplementary Table 1. Characteristics of the included studies for clinical features.

Included study, year and country	Design	Setting	Study population	Statistical method	Age (Years)	Onset-to-evaluation	Method of confirmation	Clinical features collected			Findings relevant to review
								Past medical history	Signs and symptoms	Vital signs	
Woisetschläger [25], 2000; Austria	Retrospective-prospective study	Prehospital and in-hospital	ICH, IS<br><br>ICH: 118<br>IS: 106	Uni- and multi-variable regression	ICH (mean): 58<br>IS (mean): 65	NR (Evaluated in prehospital)	CT scan	• AF<br>• Aspirin use<br>• Dicoumarin use<br>• DM<br>• HTN<br>• Hyperlipidaemia<br>• Prior MI<br>• Smoking	• Aphasia<br>• Headache<br>• Hemisymptoms<br>• Impaired LOC<br>• Seizures<br>• Syncope<br>• Vomiting	• SBP<br>• DBP<br>• ECG	• ICH patients were younger than IS patients.<br>• A history of HTN, DM, and MI was more commonly observed in IS patients.<br>• Impaired LOC was predictive of ICH.<br>• Unilateral weakness or sensory loss was observed more frequently in IS patients.
Yamashita [26], 2011; Japan	Retrospective study	Prehospital and in-hospital	ICH, IS<br><br>ICH: 100<br>IS: 127	Uni- and multi-variable regression	ICH (median): 68<br>IS (median): 74	Within 6 h of onset	CT or MRI scan	• ACS<br>• AF<br>• Antithrombotics use<br>• DM<br>• Haemodialysis<br>• HTN<br>• Hyperlipidaemia<br>• Prior TIA, IS, and ICH<br>• Smoking	• Headache<br>• Neurological deficits using NIHSS items<br>• Seizures<br>• Vomiting	• SBP<br>• DBP<br>• ECG	• ICH patients were younger than IS patients.<br>• Patients with ICH were more likely to have a history of ICH and haemodialysis, while patients with IS had a higher likelihood of a history of ACS, AF, antithrombotic usage, and hyperlipidaemia.<br>• Headache, vomiting, impaired LOC, and seizures were more frequently observed in the ICH group.<br>• The NIHSS median score was higher in the ICH group (16 vs 9).<br>• The median BP was higher in the ICH group, including both SBP (190 vs 164 mm Hg) and DBP (102 vs 90 mm Hg).
Jin [27], 2016; China	Prospective study	Prehospital and in-hospital	ICH, IS, SAH, non-stroke<br><br>ICH: 689<br>IS: 797<br>SAH: 109<br>Non-stroke: 394	Uni- and multi-variable regression	All patients (mean): 66	NR (Evaluated in prehospital)	CT or MRI scan	• AF<br>• DM<br>• HTN	• Vomiting	• SBP<br>• DBP<br>• ECG<br>• HR	• The comparison was between two groups: ICH vs IS/TIA.<br>• Age <65 years was found to be associated with the ICH group.<br>• IS/TIA patients were more likely to have a history of AF and DM.<br>• Vomiting was a common presenting symptom in the ICH group.<br>• SBP ≥180 mm Hg and DBP ≥100 mm Hg were more frequently observed in ICH patients, while arrhythmia was a common finding in the IS/TIA group.

For peer review only

For peer review only - http://bmjopen.bmj.com/site/about/guidelines.xhtml

Uchida [28], 2018; Japan	Prospective study	Prehospital and in-hospital	Any stroke, ICH, LVO, SAH, non-stroke<br><br>DC: 1229<br>Any stroke: 203<br>ICH: 169<br>LVO: 104<br>SAH: 57<br>Non-stroke: 696<br><br>VC: 1007<br>Any stroke: 253<br>ICH: 183<br>LVO: 131<br>SAH: 50<br>Non-stroke: 390	Uni- and multi-variable regression	DC (median): 72<br><br>VC (median): 75	NR (Evaluated in prehospital)	CT or MRI scan	• Antiplatelet use<br>• DOAC use<br>• Prior IS, ICH, and SAH<br>• Smoking<br>• Warfarin use	• Anisocoria<br>• Aphasia<br>• Conjugate deviation<br>• Convulsion<br>• Dizziness<br>• Dysarthria<br>• Facial palsy<br>• Headache<br>• Impaired LOC<br>• Nausea or vomiting<br>• Numbness<br>• Paralysis of lower limbs<br>• Paralysis of upper limbs<br>• Sudden onset<br>• Symptoms improved after onset<br>• Symptoms progressed after onset<br>• Unilateral spatial neglect	• SBP<br>• DBP<br>• ECG	• Patients with ICH were less likely to have a history of IS.<br>• Improvement following the onset of symptoms was associated with a decreased probability of ICH.<br>• Headache, dysarthria, paralysis of the upper limbs, conjugate deviation, and impaired LOC were common presentations of ICH. However, most of these symptoms were not specific.<br>• SBP ≥165 mm Hg and DBP ≥95 mm Hg were more frequently observed in ICH patients, while the presence of arrhythmia was associated with the absence of ICH.
Geisler [29], 2021; Germany	Retrospective study	Prehospital and in-hospital	ICH, IS, TIA, SM<br><br>DC: 416<br>ICH: 32<br>IS: 224<br>TIA: 41<br>SM: 119<br><br>VC: 285<br>ICH: 33<br>IS: 252	Uni- and multi-variable regression	DC (mean):<br>ICH: 71.5<br>IS: 74.9<br><br>VC (mean):<br>ICH: 73.3<br>IS: 73.9	≤4 h or unknown	CT scanner in MSU	• AF<br>• HTN	• Neurological deficits using NIHSS items<br>• Seizures	• SBP<br>• DBP<br>• MAP	• In the derivation set (ICH vs IS), ICH patients were more likely to be male.<br>• AF was found less frequently in the derivation set for the ICH group.<br>• ICH patients had higher median NIHSS scores in both sets (≥15).<br>• A greater number of ICH patients showed a decrease in LOC.<br>• The majority of seizures were found in the SM group.<br>• A higher proportion of ICH patients exhibited SBP ≥180 mm Hg, DBP ≥110 mm Hg, and MAP ≥130 mm Hg.

For peer review only - http://bmjopen.bmj.com/site/about/guidelines.xhtml

Chiquete [30], 2021; Mexico	Prospective study	Prehospital and in-hospital	ICH, IS, SAH<br><br>ICH: 107<br>IS: 209<br>SAH: 53	Uni- and multi-variable regression	ICH (median): 60<br>IS (median): 68<br>SAH (median): 62	≤6 h in most cases	CT or MRI scan	• AF<br>• DM<br>• HTN<br>• Obesity<br>• Previous stroke	• Focal motor deficit<br>• Headache<br>• Impaired LOC<br>• Language or speech disorder<br>• Vomiting	NR	• A combination of focal motor deficit and a history of HTN performed the best in predicting ICH patients among different stroke subtypes.
Gioia [31], 2016; Canada	Retrospective study	Prehospital and in-hospital	ICH, IS, TIA, SM<br><br>ICH: 51<br>IS: 367<br>TIA: 117<br>SM: 416	Uni-variable	ICH (mean): 73.1<br>IS (mean): 73.3<br>TIA (mean): 72.4<br>SM (mean): 66.9	Median time: 75 min	Brain imaging	• AF<br>• CAD<br>• CHF<br>• DM type 2<br>• Dyslipidaemia<br>• HTN<br>• Prior stroke, ICH, and TIA	NR	• SBP<br>• DBP<br>• MAP	• SM patients were younger than patients with IS/TIA/ICH.<br>• In comparison to the other groups, ICH patients had higher rates of previous ICH.<br>• ICH patients had a higher mean prehospital SBP (172.3 ± 31.7 mm Hg) compared to IS (154.9 ± 26.5 mm Hg), TIA (153.3 ± 23.1 mm Hg), and SM (145.4 ± 25.4 mm Hg).<br>• ICH patients also had a higher mean prehospital DBP (95.8 ± 22.1 mm Hg) compared to IS (87.7 ± 17.1 mm Hg), TIA (86.6 ± 15.9 mm Hg), and SM (84.7 ± 16.8 mm Hg).<br>• The mean prehospital MAP of the ICH group was higher (122.0 ± 20.2 mm Hg) compared to IS (110.2 ± 18.9 mm Hg), TIA (108.6 ± 16.6 mm Hg), and SM (105.1 ± 18.0 mm Hg).<br>• During EMS transport, the ICH group maintained the highest mean SBP.

For peer review only

For peer review only - http://bmjopen.bmj.com/site/about/guidelines.xhtml

Irisawa [32], 2013; Japan	Retrospective study	Prehospital and in-hospital	ICH, IS, SAH, non-stroke<br><br>ICH: 6699<br>IS: 24,754<br>SAH: 1631<br>Non-stroke: 73,622	Uni- and multi-variable regression	All patients (mean): 63.1	NR (Evaluated in prehospital)	NR	NR	• Impaired LOC	• SBP	• Among patients with stroke with impaired LOC, elevated SBP (≥200 mm Hg) in the prehospital setting was strongly associated with a final diagnosis of ICH.
Andersson Hagiwara [33], 2018; Sweden	Retrospective study	Prehospital and in-hospital	ICH, IS<br><br>ICH: 172<br>IS: 1164	Uni-variable	ICH (mean, median): 74, 78<br>IS (mean, median): 77, 78	<24 h from symptoms onset	CT scan	• AF<br>• Angina pectoris<br>• CHF<br>• DM<br>• HTN<br>• Intermittent claudication<br>• Malignancy<br>• Prior amaurosis fugax<br>• Prior MI<br>• Prior stroke and TIA<br>• Sleep apnoea	Prehospital assessment:<br>• Arm weakness<br>• Facial droop<br>• Impaired LOC<br>• Leg weakness<br>• Numbness<br>• Speech disturbances<br><br>In-hospital assessment:<br>• Aphasia/ dysphasia<br>• Compromised balance<br>• Double vision<br>• Dysphagia<br>• Facial droop<br>• Headache<br>• Hemiparesis/ plegia<br>• Impaired LOC<br>• Numbness<br>• Syncope<br>• Vertigo	• SBP<br>• DBP<br>• ECG<br>• HR<br>• SPO_2	• ICH patients were slightly younger than patients with IS.<br>• In terms of past medical history, a history of previous TIA was more common in the IS group.<br>• Patients with ICH woke up with symptoms less frequently than IS patients.<br>• Leg weakness and decreased LOC were more frequently observed in ICH patients in the prehospital setting.<br>• During the in-hospital assessment, patients with ICH tended to present more often with headaches, syncope, and decreased LOC.<br>• Both SBP >200 mm Hg, and DBP >90 mm Hg were more frequently observed in ICH patients in both prehospital and in-hospital settings.

For peer review only - http://bmjopen.bmj.com/site/about/guidelines.xhtml

Puustjärvi [34], 2015; Finland	Retrospective-prospective study	In-hospital	PC-ICH, PC-IS<br><br>PC-ICH: 363<br>PC-IS: 190	Uni- and multi-variable regression	ICH (median): 68<br>IS (median): 65	NR (Evaluated in prehospital)	CT or MRI scan	• AF<br>• Antiplatelet use<br>• CAD<br>• CHF<br>• DM<br>• Dyslipidaemia<br>• HTN<br>• Warfarin use	• Deterioration<br>• Headache<br>• Neurological deficits using NIHSS items<br>• Seizures<br>• Sudden onset<br>• Vomiting	• SBP<br>• DBP<br>• GCS<br>• BGL	• Patients with PC-ICH were older than those with PC-IS.<br>• PC-ICH patients were more likely to have a history of HTN and warfarin use, and less likely to have a history of AF or dyslipidaemia than PC-IS patients.<br>• Headache and vomiting at the onset were more frequently observed in the PC-ICH group.<br>• Upon arrival at the ER, PC-ICH patients had higher median BP (177/92 vs 153/86 mm Hg) as well as higher median BGL (7.4 vs 6.8 mmol/L) than PC-IS patients.
Bustamante [35], 2021; Spain	Prospective study	In-hospital	ICH, IS<br><br>ICH: 35<br>IS: 154	Uni- and multi-variable regression	ICH (median): 82<br>IS (median): 81	Within 4.5 h after symptoms onset	CT scan	• AF<br>• Alcohol intake<br>• CAD<br>• DM<br>• Dyslipidaemia<br>• HTN<br>• PAD<br>• Previous stroke<br>• Smoking	• Neurological deficits using NIHSS items	• SBP<br>• DBP<br>• BGL	• Patients with ICH tended to be more frequently male than IS patients.<br>• Patients with IS had higher rates of AF compared to ICH patients.<br>• ICH patients tended to present with a more severe stroke than patients with IS (median NIHSS 17 vs 13).<br>• The median admission BP of ICH patients was higher than that of IS patients (160/84 vs 149/77 mm Hg).
Montaner [36], 2012; Spain	Cross-sectional study	In-hospital	ICH, IS<br><br>ICH: 139<br>IS: 776	Uni- and multi-variable regression	ICH (mean): 70.35<br>IS (mean): 73.05	Within the first 24 h after symptoms onset	CT scan	• AF<br>• Alcohol intake<br>• CAD<br>• DM<br>• Dyslipidaemia<br>• Embolic cardiopathy<br>• HTN<br>• PAD<br>• Prior IS and ICH<br>• Smoking	• Neurological deficits using NIHSS items	NR	• Patients with ICH were younger and more likely to be male than IS patients.<br>• ICH patients had a higher history of ICH and alcohol use, but a lower history of dyslipidaemia, PAD, CAD, and AF, which were more prevalent among IS patients.<br>• Patients with ICH experienced a more severe stroke than those with IS (median NIHSS 11 vs 6).

For peer review only

For peer review only - http://bmjopen.bmj.com/site/about/guidelines.xhtml

Rozanski [37], 2017; Germany	Prospective study	Prehospital and in-hospital	ICH, IS<br><br>ICH: 25<br>IS: 49	Uni-variable	ICH (mean): 69.4<br>IS (mean): 75.3	Median time: 63 min	CT scanner in MSU	• AF<br>• DM<br>• Dyslipidaemia<br>• HTN<br>• Smoking	• Neurological deficits using NIHSS items	• SBP	• Patients with ICH were younger than those with IS.<br>• Hypercholesterolaemia (dyslipidaemia) was less common among ICH patients compared to IS patients.<br>• Patients with ICH presented with more severe strokes than IS patients (median NIHSS 15 vs 6).<br>• ICH patients had a higher mean SBP than IS patients (204 vs 162 mm Hg).
Bustamante [38], 2017; Spain	Prospective study	In-hospital	ICH, IS<br><br>ICH: 174<br>IS: 941	Uni- and multi-variable regression	ICH (median): 70<br>IS (median): 76	<6 h from symptoms onset	Brain imaging	• AF<br>• Alcohol intake<br>• CAD<br>• DM<br>• Dyslipidaemia<br>• HTN<br>• Previous stroke<br>• Smoking	• Neurological deficits using NIHSS items	• SBP<br>• DBP<br>• BGL	• ICH patients were younger and more often male than IS patients.<br>• Patients with ICH were more likely to have a history of HTN and less likely to have a history of AF or CAD than IS patients.<br>• ICH patients presented with more severe neurological deficits compared to patients with IS (median NIHSS 12 vs 7).<br>• Patients with ICH had higher median SBP (174 vs 155 mm Hg), DBP (92 vs 82 mm Hg), and BGL (7.1 vs 6.7 mmol/L*) than patients with IS.
Llombart [39], 2016; Spain	Retrospective study	In-hospital	ICH, IS<br><br>ICH: 28<br>IS: 38	Uni- and multi-variable regression	ICH (mean): 76.03<br>IS (mean): 72.28	Within 6 h from the onset of symptoms	CT scan	• AF<br>• CAD<br>• DM<br>• Dyslipidaemia<br>• HTN<br>• Previous stroke<br>• Smoking	• Neurological deficits using NIHSS items	NR	• A history of HTN and CAD was more common among IS patients than ICH patients.

*Converted from mg/dL to mmol/L by dividing by 18.

Abbreviations: ACS, acute coronary syndrome; AF, atrial fibrillation; BGL, blood glucose level; BP, blood pressure; CAD, coronary artery disease; CHF, congestive heart failure; CT, computed tomography; DBP, diastolic blood pressure; DC, derivation cohort; DM, diabetes mellitus; DOAC, direct oral anticoagulant; ECG, electrocardiogram; EMS, emergency medical services; ER, emergency room; GCS, Glasgow Coma Scale; HR, heart rate; HTN, hypertension; ICH, intracerebral haemorrhage; IS, ischaemic stroke; LOC, level of consciousness; LVO, large vessel occlusion; MAP, mean arterial pressure; MI, myocardial infarction; MRI, magnetic resonance imaging; MSU, mobile stroke unit; NIHSS, National Institutes of Health Stroke Scale; NR, not reported; PAD, peripheral artery disease; PC-ICH, posterior circulation intracerebral haemorrhage; PC-IS, posterior circulation ischaemic stroke; SAH, subarachnoid haemorrhage; SBP, systolic blood pressure; SM, stroke mimic; SPO_2, peripheral oxygen saturation; TIA, transient ischaemic attack; VC, validation cohort.

For peer review only

For peer review only - http://bmjopen.bmj.com/site/about/guidelines.xhtml

Supplementary Table 2. Characteristics of the included studies for portable technologies.

Included study, year and country	Design	Technology and device employed	Study population	Sample size (Number)	Time to results	Comparator	User expertise	Diagnostic accuracy					Potential limitations
								SEN (95% CI)	SPE (95% CI)	AUC (95% CI)	PPV (95% CI)	NPV (95% CI)	
Persson [40], 2014; Sweden	Proof-of-concept study	Microwave<br><br>2 prototype systems	Patients with stroke, healthy controls	Study 1: 20<br><br>ICH: 9<br><br>IS: 11<br><br>Study 2: 90<br><br>ICH: 10<br><br>IS: 15<br><br>Healthy controls: 65	NR	CT scan	Staff members from the engineering, neurophysiology, and nursing departments	The first clinical study (study 1): ICH vs IS					• The development of brain oedema after IS may limit the diagnostic accuracy of this imaging technique.
								NR	NR	0.88	NR	NR	
								The second clinical study (study 2): ICH vs IS					
								NR	NR	0.85	NR	NR	
								The second clinical study (study 2): ICH vs healthy controls					
								NR	NR	0.87	NR	NR	
Kellner [41], 2018; USA	Pilot study	VIPS<br><br>Cerebrotech Visor	Suspected stroke patients, healthy controls	248<br><br>Severe strokes: 57 (2 with ICH ≥60 ml)<br><br>Minor strokes: 26 (10 with ICH <60 ml)<br><br>Healthy controls: 79<br><br>Other subjects: 86	30 sec	Brain imaging and prehospital stroke scales	Trained staff	Severe strokes vs minor strokes					• Metallic implants (intracranial or extracranial) may interfere with the device's ability to provide accurate measurements.<br>• No evidence was provided that VIPS could distinguish ICH from other conditions (e.g., IS).
								93% (83–98)	92% (75–99)	0.93 (0.85–0.97)	96% (88–99)	86% (70–94)	
								Severe strokes vs all subjects enrolled					
								93% (83–98)	87% (81–92)	0.93 (0.89–0.96)	70% (61–77)	98% (94–99)	

For peer review only

For peer review only - http://bmjopen.bmj.com/site/about/guidelines.xhtml

Seidel [42], 1995; Germany	Prospective study	Ultrasound<br><br>HP SONOS 1000	Suspected stroke patients	84<br><br>ICH: 15<br><br>IS/TIA (in MCA): 48<br><br>IS/TIA (in PCA): 4<br><br>Other subjects: 17	NR	CT scan	NR	TCCS as a first diagnostic modality for ICH					
								88%	96%	NR	88%	96%	• The detection of small ICH (<1×1cm) is limited.<br>• Brain tumours like gliomas, and haemorrhagic infarcts could be misdiagnosed as a spontaneous ICH.<br>• A total of 20% (17/84) of the enrolled patients could not be assessed due to inadequate bone windows.
Mäurer [43], 1998; Germany	Prospective study	Ultrasound<br><br>Siemens Sonoline Ellegra or Acuson 128 XP/4	Suspected stroke patients	151<br><br>ICH: 60<br><br>IS: 67<br><br>Non-stroke: 24	NR	CT scan	Neuroradiologist	ICH vs IS					
								94%	95%	NR	91%	95%	• The assessment of the frontal and parasagittal brain regions is challenging when the acoustic bone window is narrow.<br>• Increased echo density of the white matter in patients with cerebral microangiopathy may mimic the sonographic pattern of ICH.

For peer review only - http://bmjopen.bmj.com/site/about/guidelines.xhtml

Antipova [44], 2020; UK	Exploratory study	Ultrasound<br><br>SonoSite M-Turbo, Philips Sparq, or Philips CX50 ultrasound	Suspected stroke patients	107<br><br>ICH: 18<br><br>IS: 63<br><br>TIA: 13<br><br>SM: 13	20 min	CT scan	Expert sonographer	ICH vs IS/TIA/SM based on TCCS and clinical examination					• Inadequate acoustic windows (18/107) and user dependency may hinder the potential application.
								63%	99%	0.912 (0.829–0.996)	91%	92%	
Michelson [45], 2015; USA	Retrospective study	EEG<br><br>BrainScope	Patients with stroke, SM controls	183<br><br>HS: 17<br><br>IS: 31<br><br>SM controls: 135	10 min	CT or MRI scan	Expert EEG technologist	Classification result of HS					• Previous TIAs/strokes and the use of neuroactive medications could alter EEG findings.<br>• The device was not demonstrated to be capable of distinguishing between stroke subtypes.
								94.1%	50.4% (43.0–58.7)	NR	NR	NR	
Erani [46], 2020; USA	Proof-of-concept study	EEG<br><br>Quick-20	Suspected stroke patients	100<br><br>ICH: 7<br><br>IS: 43<br><br>TIA: 13<br><br>Other diagnoses: 37	13 min	CT or MRI scan	Trained staff	Detection of stroke/TIA					• This device was not evaluated for its ability to distinguish ICH from other diagnoses.
								65%	80%	78.2	NR	NR	

Abbreviations: AUC, area under curve; CI, confidence interval; CT, computed tomography; EEG, electroencephalography; HS, haemorrhagic stroke; ICH, intracerebral haemorrhage; IS, ischaemic stroke; MCA, middle cerebral artery; MRI, magnetic resonance imaging; NPV, negative predictive value; NR, not reported; PCA, posterior cerebral artery; PPV, positive predictive value; SEN, sensitivity; SM, stroke mimic; SPE, specificity; TCCS, transcranial colour-coded duplex sonography; TIA, transient ischaemic attack; UK, United Kingdom; USA, United States of America; VIPS, volumetric impedance phase shift spectroscopy.

For peer review only - http://bmjopen.bmj.com/site/about/guidelines.xhtml

Appendix 1. Preferred Reporting Items for Systematic reviews and Meta-Analyses extension for Scoping Reviews (PRISMA-ScR) Checklist

SECTION	ITEM	PRISMA-ScR CHECKLIST ITEM	REPORTED ON PAGE #
**TITLE**			
Title	1	Identify the report as a scoping review.	1
**ABSTRACT**			
Structured summary	2	Provide a structured summary that includes (as applicable): background, objectives, eligibility criteria, sources of evidence, charting methods, results, and conclusions that relate to the review questions and objectives.	2–3
**INTRODUCTION**			
Rationale	3	Describe the rationale for the review in the context of what is already known. Explain why the review questions/objectives lend themselves to a scoping review approach.	4–5
Objectives	4	Provide an explicit statement of the questions and objectives being addressed with reference to their key elements (e.g., population or participants, concepts, and context) or other relevant key elements used to conceptualize the review questions and/or objectives.	4–6
**METHODS**			
Protocol and registration	5	Indicate whether a review protocol exists; state if and where it can be accessed (e.g., a Web address); and if available, provide registration information, including the registration number.	5
Eligibility criteria	6	Specify characteristics of the sources of evidence used as eligibility criteria (e.g., years considered, language, and publication status), and provide a rationale.	6
Information sources*	7	Describe all information sources in the search (e.g., databases with dates of coverage and contact with authors to identify additional sources), as well as the date the most recent search was executed.	6
Search	8	Present the full electronic search strategy for at least 1 database, including any limits used, such that it could be repeated.	Appendix 2
Selection of sources of evidence†	9	State the process for selecting sources of evidence (i.e., screening and eligibility) included in the scoping review.	6
Data charting process‡	10	Describe the methods of charting data from the included sources of evidence (e.g., calibrated forms or forms that have been tested by the team before their use, and whether data charting was done independently or in duplicate) and any processes for obtaining and confirming data from investigators.	7
Data items	11	List and define all variables for which data were sought and any assumptions and simplifications made.	Appendix 2
Critical appraisal of individual sources of evidence§	12	If done, provide a rationale for conducting a critical appraisal of included sources of evidence; describe the methods used and how this information was used in any data synthesis (if appropriate).	N/A
Synthesis of results	13	Describe the methods of handling and summarizing the data that were charted.	7

St. Michael's
Inspired Care. Inspiring Science.

For peer review only - http://bmjopen.bmj.com/site/about/guidelines.xhtml

SECTION	ITEM	PRISMA-ScR CHECKLIST ITEM	REPORTED ON PAGE #
**RESULTS**			
Selection of sources of evidence	14	Give numbers of sources of evidence screened, assessed for eligibility, and included in the review, with reasons for exclusions at each stage, ideally using a flow diagram.	7, Figure 1
Characteristics of sources of evidence	15	For each source of evidence, present characteristics for which data were charted and provide the citations.	7–8
Critical appraisal within sources of evidence	16	If done, present data on critical appraisal of included sources of evidence (see item 12).	N/A
Results of individual sources of evidence	17	For each included source of evidence, present the relevant data that were charted that relate to the review questions and objectives.	Supplementary Tables 1 and 2
Synthesis of results	18	Summarize and/or present the charting results as they relate to the review questions and objectives.	7–13, 22
**DISCUSSION**			
Summary of evidence	19	Summarize the main results (including an overview of concepts, themes, and types of evidence available), link to the review questions and objectives, and consider the relevance to key groups.	13–18
Limitations	20	Discuss the limitations of the scoping review process.	18
Conclusions	21	Provide a general interpretation of the results with respect to the review questions and objectives, as well as potential implications and/or next steps.	18–19
**FUNDING**			
Funding	22	Describe sources of funding for the included sources of evidence, as well as sources of funding for the scoping review. Describe the role of the funders of the scoping review.	19

JBI = Joanna Briggs Institute; PRISMA-ScR = Preferred Reporting Items for Systematic reviews and Meta-Analyses extension for Scoping Reviews.
* Where *sources of evidence* (see second footnote) are compiled from, such as bibliographic databases, social media platforms, and Web sites.
† A more inclusive/heterogeneous term used to account for the different types of evidence or data sources (e.g., quantitative and/or qualitative research, expert opinion, and policy documents) that may be eligible in a scoping review as opposed to only studies. This is not to be confused with *information sources* (see first footnote).
‡ The frameworks by Arksey and O'Malley (6) and Levac and colleagues (7) and the JBI guidance (4, 5) refer to the process of data extraction in a scoping review as data charting.
§ The process of systematically examining research evidence to assess its validity, results, and relevance before using it to inform a decision. This term is used for items 12 and 19 instead of "risk of bias" (which is more applicable to systematic reviews of interventions) to include and acknowledge the various sources of evidence that may be used in a scoping review (e.g., quantitative and/or qualitative research, expert opinion, and policy document).

From: Tricco AC, Lillie E, Zarin W, O'Brien KK, Colquhoun H, Levac D, et al. PRISMA Extension for Scoping Reviews (PRISMAScR): Checklist and Explanation. Ann Intern Med. 2018;169:467–473. doi: 10.7326/M18-0850.

For peer review only - http://bmjopen.bmj.com/site/about/guidelines.xhtml

Appendix 2. Search strategies for the scoping review

1- The search strategy for clinical features

Database searched: MEDLINE (Ovid)

#	Query
1	exp Brain Ischemia/ or exp Stroke/ or exp Hemorrhagic Stroke/ or exp Cerebral Hemorrhage/ or exp Intracranial Hemorrhages/
2	Intracerebral hemorrhage.mp.
3	1 or 2
4	exp cerebrovascular accident/di
5	exp stroke/di
6	4 or 5
7	(recogni$ or identi$ or strati$ or different$ or probabilit$).mp.
8	3 and 7
9	6 or 8
10	exp Emergency Medical Services/ or Paramedic*.mp. or ambulance.mp. or triage.mp. or prehospital.mp. or pre hospital.mp. or EMS.mp. or emergency medical service*.mp.
11	Air Ambulances/
12	Emergency Service, Hospital/
13	Emergency Medicine/
14	Clinical feature.mp.
15	Stroke Score.mp.
16	Clinical differentiation.mp.
17	10 or 11 or 12 or 13 or 14 or 15 or 16
18	9 and 17
19	limit 18 to english language

For peer review only

Database searched: EMBASE (Ovid)

#	Query
1	exp Brain Ischemia/ or exp Stroke/ or exp Hemorrhagic Stroke/ or exp Cerebral Hemorrhage/ or exp Intracranial Hemorrhages/
2	Intracerebral hemorrhage.mp.
3	1 or 2
4	exp cerebrovascular accident/di [Diagnosis]
5	exp stroke/di
6	4 or 5
7	(recogni$ or identi$ or strati$ or distinguish$ or probabilit$).mp.
8	3 and 7
9	6 or 8
10	exp Emergency Medical Services/ or Paramedic*.mp. or ambulance.mp. or triage.mp. or prehospital.mp. or pre hospital.mp. or EMS.mp. or emergency medical service*.mp. or out-of-hospital.mp.
11	Air Ambulances/
12	Emergency Service, Hospital/
13	Stroke Score.mp.
14	Clinical differentiation.mp.
15	10 or 11 or 12 or 13 or 14
16	9 and 15
17	limit 16 to (human and english language)

For peer review only

Database searched: CENTRAL (Ovid)

#	Query
1	exp Brain Ischemia/ or exp Stroke/ or exp Hemorrhagic Stroke/ or exp Cerebral Hemorrhage/ or exp Intracranial Hemorrhages/
2	Intracerebral hemorrhage.mp.
3	1 or 2
4	exp cerebrovascular accident/di
5	exp stroke/di
6	4 or 5
7	(recogni$ or identi$ or strati$ or different$ or probabilit$).mp.
8	3 and 7
9	6 or 8
10	exp Emergency Medical Services/ or Paramedic*.mp. or ambulance.mp. or triage.mp. or prehospital.mp. or pre hospital.mp. or EMS.mp. or emergency medical service*.mp.
11	Air Ambulances/
12	Emergency Service, Hospital/
13	Emergency Medicine/
14	Clinical feature.mp.
15	Stroke Score.mp.
16	Clinical differentiation.mp.
17	10 or 11 or 12 or 13 or 14 or 15 or 16
18	9 and 17
19	limit 18 to english language

For peer review only

2- The search strategy for portable devices

Database searched: MEDLINE (Ovid)

#	Query
1	exp Brain Ischemia/ or exp Stroke/ or exp Hemorrhagic Stroke/ or exp Cerebral Hemorrhage/ or exp Intracranial Hemorrhages/
2	Intracerebral hemorrhage.mp.
3	1 or 2
4	(recogni$ or identi$ or strati$ or diagnos$ or detect$).mp.
5	exp Emergency Medical Services/ or Paramedic*.mp. or ambulance.mp. or triage.mp. or prehospital.mp. or pre hospital.mp. or EMS.mp. or emergency medical service*.mp.
6	Air Ambulances/
7	Emergency Service, Hospital/
8	(technology or spectroscopy or ultrasound or diagnostic imaging or imaging or transcranial doppler or radiofrequency or microwave or electroencephalography or device).mp.
9	5 or 6 or 7
10	3 and 4 and 8 and 9
11	limit 10 to (english language and humans)

For peer review only

Database searched: EMBASE (Ovid)

#	Query
1	exp Brain Ischemia/ or exp Stroke/ or exp Hemorrhagic Stroke/ or exp Cerebral Hemorrhage/ or exp Intracranial Hemorrhages/
2	Intracerebral hemorrhage.mp.
3	1 or 2
4	(recogni$ or identi$ or strati$ or diagnos$ or detect$).mp.
5	exp Emergency Medical Services/ or Paramedic*.mp. or ambulance.mp. or triage.mp. or prehospital.mp. or pre hospital.mp. or EMS.mp. or emergency medical service*.mp.
6	Air Ambulances/
7	Emergency Service, Hospital/
8	(technology or spectroscopy or ultrasound or diagnostic imaging or imaging or transcranial doppler or radiofrequency or microwave or electroencephalography or device).mp.
9	5 or 6 or 7
10	3 and 4 and 8 and 9
11	limit 10 to (english language and humans)

For peer review only

Database searched: CENTRAL (Ovid)

#	Query
1	exp Brain Ischemia/ or exp Stroke/ or exp Hemorrhagic Stroke/ or exp Cerebral Hemorrhage/ or exp Intracranial Hemorrhages/
2	Intracerebral hemorrhage.mp.
3	1 or 2
4	(recogni$ or identi$ or strati$ or diagnos$ or detect$).mp.
5	exp Emergency Medical Services/ or Paramedic*.mp. or ambulance.mp. or triage.mp. or prehospital.mp. or pre hospital.mp. or EMS.mp. or emergency medical service*.mp.
6	Air Ambulances/
7	Emergency Service, Hospital/
8	(technology or spectroscopy or ultrasound or diagnostic imaging or imaging or transcranial doppler or radiofrequency or microwave or electroencephalography or device).mp.
9	5 or 6 or 7
10	3 and 4 and 8 and 9
11	limit 10 to (english language and humans)

For peer review only
